# Supplementary material for: Effects of individual base-pairs on in vivo target search and destruction kinetics of bacterial small RNA
Source: Nat Commun. 2021 Feb 8;12:874. doi: 10.1038/s41467-021-21144-0 (PMC7870926; doi:10.1038/s41467-021-21144-0)
Supplement: Supplementary file 1 — Supplementary Information [file 41467_2021_21144_MOESM1_ESM.pdf]

Supplementary Information for:

Effects of individual base-pairs on in vivo target search and destruction kinetics of bacterial small RNA

Anustup Poddar[1], Muhammad S. Azam[2,3], Tunc Kayikcioglu[1], Maksym Bobrovskyy[2,4], Jichuan Zhang[1], Xiangqian Ma[2], Piyush Labhsetwar[5,6], Jingyi Fei[7], Digvijay Singh[1,8], Zaida Luthey-Schulten[5], Carin K. Vanderpool[2,*] and Taekjip Ha[1,9,*]

[1]Department of Biophysics and Biophysical Chemistry, Johns Hopkins University School of Medicine, Baltimore, Maryland 21205

[2]Department of Microbiology, University of Illinois at Urbana-Champaign, Urbana, Illinois 61801

[3]Present Address: Department of Biochemistry and Molecular Biology, University of Chicago, Chicago, Illinois 60637

[4]Present Address: Department of Microbiology, University of Chicago, Chicago, Illinois 60637

[5]Department of Chemistry, University of Illinois at Urbana-Champaign, Urbana, Illinois 61801

[6]Present Address: The Land Institute, Salina, Kansas 67401

[7]Department of Biochemistry and Molecular Biology, University of Chicago, Chicago, Illinois 60637

[8]Present Address: Division of Biological Sciences, University of California, San Diego, San Diego, California 92093

[9]Howard Hughes Medical Institute, Baltimore, Maryland 21205

* Corresponding authors

Taekjip Ha, Ph.D. (tjha@jhu.edu)

Carin K. Vanderpool, Ph.D. (cvanderp@illinois.edu)

This document includes:

Supplementary Figures 1-54

Supplementary Tables 1-4

Supplementary Note 1

References

Supplementary Figures

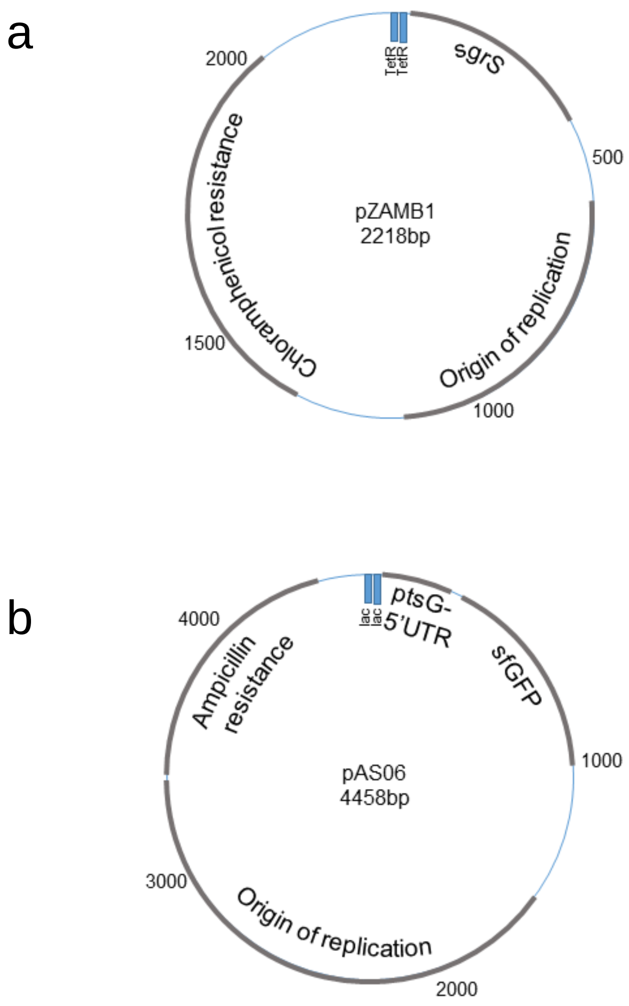

Supplementary Figure 1. Plasmids used for Sort-Seq. (a) pZAMB1 plasmid containing wild-type *sgrS* sequence, **(b)** pAS06 plasmid containing *ptsG* 5’ UTR fused to superfolder GFP (sfGFP).

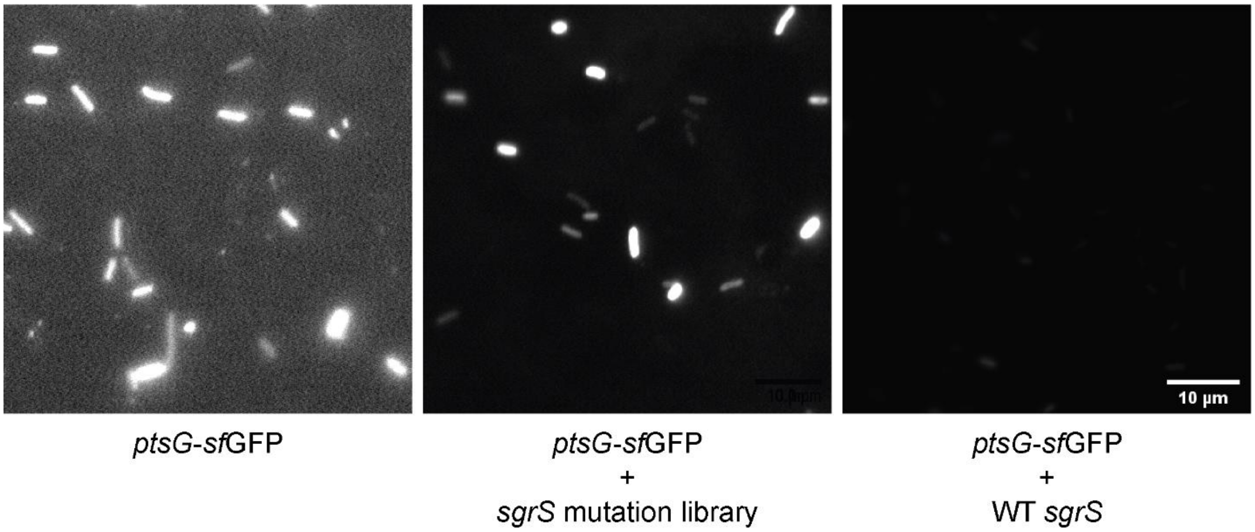

Supplementary Figure 2. Verification of target-reporter system. Epifluorescence imaging of cells expressing only ptsG-*sf*GFP, ptsG-*sf*GFP with WT *sgrS* and ptsG-*sf*GFP with *sgrS* mutation library. Each experiment was performed independently 2 times. Scale bar is 10 μm and applies to all the panels.

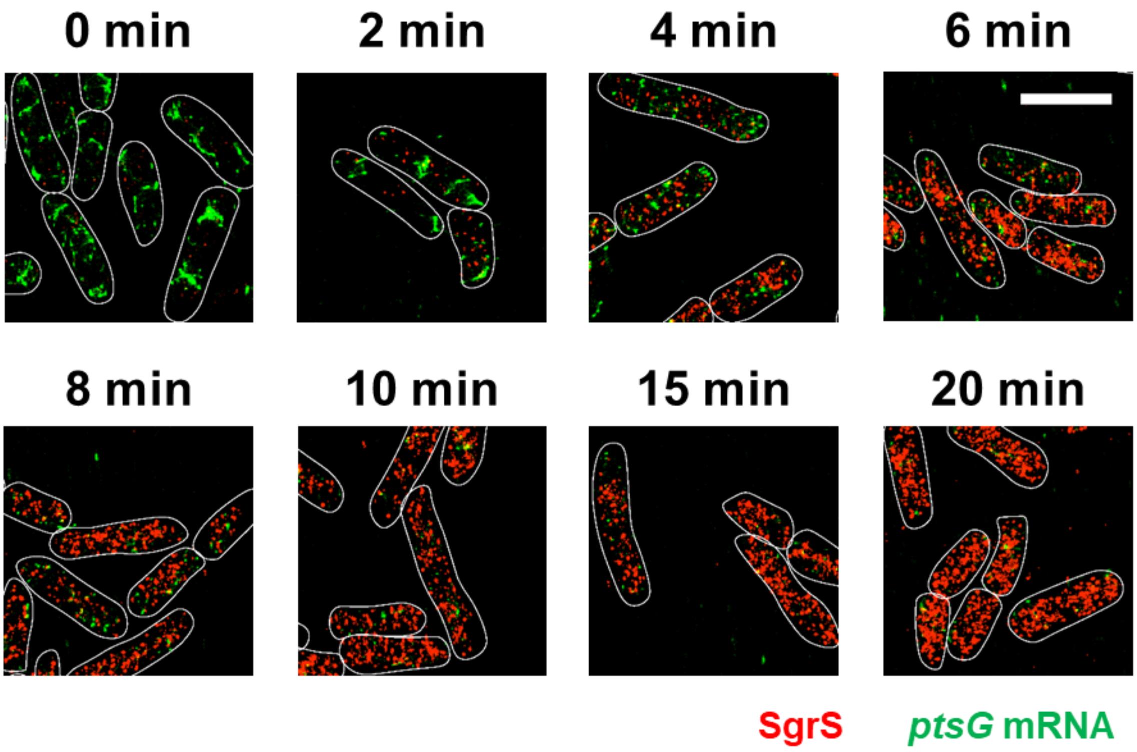

Supplementary Figure 3. 3D super-resolution images of SgrS (red) and *ptsG* mRNA (green) in the wild-type SgrS strain projected on 2D planes. The panels show the multi-color images of WT SgrS cells before (0 min) and 2, 4, 6, 8, 10, 15, 20 min after αMG (non-metabolizable sugar analog) induction. Each experiment was performed independently 2 times. White lines denote cell boundaries. Scale bar is 2 μm and applies to all the panels.

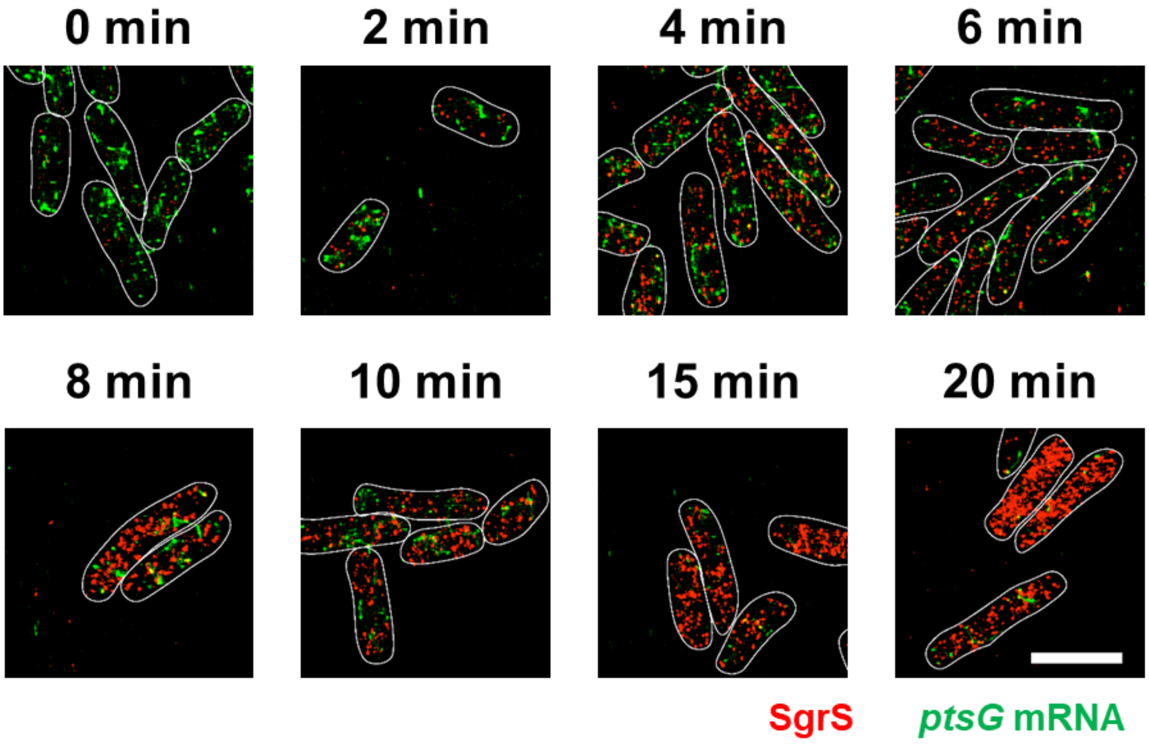

Supplementary Figure 4. 3D super-resolution images of SgrS (red) and *ptsG* mRNA (green) in the SgrS A177U mutant strain projected on 2D planes. The panels show the multi-color images of SgrS A177U cells before (0 min) and 2, 4, 6, 8, 10, 15, 20 min after αMG (non-metabolizable sugar analog) induction. Each experiment was performed independently 2 times. White lines denote cell boundaries. Scale bar is 2 μm and applies to all the panels.

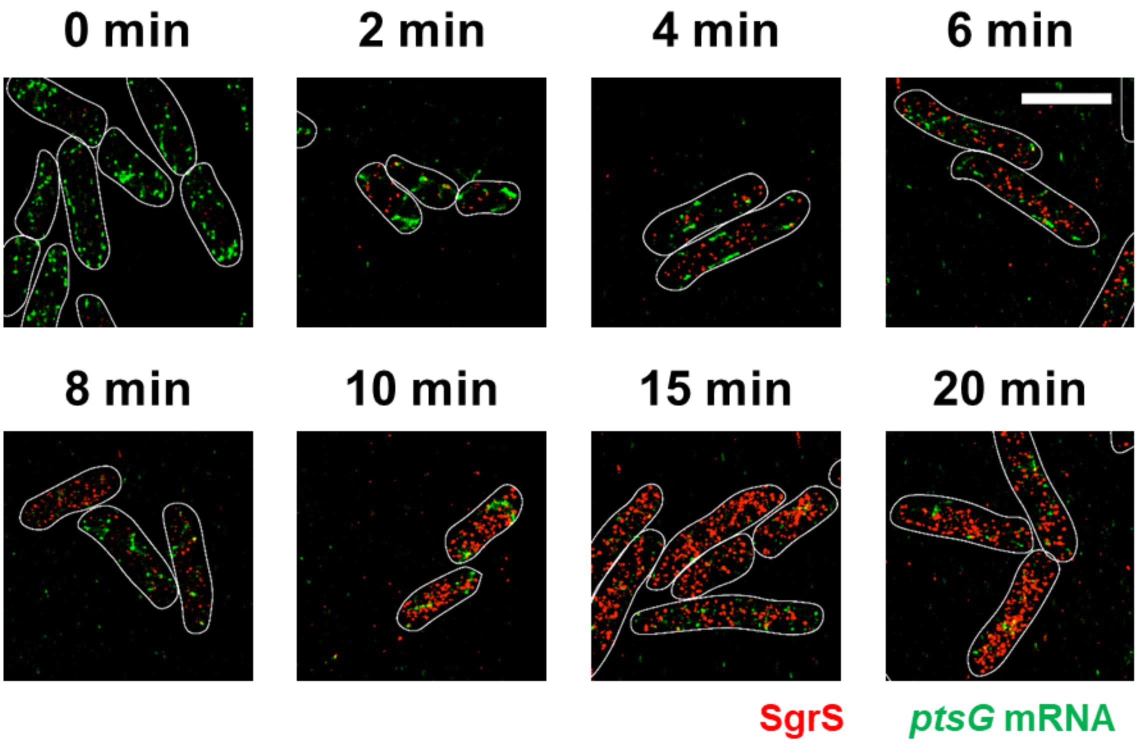

Supplementary Figure 5. 3D super-resolution images of SgrS (red) and *ptsG* mRNA (green) in the SgrS G178A mutant strain projected on 2D planes. The panels show the multi-color images of SgrS G178A cells before (0 min) and 2, 4, 6, 8, 10, 15, 20 min after αMG (non-metabolizable sugar analog) induction. Each experiment was performed independently 2 times. White lines denote cell boundaries. Scale bar is 2 μm and applies to all the panels.

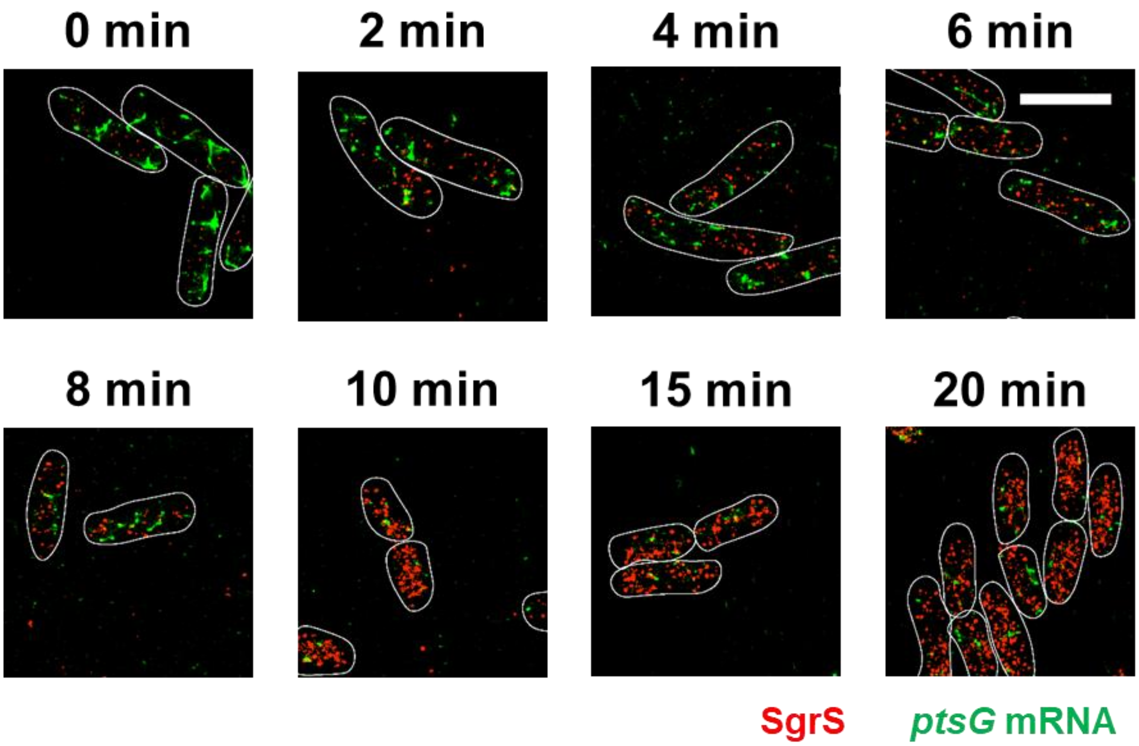

Supplementary Figure 6. 3D super-resolution images of SgrS (red) and *ptsG* mRNA (green) in the SgrS G178U mutant strain projected on 2D planes. The panels show the multi-color images of SgrS G178U cells before (0 min) and 2, 4, 6, 8, 10, 15, 20 min after αMG (non-metabolizable sugar analog) induction. Each experiment was performed independently 2 times. White lines denote cell boundaries. Scale bar is 2 μm and applies to all the panels.

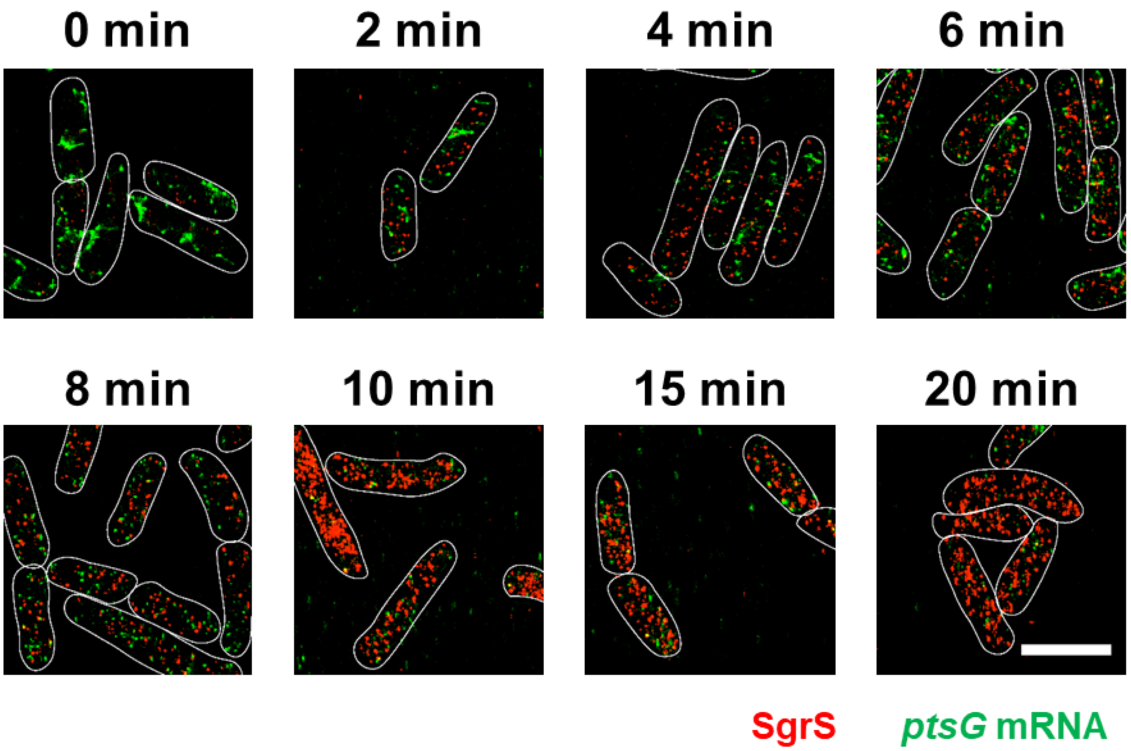

Supplementary Figure 7. 3D super-resolution images of SgrS (red) and *ptsG* mRNA (green) in the SgrS U181A mutant strain projected on 2D planes. The panels show the multi-color images of SgrS U181A cells before (0 min) and 2, 4, 6, 8, 10, 15, 20 min after αMG (non-metabolizable sugar analog) induction. Each experiment was performed independently 2 times. White lines denote cell boundaries. Scale bar is 2 μm and applies to all the panels.

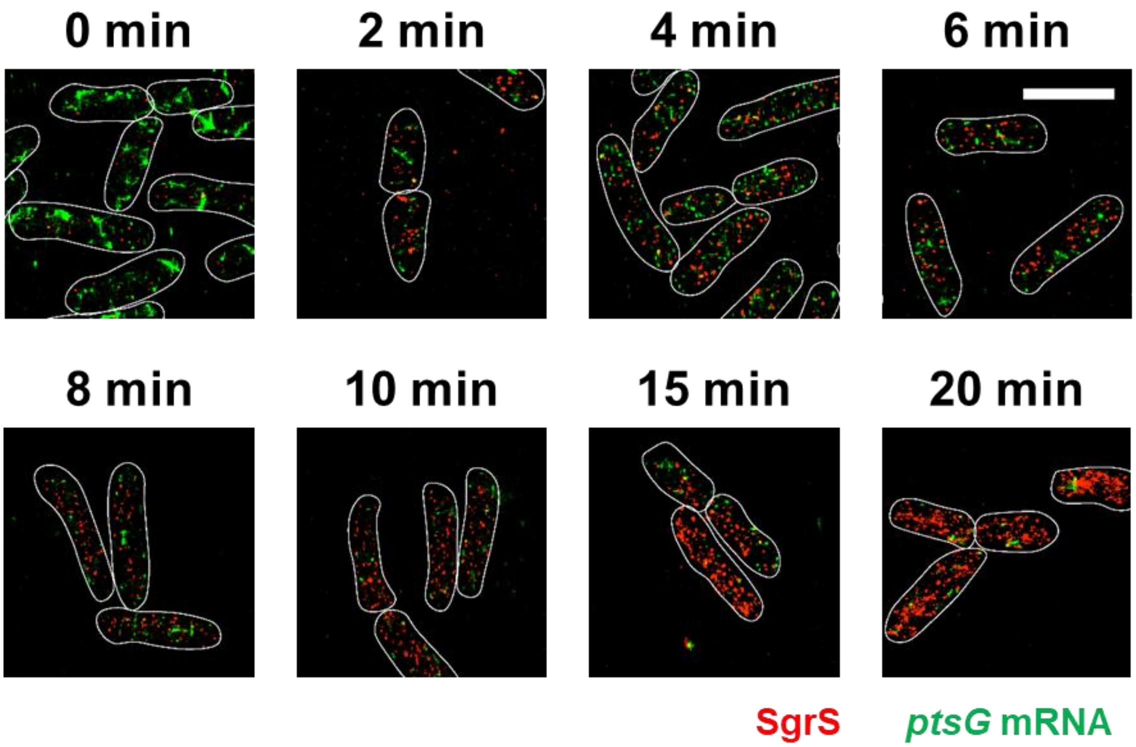

Supplementary Figure 8. 3D super-resolution images of SgrS (red) and *ptsG* mRNA (green) in the SgrS U182A mutant strain projected on 2D planes. The panels show the multi-color images of SgrS U182A cells before (0 min) and 2, 4, 6, 8, 10, 15, 20 min after αMG (non-metabolizable sugar analog) induction. Each experiment was performed independently 2 times. White lines denote cell boundaries. Scale bar is 2 μm and applies to all the panels.

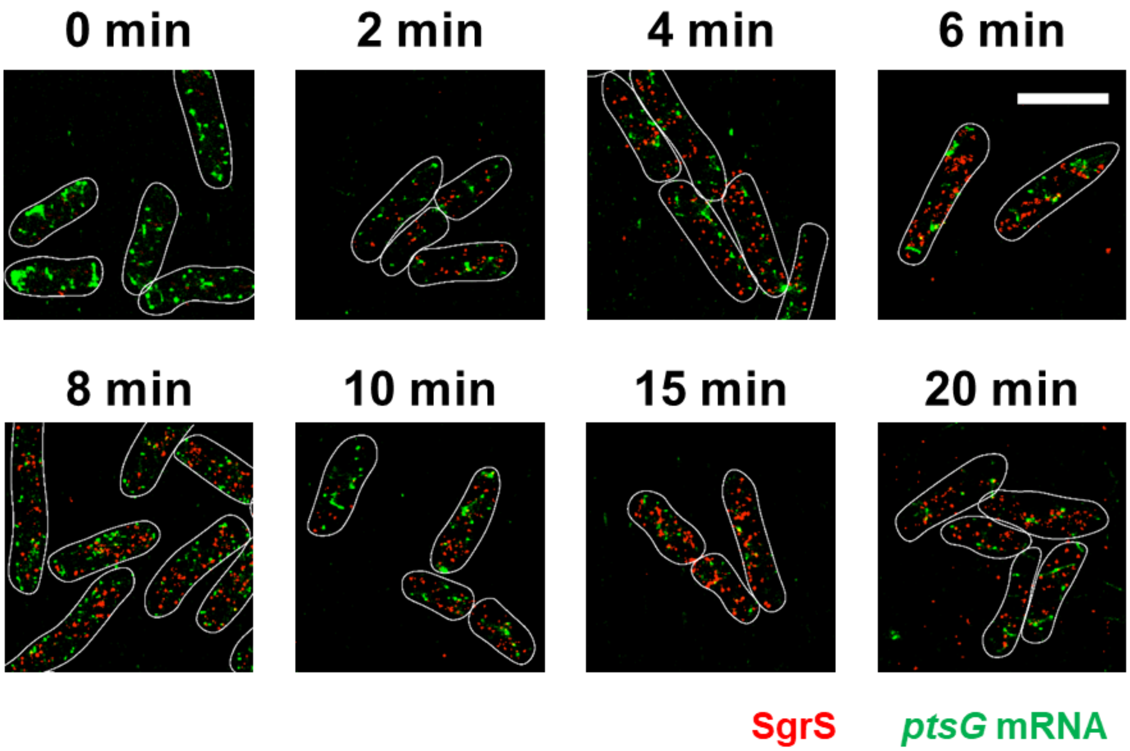

Supplementary Figure 9. 3D super-resolution images of SgrS (red) and *ptsG* mRNA (green) in the SgrS G184A mutant strain projected on 2D planes. The panels show the multi-color images of SgrS G184A cells before (0 min) and 2, 4, 6, 8, 10, 15, 20 min after αMG (non-metabolizable sugar analog) induction. Each experiment was performed independently 2 times. White lines denote cell boundaries. Scale bar is 2 μm and applies to all the panels.

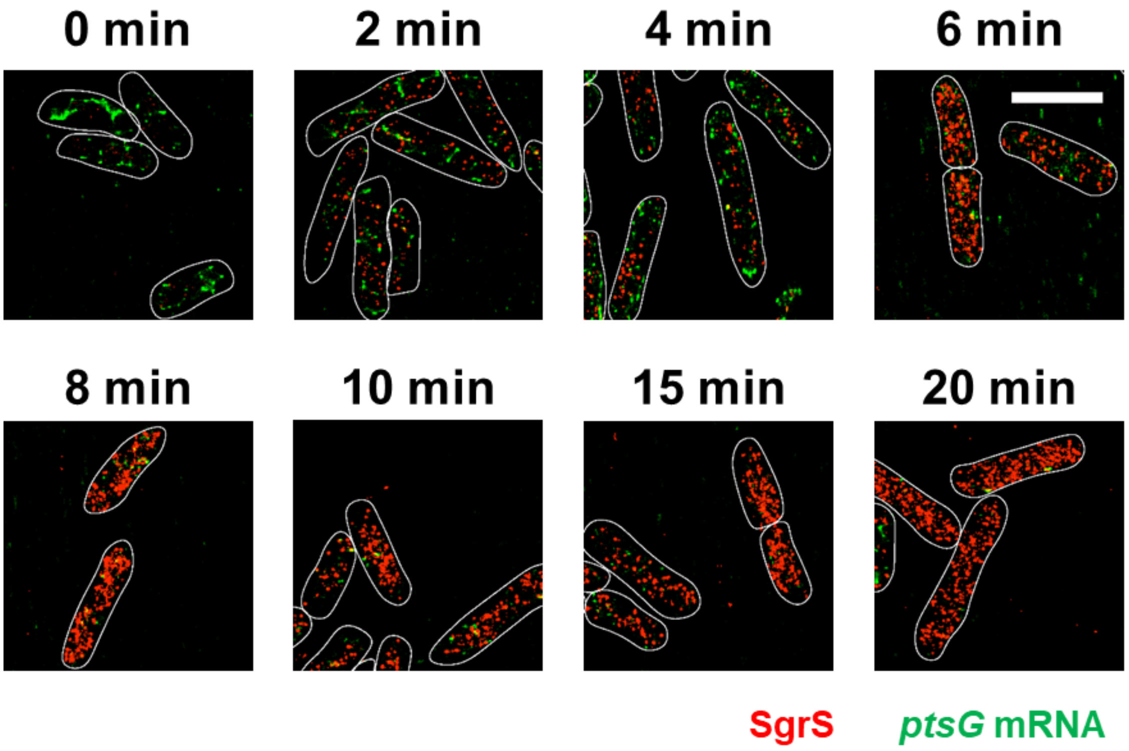

Supplementary Figure 10. 3D super-resolution images of SgrS (red) and *ptsG* mRNA (green) in the SgrS G184A-C195U mutant strain projected on 2D planes. The panels show the multi-color images of SgrS G184A-C195U cells before (0 min) and 2, 4, 6, 8, 10, 15, 20 min after αMG (non-metabolizable sugar analog) induction. Each experiment was performed independently 2 times. White lines denote cell boundaries. Scale bar is 2 μm and applies to all the panels.

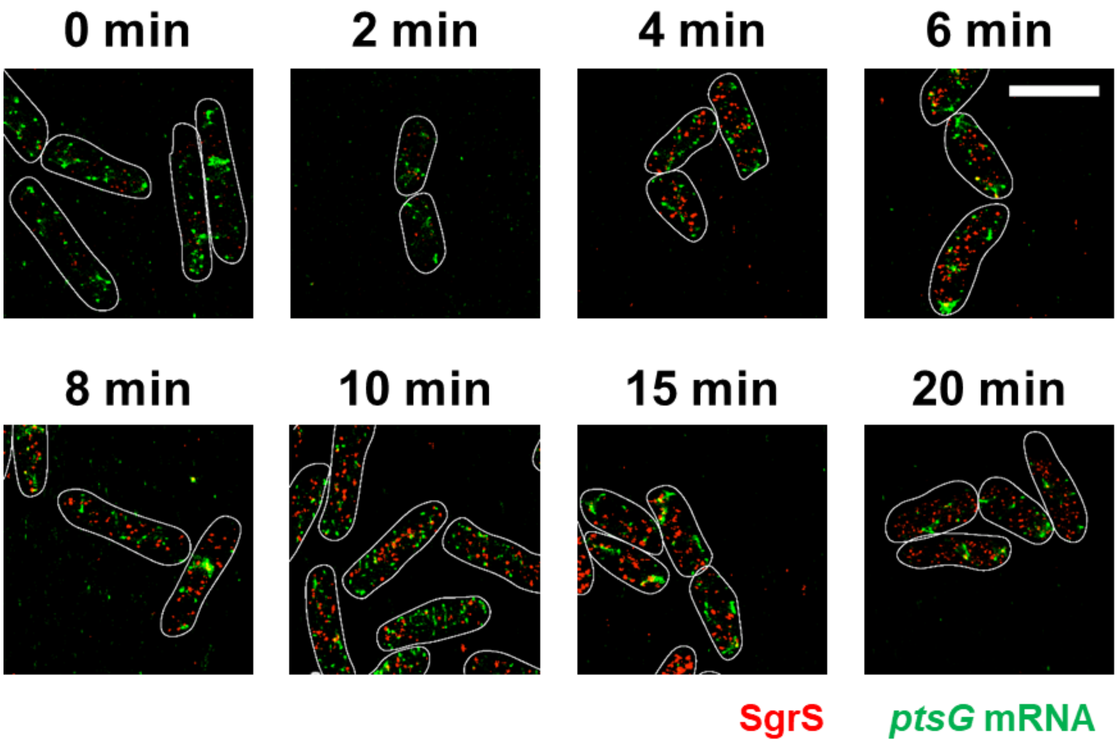

Supplementary Figure 11. 3D super-resolution images of SgrS (red) and *ptsG* mRNA (green) in the SgrS G215A mutant strain projected on 2D planes. The panels show the multi-color images of SgrS G215A cells before (0 min) and 2, 4, 6, 8, 10, 15, 20 min after αMG (non-metabolizable sugar analog) induction. Each experiment was performed independently 2 times. White lines denote cell boundaries. Scale bar is 2 μm and applies to all the panels.

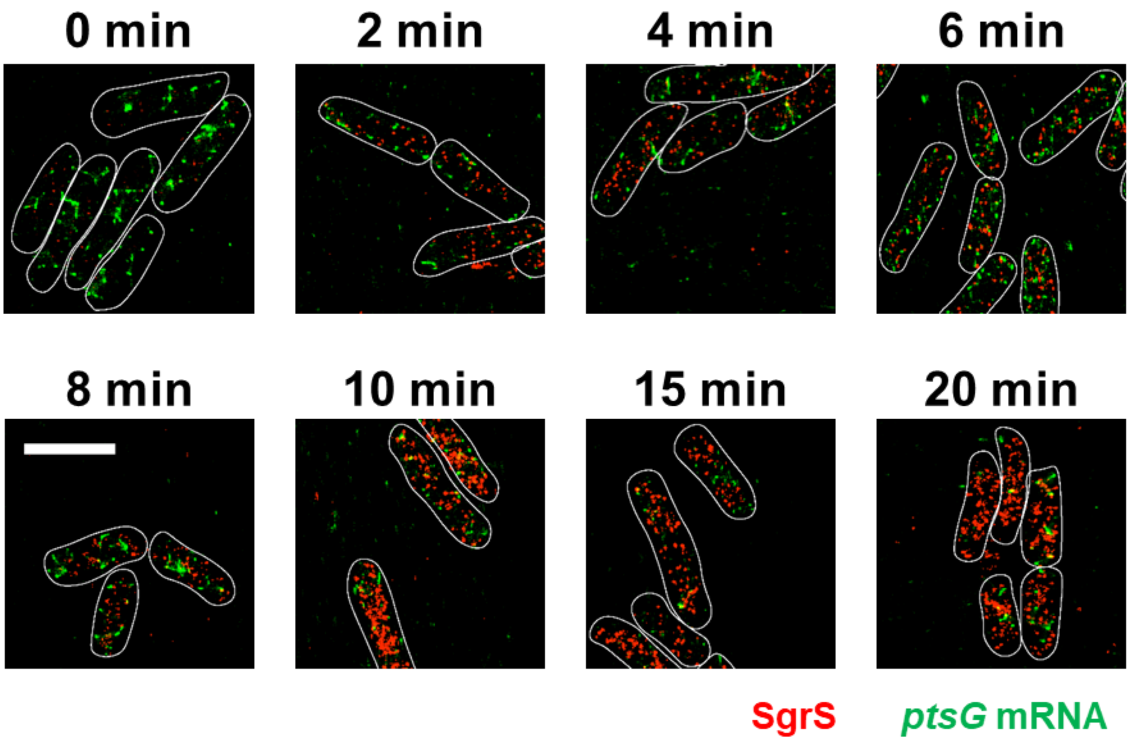

Supplementary Figure 12. 3D super-resolution images of SgrS (red) and *ptsG* mRNA (green) in the SgrS U224A mutant strain projected on 2D planes. The panels show the multi-color images of SgrS U224A cells before (0 min) and 2, 4, 6, 8, 10, 15, 20 min after αMG (non-metabolizable sugar analog) induction. Each experiment was performed independently 2 times. White lines denote cell boundaries. Scale bar is 2 μm and applies to all the panels.

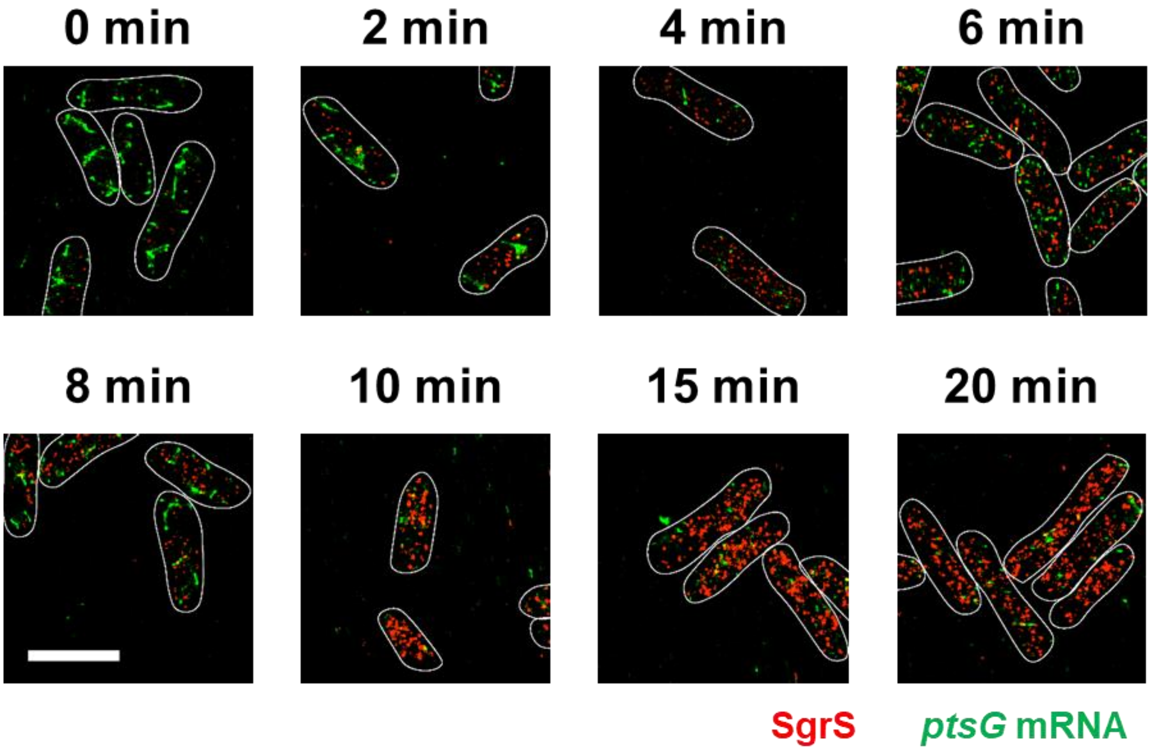

Supplementary Figure 13. 3D super-resolution images of SgrS (red) and *ptsG* mRNA (green) in the SgrS U224G mutant strain projected on 2D planes. The panels show the multi-color images of SgrS U224G cells before (0 min) and 2, 4, 6, 8, 10, 15, 20 min after αMG (non-metabolizable sugar analog) induction. Each experiment was performed independently 2 times. White lines denote cell boundaries. Scale bar is 2 μm and applies to all the panels.

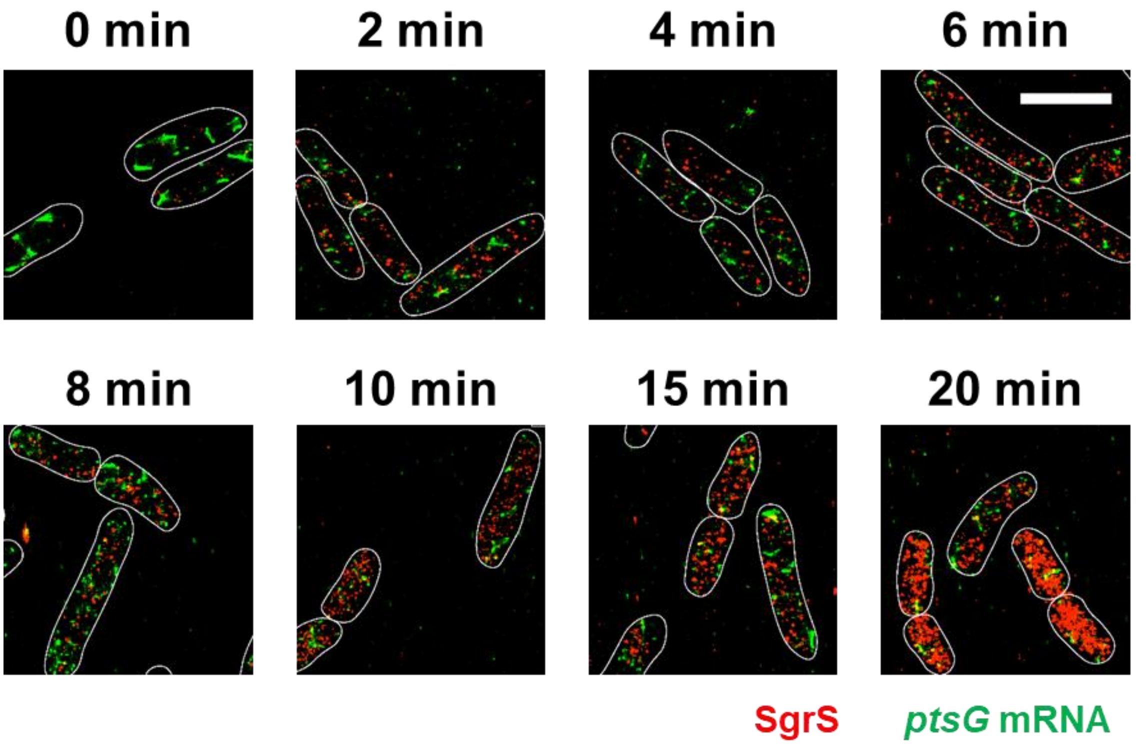

Supplementary Figure 14. 3D super-resolution images of SgrS (red) and *ptsG* mRNA (green) in the wild-type SgrS RNase E mutant strain projected on 2D planes. The panels show the multi-color images of WT SgrS RNase E mutant cells before (0 min) and 2, 4, 6, 8, 10, 15, 20 min after αMG (non-metabolizable sugar analog) induction. Each experiment was performed independently 2 times. White lines denote cell boundaries. Scale bar is 2 μm and applies to all the panels.

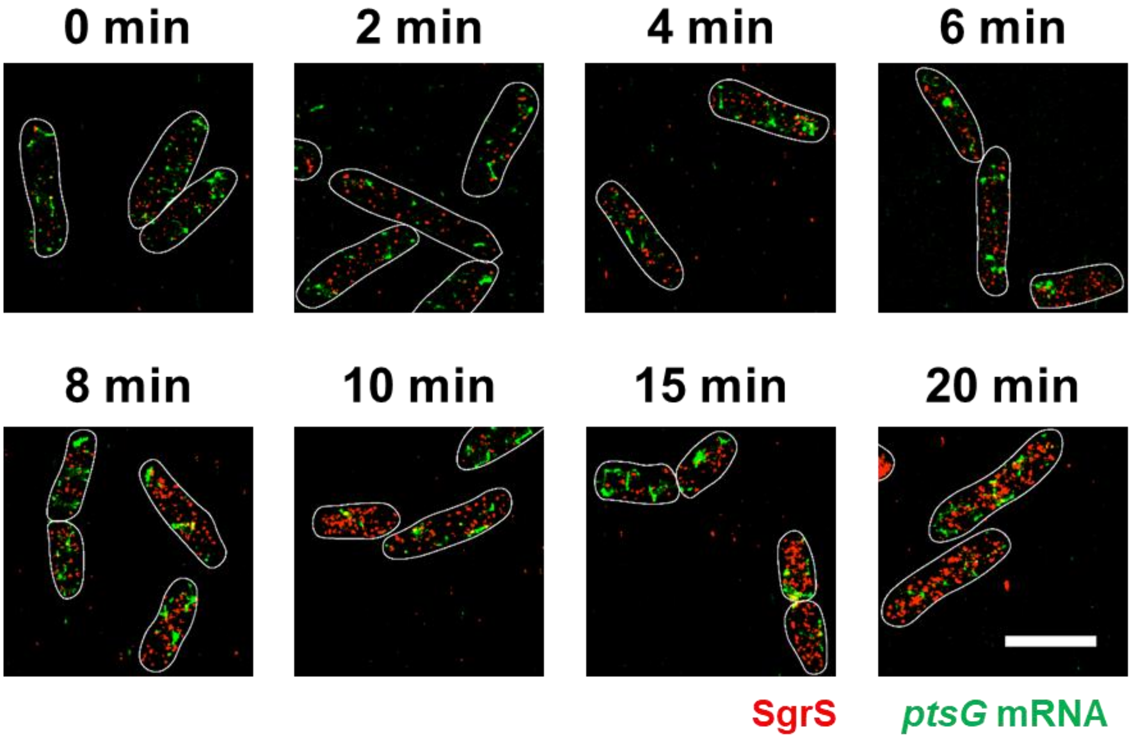

Supplementary Figure 15. 3D super-resolution images of SgrS (red) and *ptsG* mRNA (green) in the SgrS A177U RNase E mutant strain projected on 2D planes. The panels show the multi-color images of SgrS A177U RNase E mutant cells before (0 min) and 2, 4, 6, 8, 10, 15, 20 min after αMG (non-metabolizable sugar analog) induction. Each experiment was performed independently 2 times. White lines denote cell boundaries. Scale bar is 2 μm and applies to all the panels.

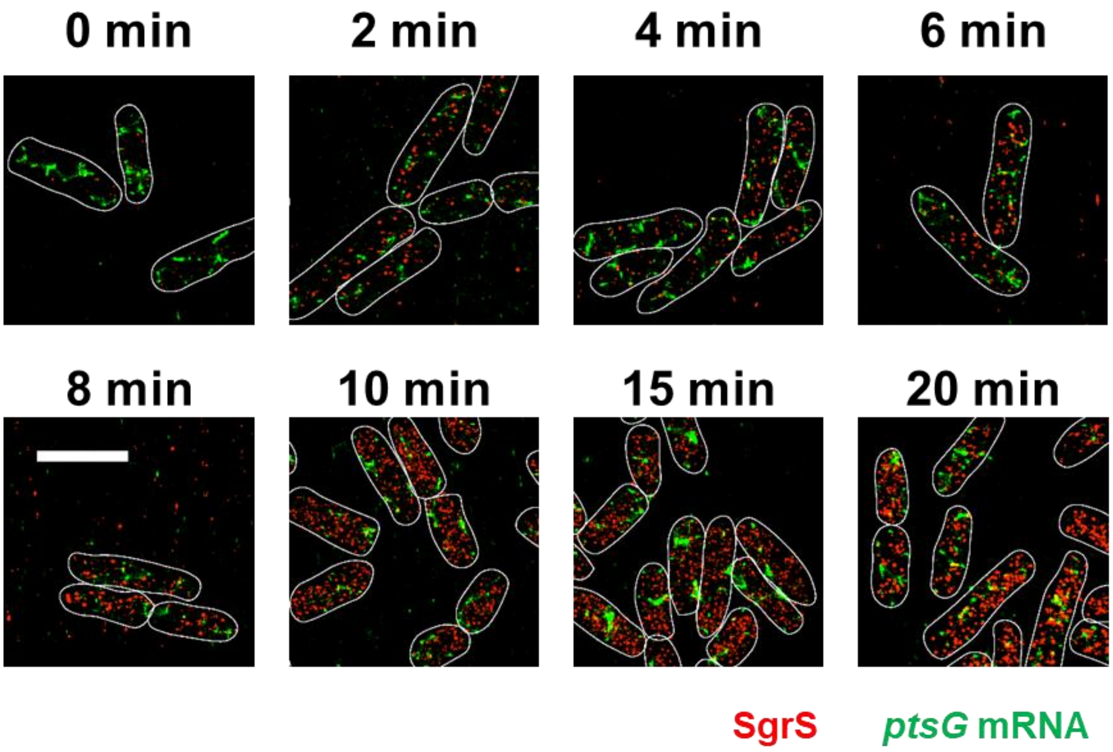

Supplementary Figure 16. 3D super-resolution images of SgrS (red) and *ptsG* mRNA (green) in the SgrS G178A RNase E mutant strain projected on 2D planes. The panels show the multi-color images of SgrS G178A RNase E mutant cells before (0 min) and 2, 4, 6, 8, 10, 15, 20 min after αMG (non-metabolizable sugar analog) induction. Each experiment was performed independently 2 times. White lines denote cell boundaries. Scale bar is 2 μm and applies to all the panels.

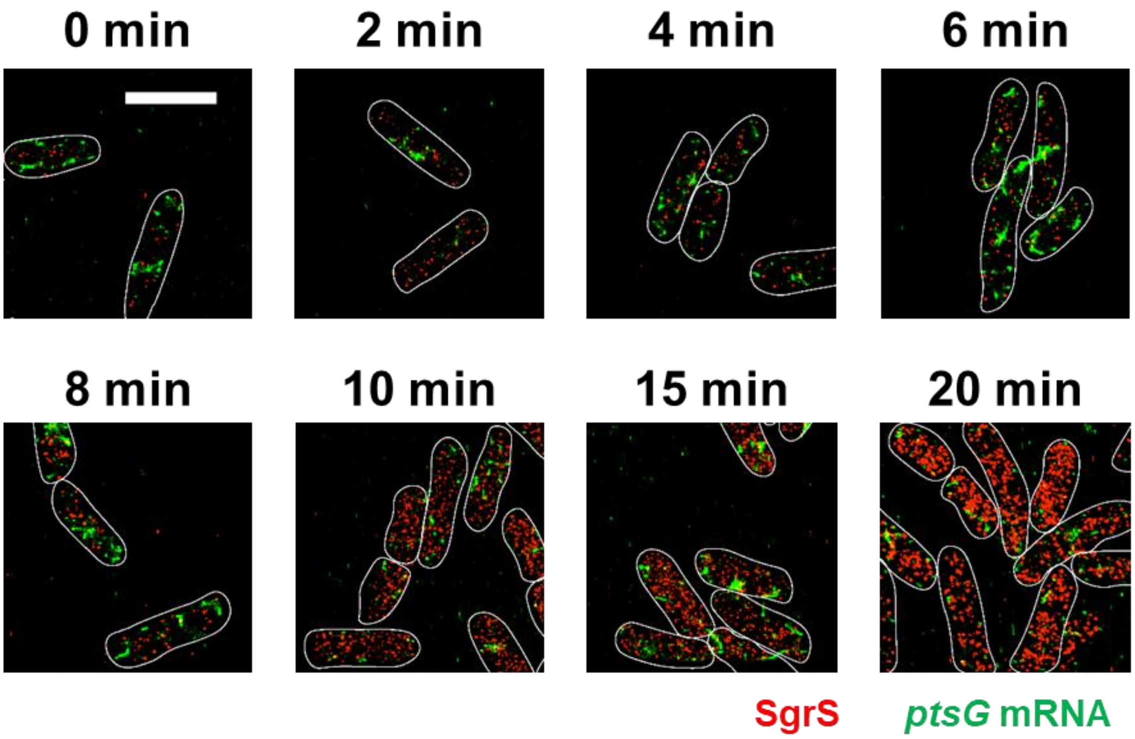

Supplementary Figure 17. 3D super-resolution images of SgrS (red) and *ptsG* mRNA (green) in the SgrS G178U RNase E mutant strain projected on 2D planes. The panels show the multi-color images of SgrS G178U RNase E mutant cells before (0 min) and 2, 4, 6, 8, 10, 15, 20 min after αMG (non-metabolizable sugar analog) induction. Each experiment was performed independently 2 times. White lines denote cell boundaries. Scale bar is 2 μm and applies to all the panels.

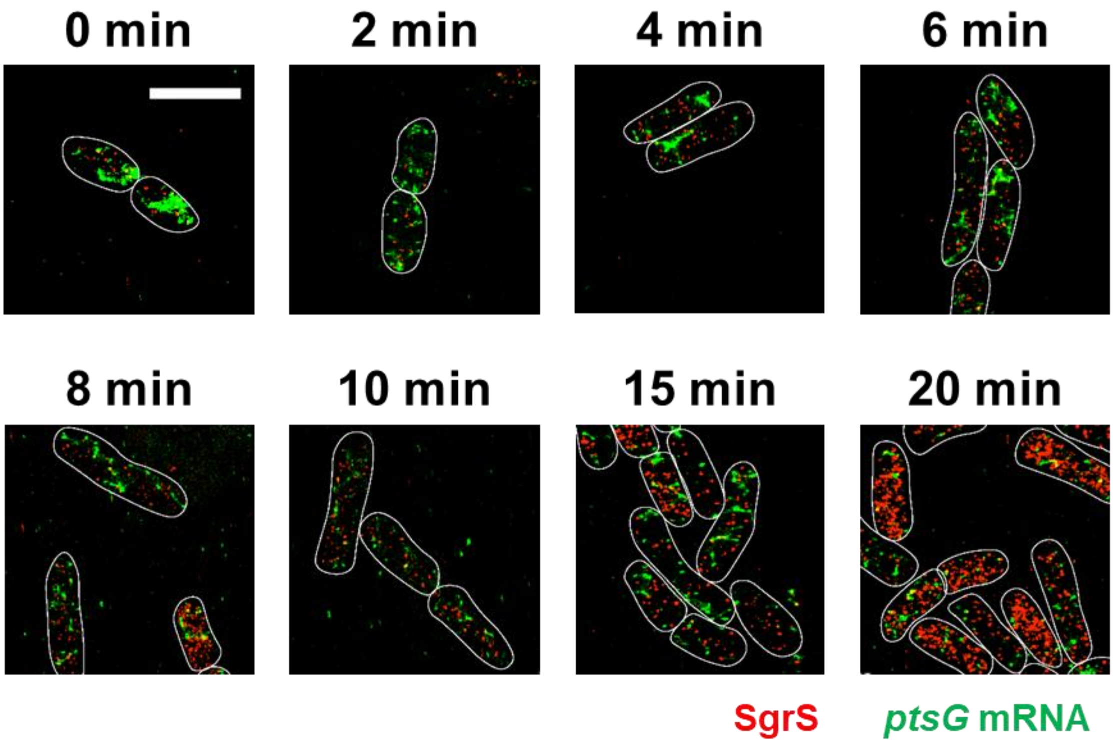

Supplementary Figure 18. 3D super-resolution images of SgrS (red) and *ptsG* mRNA (green) in the SgrS U181A RNase E mutant strain projected on 2D planes. The panels show the multi-color images of SgrS U181A RNase E mutant cells before (0 min) and 2, 4, 6, 8, 10, 15, 20 min after αMG (non-metabolizable sugar analog) induction. Each experiment was performed independently 2 times. White lines denote cell boundaries. Scale bar is 2 μm and applies to all the panels.

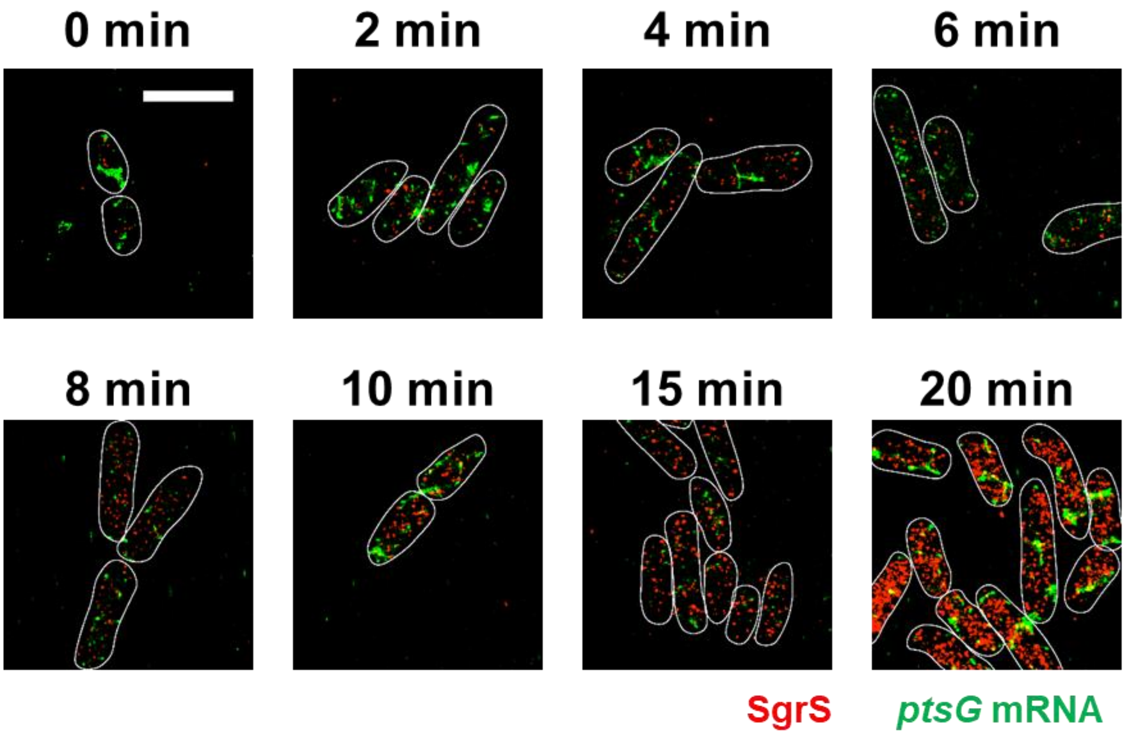

Supplementary Figure 19. 3D super-resolution images of SgrS (red) and *ptsG* mRNA (green) in the SgrS U182A RNase E mutant strain projected on 2D planes. The panels show the multi-color images of SgrS U182A RNase E mutant cells before (0 min) and 2, 4, 6, 8, 10, 15, 20 min after αMG (non-metabolizable sugar analog) induction. Each experiment was performed independently 2 times. White lines denote cell boundaries. Scale bar is 2 μm and applies to all the panels.

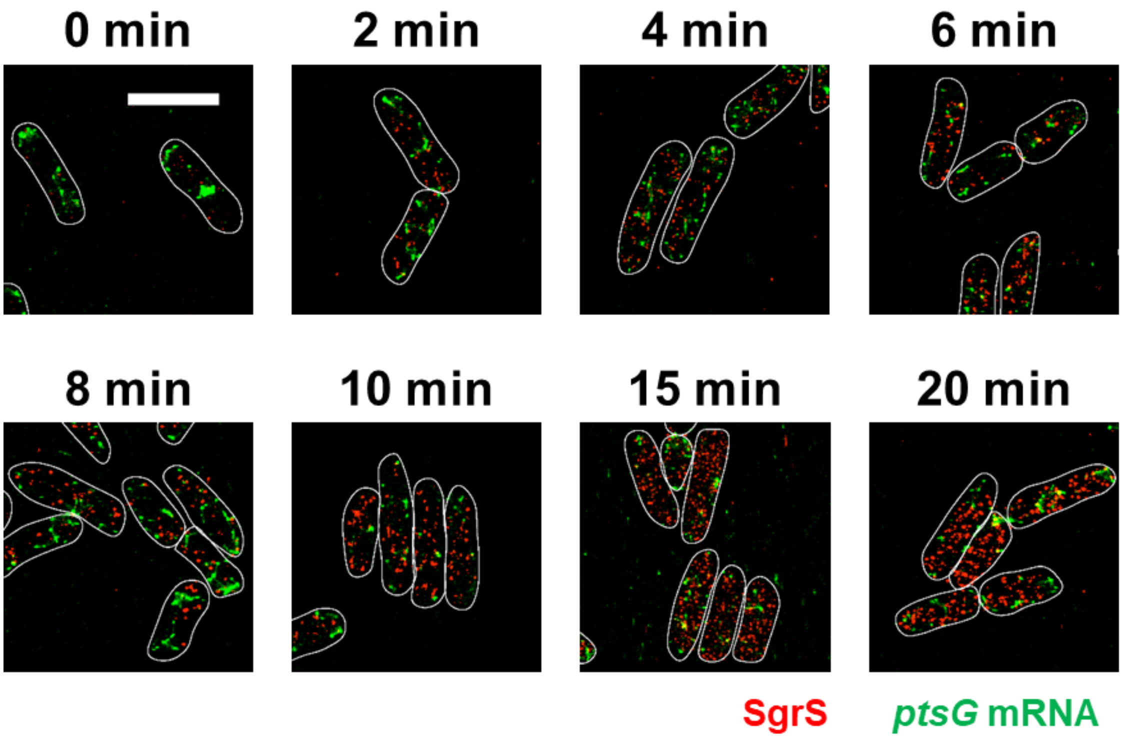

Supplementary Figure 20. 3D super-resolution images of SgrS (red) and *ptsG* mRNA (green) in the SgrS G184A RNase E mutant strain projected on 2D planes. The panels show the multi-color images of SgrS G184A RNase E mutant cells before (0 min) and 2, 4, 6, 8, 10, 15, 20 min after αMG (non-metabolizable sugar analog) induction. Each experiment was performed independently 2 times. White lines denote cell boundaries. Scale bar is 2 μm and applies to all the panels.

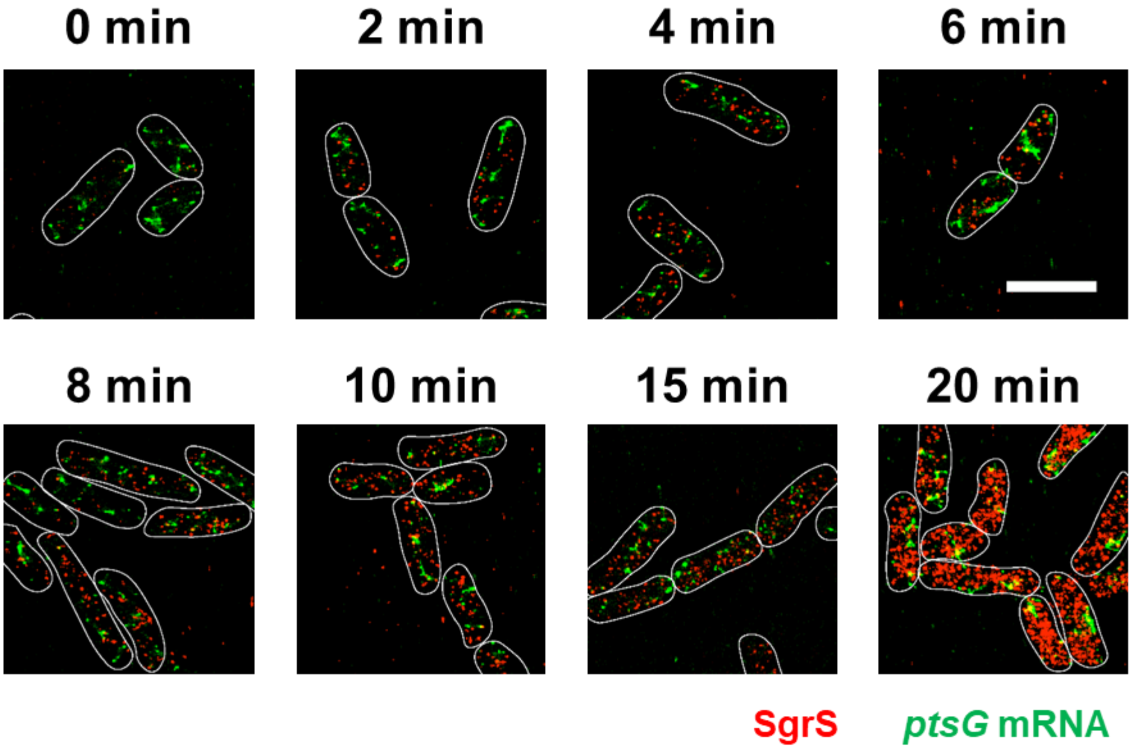

Supplementary Figure 21. 3D super-resolution images of SgrS (red) and *ptsG* mRNA (green) in the SgrS G184A-C195U RNase E mutant strain projected on 2D planes. The panels show the multi-color images of SgrS G184A-C195U RNase E mutant cells before (0 min) and 2, 4, 6, 8, 10, 15, 20 min after αMG (non-metabolizable sugar analog) induction. Each experiment was performed independently 2 times. White lines denote cell boundaries. Scale bar is 2 μm and applies to all the panels.

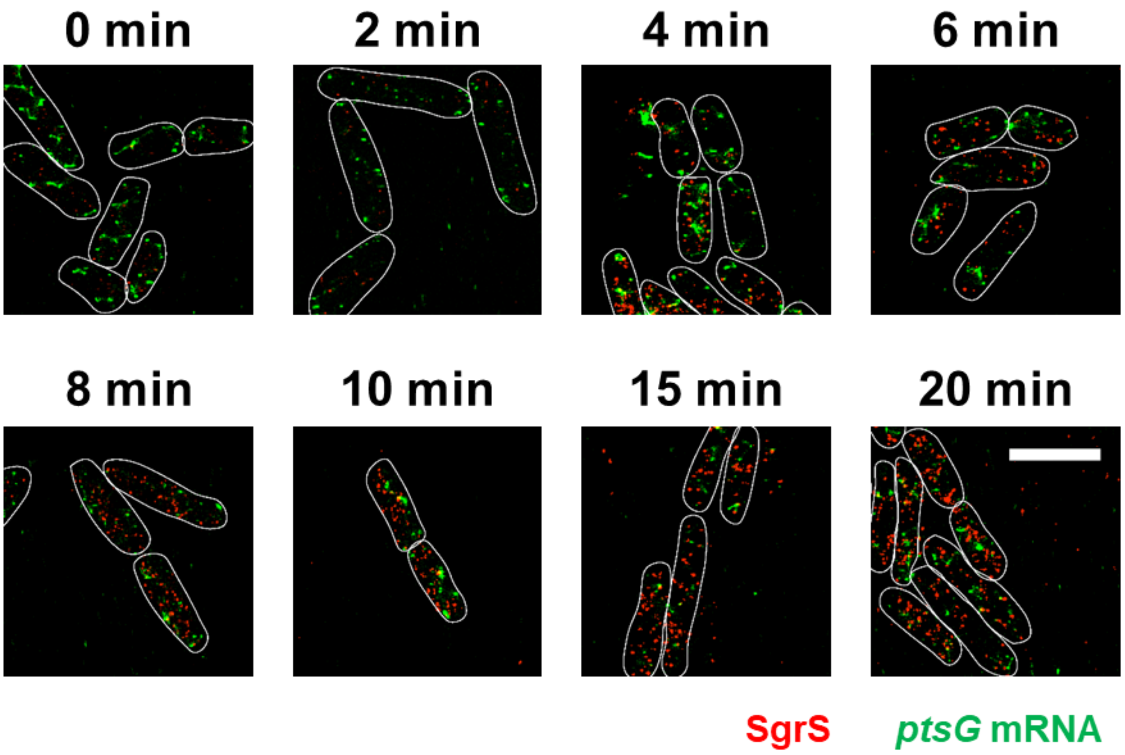

Supplementary Figure 22. 3D super-resolution images of SgrS (red) and *ptsG* mRNA (green) in the SgrS G215A RNase E mutant strain projected on 2D planes. The panels show the multi-color images of SgrS G215A RNase E mutant cells before (0 min) and 2, 4, 6, 8, 10, 15, 20 min after αMG (non-metabolizable sugar analog) induction. Each experiment was performed independently 2 times. White lines denote cell boundaries. Scale bar is 2 μm and applies to all the panels.

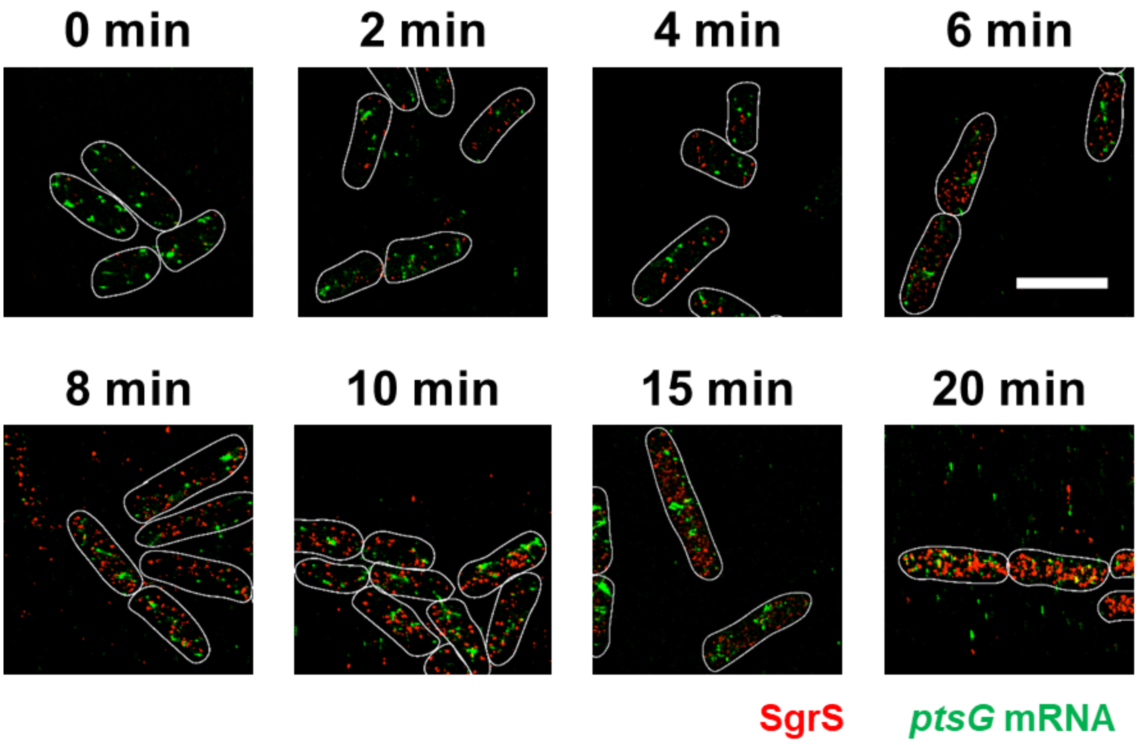

Supplementary Figure 23. 3D super-resolution images of SgrS (red) and *ptsG* mRNA (green) in the SgrS U224A RNase E mutant strain projected on 2D planes. The panels show the multi-color images of SgrS U224A RNase E mutant cells before (0 min) and 2, 4, 6, 8, 10, 15, 20 min after αMG (non-metabolizable sugar analog) induction. Each experiment was performed independently 2 times. White lines denote cell boundaries. Scale bar is 2 μm and applies to all the panels.

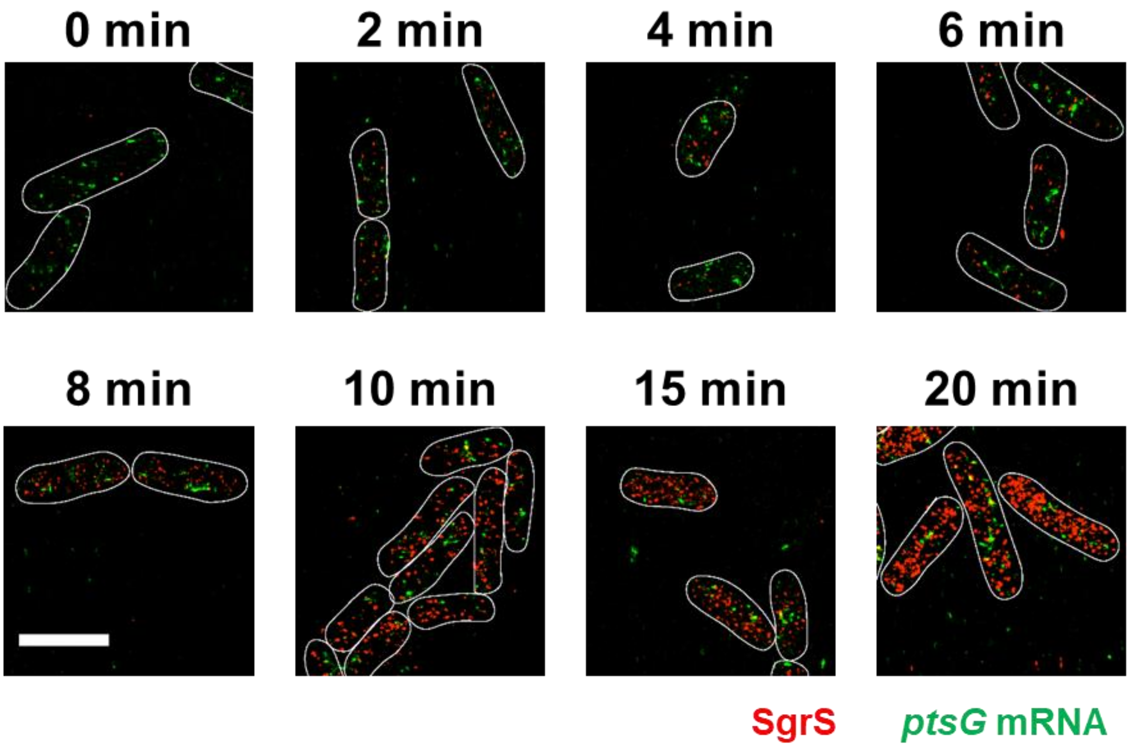

Supplementary Figure 24. 3D super-resolution images of SgrS (red) and *ptsG* mRNA (green) in the SgrS U224G RNase E mutant strain projected on 2D planes. The panels show the multi-color images of SgrS U224G RNase E mutant cells before (0 min) and 2, 4, 6, 8, 10, 15, 20 min after αMG (non-metabolizable sugar analog) induction. Each experiment was performed independently 2 times. White lines denote cell boundaries. Scale bar is 2 μm and applies to all the panels.

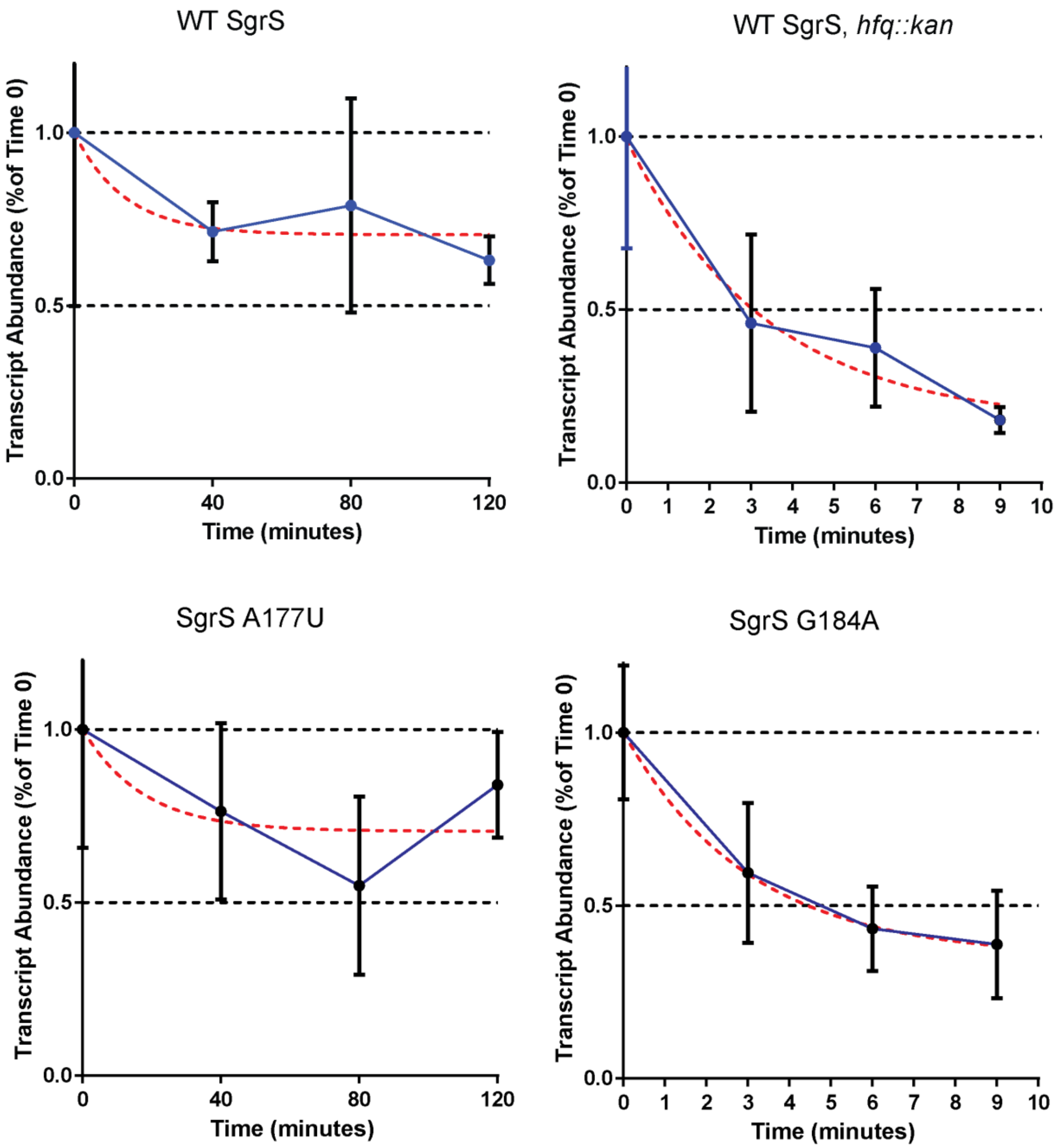

Supplementary Figure 25. Stability of SgrS transcripts in different strains, (a) wild-type SgrS, **(b)** Δ*hfq* wild-type strain, **(c)** SgrS A177U strain, **(d)** SgrS G184A strain. Rifampicin was added to the SgrS induced culture to inhibit the synthesis of all cellular transcripts (including SgrS and all its targets). To measure target independent half-life of SgrS, the starting time point was set 5 minutes after the addition of rifampicin. The cells were harvested for total RNA

extraction at the indicated time points, and mRNA levels were measured with RT-PCR. The relative transcript concentration was determined using ΔΔCq method normalized to 16S ribosome gene (*rrsA*) transcripts and was expressed as fractions of abundance in time point 0 samples. The transcript turnover rates were calculated based on the non-linear fit one phase exponential decay curves using GraphPad software (red dotted lines). Data was obtained from n=3 biological replicates and presented as mean values +/- SD. Source data are provided as a Source Data file.

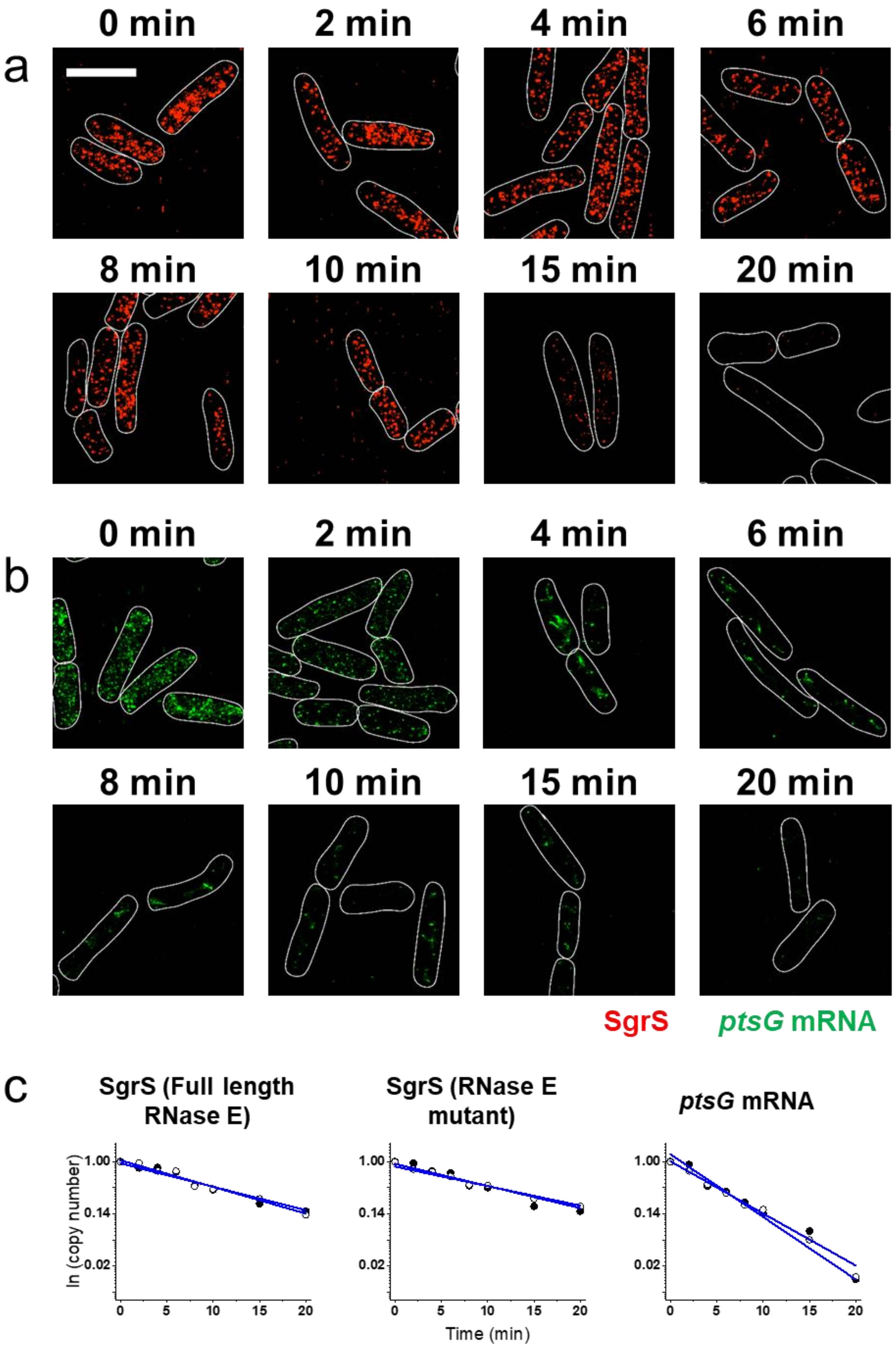
0 min
2 min
4 min
6 min
a
8 min
10 min
15 min
20 min
0 min
2 min
4 min
6 min
b
8 min
10 min
15 min
20 min
SgrS
ptsG mRNA
c
SgrS (Full length RNase E)
SgrS (RNase E mutant)
ptsG mRNA
ln (copy number)
1.00
0.14
0.02
0
5
10
15
20
Time (min)

251

Supplementary Figure 26. RNA lifetime measurements for the wild-type strain. (a) SgrS degradation in the wild-type strain. Each experiment was performed independently 2 times. **(b)** *ptsG* mRNA degradation in the wild-type strain. Each experiment was performed independently 2 times. **(c)** Calculation of RNA lifetime. Filled and open circles are two independent measurements with mean value from ~80 cells in each case. The copy numbers have been normalized to time t=0 in each case. The degradation rates calculated from the lifetimes are shown in Fig. 6a, Supplementary Fig. 37 and Supplementary Table 3. Scale bar is 2 μm and applies to all the panels. Source data are provided as a Source Data file.

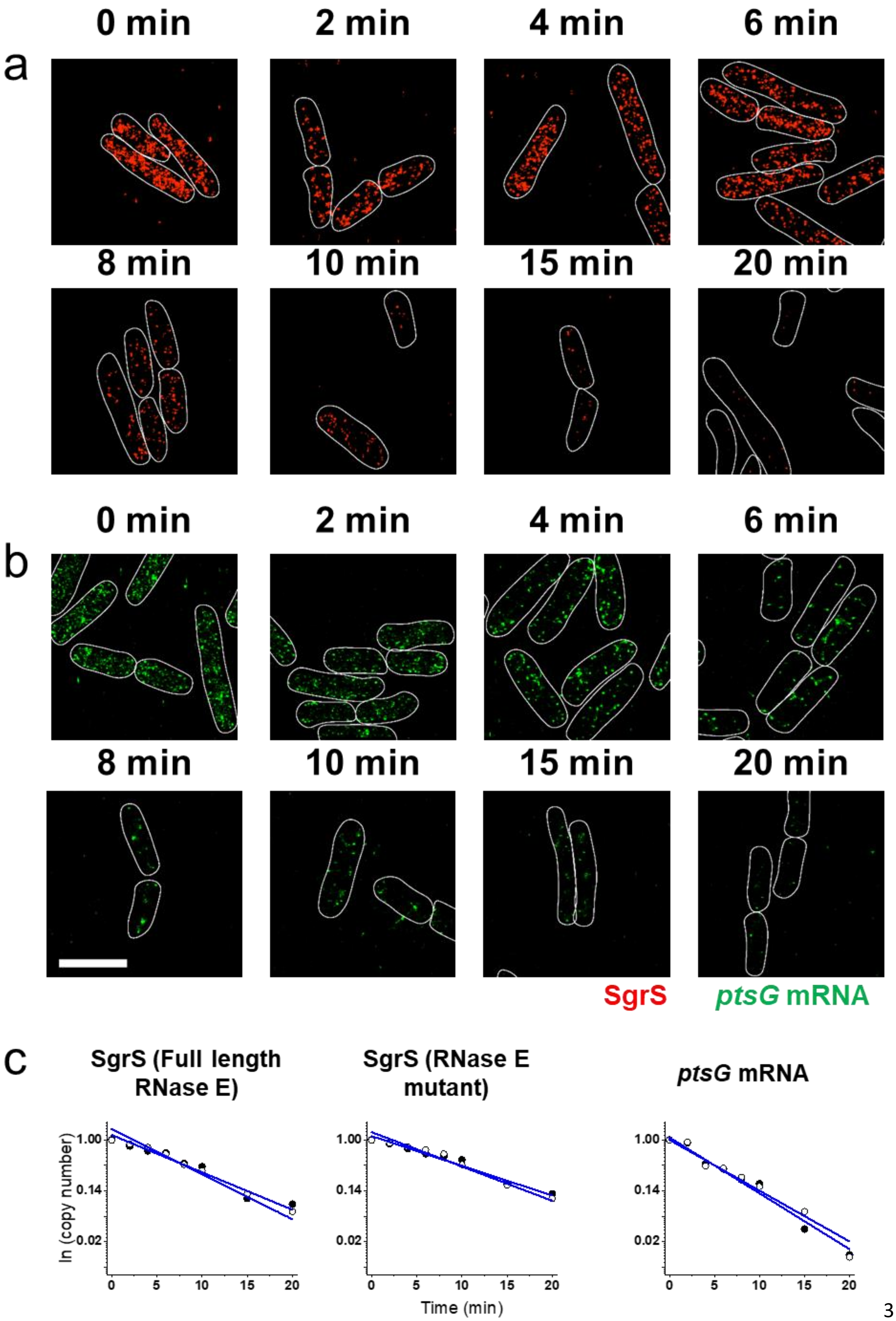
0 min
2 min
4 min
6 min
a
8 min
10 min
15 min
20 min
0 min
2 min
4 min
6 min
b
8 min
10 min
15 min
20 min
SgrS
ptsG mRNA
c
SgrS (Full length RNase E)
SgrS (RNase E mutant)
ptsG mRNA
ln (copy number)
1.00
0.14
0.02
0
5
10
15
20
Time (min)

Supplementary Figure 27. RNA lifetime measurements for the A177U strain. (a) SgrS degradation in the A177U strain. Each experiment was performed independently 2 times. **(b)** *ptsG* mRNA degradation in the A177U strain. Each experiment was performed independently 2 times. **(c)** Calculation of RNA lifetime. Filled and open circles are two independent measurements with mean value from ~80 cells in each case. The copy numbers have been normalized to time t=0 in each case. The degradation rates calculated from the lifetimes are shown in Fig. 6a, Supplementary Fig. 37 and Supplementary Table 3. Scale bar is 2 μm and applies to all the panels. Source data are provided as a Source Data file.

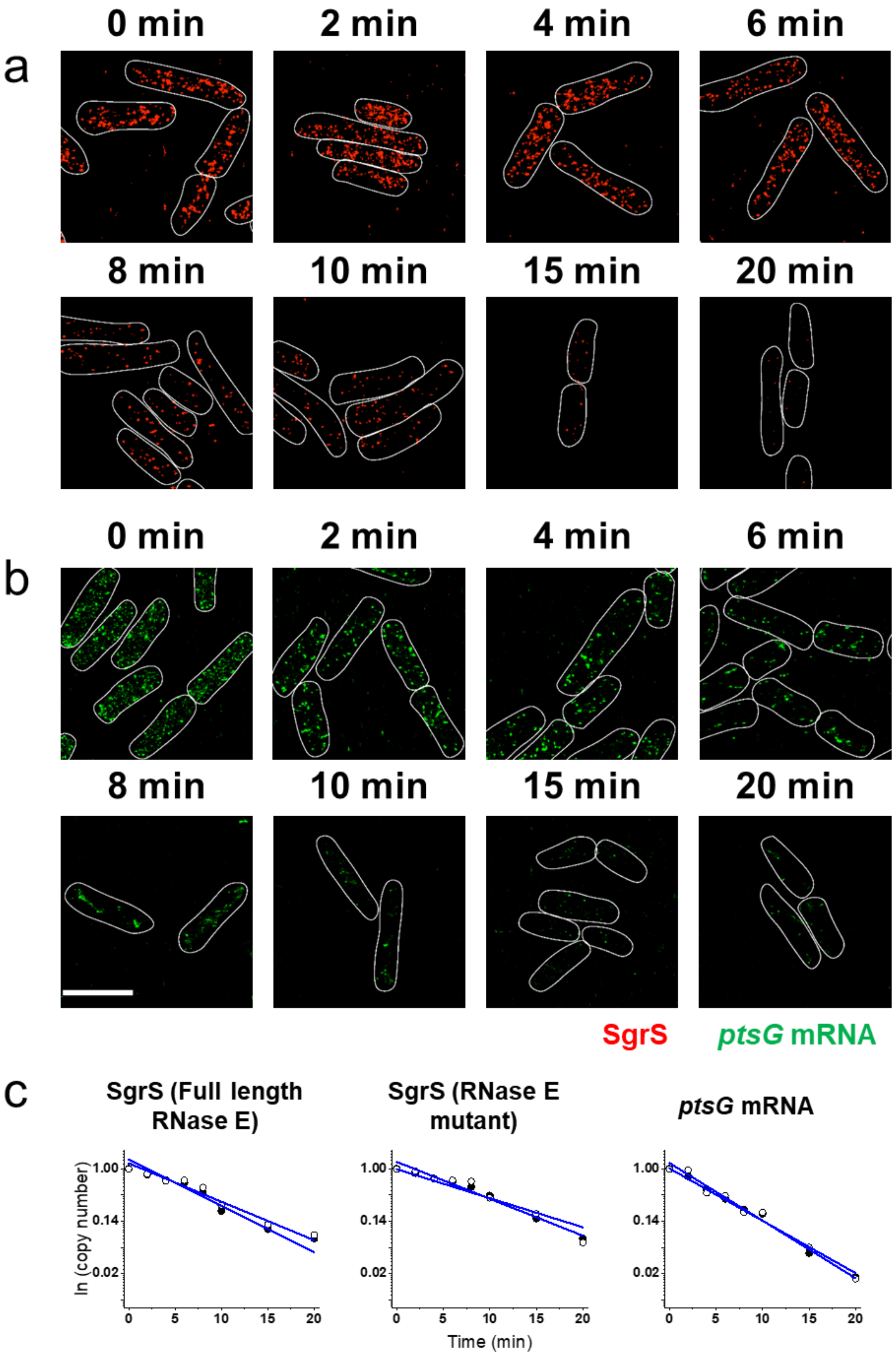

a
0 min
2 min
4 min
6 min
8 min
10 min
15 min
20 min
b
0 min
2 min
4 min
6 min
8 min
10 min
15 min
20 min
SgrS
ptsG mRNA
c
SgrS (Full length RNase E)
SgrS (RNase E mutant)
ptsG mRNA
ln (copy number)
1.00
0.14
0.02
0
5
10
15
20
Time (min)

Supplementary Figure 28. RNA lifetime measurements for the G178A strain. (a) SgrS degradation in the G178A strain. Each experiment was performed independently 2 times. **(b)** *ptsG* mRNA degradation in the G178A strain. Each experiment was performed independently 2 times. **(c)** Calculation of RNA lifetime. Filled and open circles are two independent measurements with mean value from ~80 cells in each case. The copy numbers have been normalized to time t=0 in each case. The degradation rates calculated from the lifetimes are shown in Fig. 6a, Supplementary Fig. 37 and Supplementary Table 3. Scale bar is 2 μm and applies to all the panels. Source data are provided as a Source Data file.

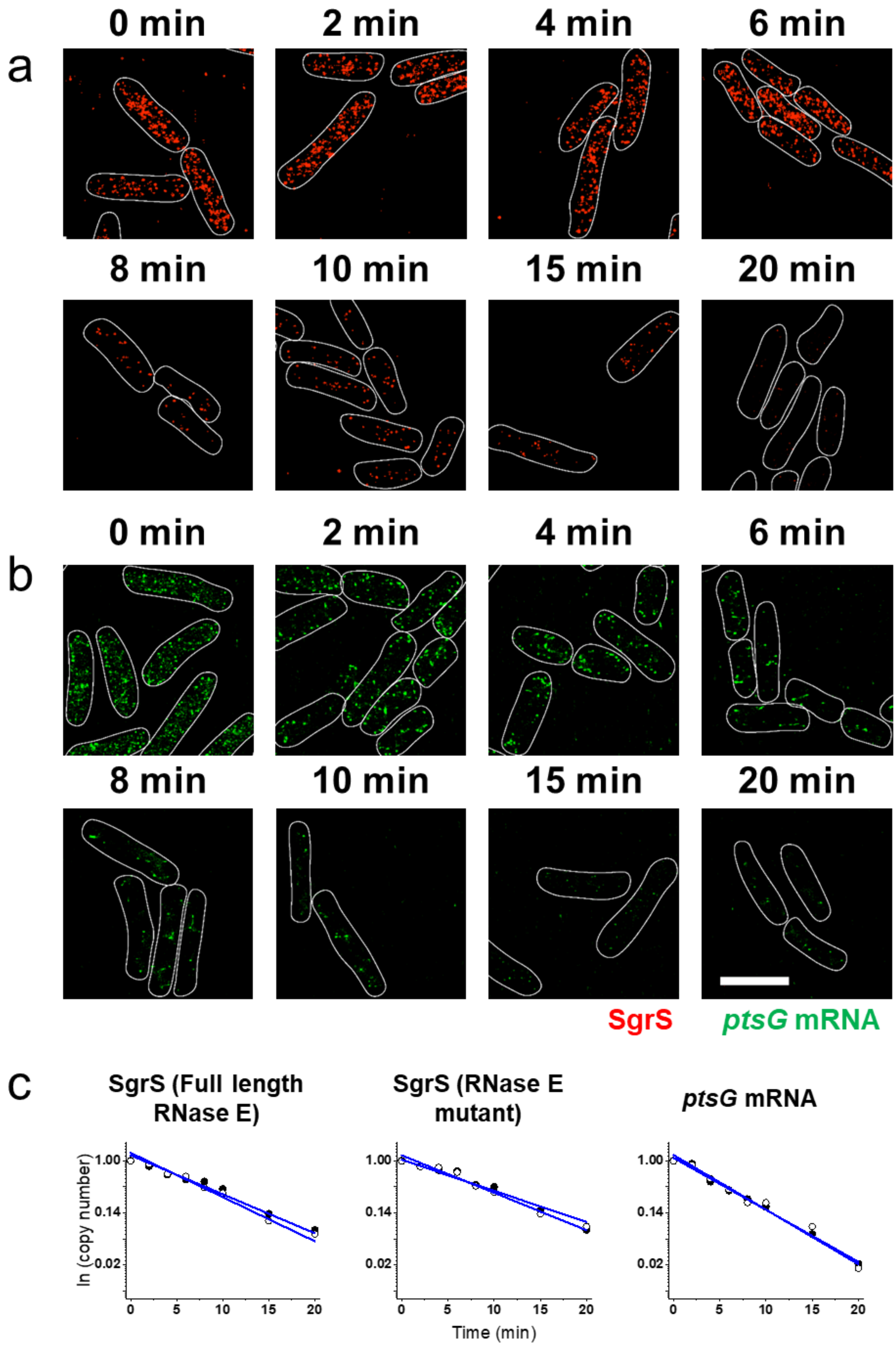
a
0 min
2 min
4 min
6 min
8 min
10 min
15 min
20 min
b
0 min
2 min
4 min
6 min
8 min
10 min
15 min
20 min
SgrS
ptsG mRNA
c
SgrS (Full length RNase E)
SgrS (RNase E mutant)
ptsG mRNA
ln (copy number)
1.00
0.14
0.02
0
5
10
15
20
Time (min)

Supplementary Figure 29. RNA lifetime measurements for the G178U strain. (a) SgrS degradation in the G178U strain. Each experiment was performed independently 2 times. **(b)** *ptsG* mRNA degradation in the G178U strain. Each experiment was performed independently 2 times. **(c)** Calculation of RNA lifetime. Filled and open circles are two independent measurements with mean value from ~80 cells in each case. The copy numbers have been normalized to time t=0 in each case. The degradation rates calculated from the lifetimes are shown in Fig. 6a, Supplementary Fig. 37 and Supplementary Table 3. Scale bar is 2 μm and applies to all the panels. Source data are provided as a Source Data file.

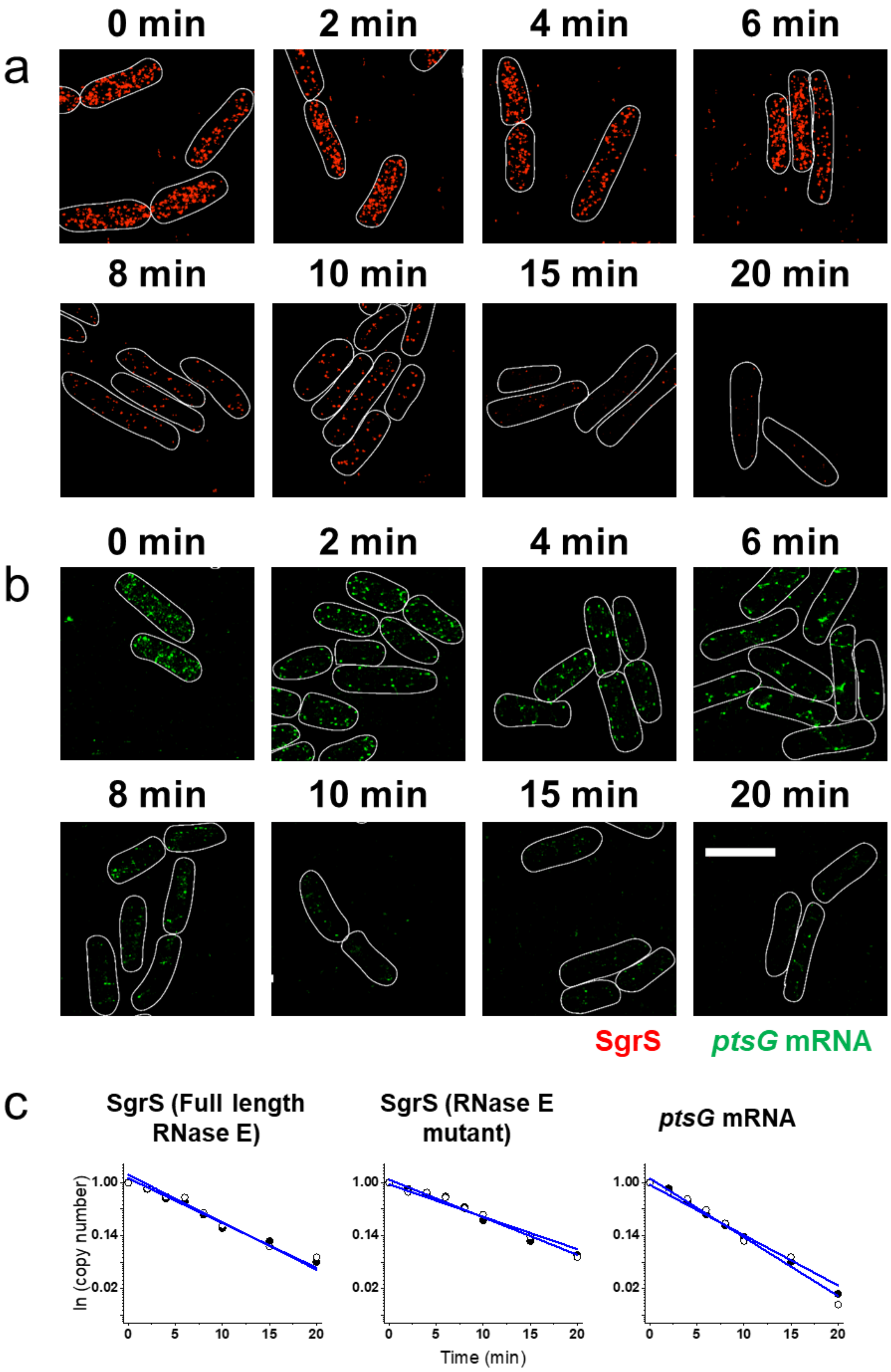
a
0 min
2 min
4 min
6 min
8 min
10 min
15 min
20 min
b
0 min
2 min
4 min
6 min
8 min
10 min
15 min
20 min
SgrS
ptsG mRNA
c
SgrS (Full length RNase E)
SgrS (RNase E mutant)
ptsG mRNA
ln (copy number)
1.00
0.14
0.02
0
5
10
15
20
Time (min)

Supplementary Figure 30. RNA lifetime measurements for the U181A strain. (a) SgrS degradation in the U181A strain. Each experiment was performed independently 2 times. **(b)** *ptsG* mRNA degradation in the U181A strain. Each experiment was performed independently 2 times. **(c)** Calculation of RNA lifetime. Filled and open circles are two independent measurements with mean value from ~80 cells in each case. The copy numbers have been normalized to time t=0 in each case. The degradation rates calculated from the lifetimes are shown in Fig. 6a, Supplementary Fig. 37 and Supplementary Table 3. Scale bar is 2 μm and applies to all the panels. Source data are provided as a Source Data file.

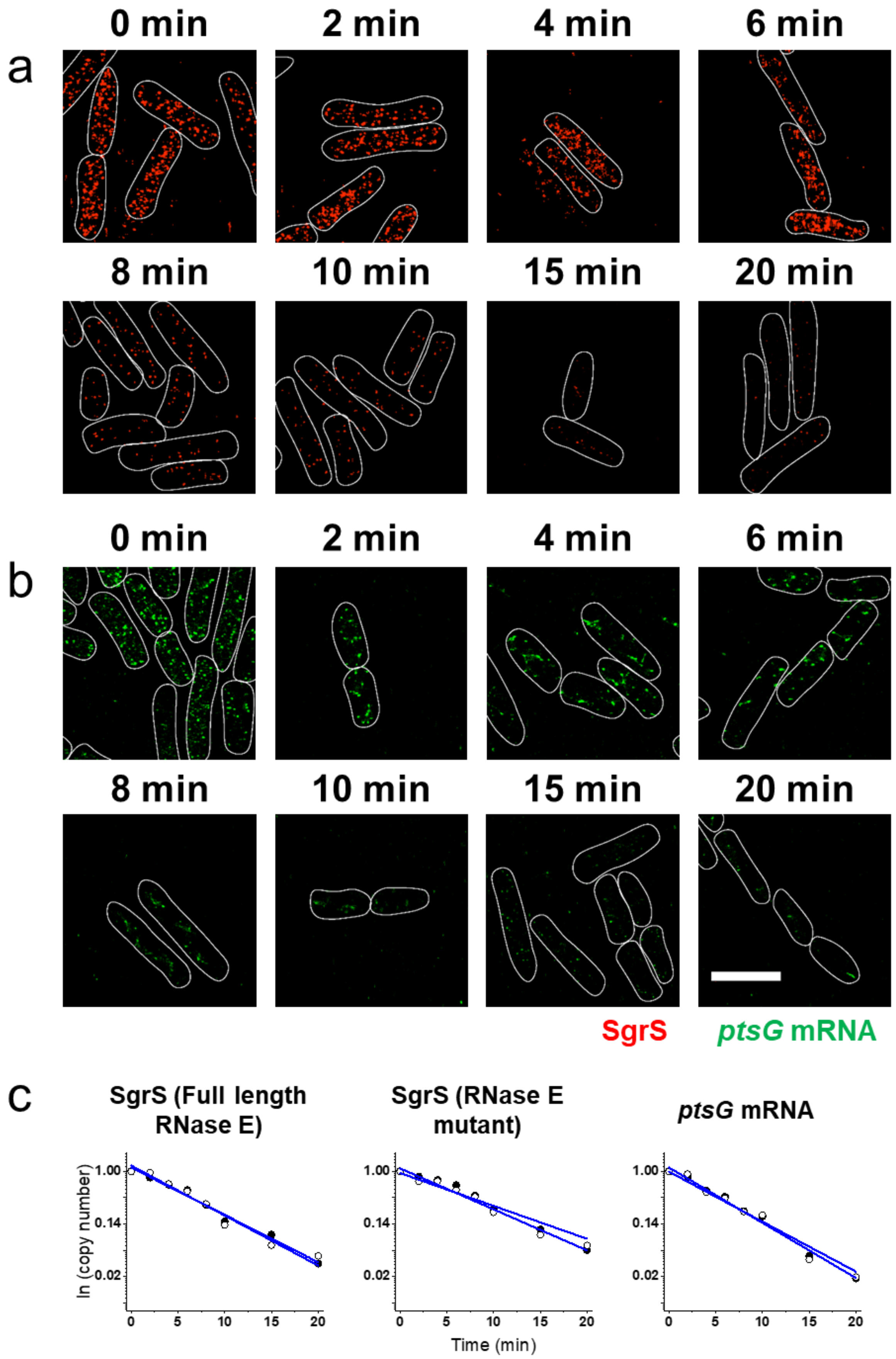
a
0 min
2 min
4 min
6 min
8 min
10 min
15 min
20 min
b
0 min
2 min
4 min
6 min
8 min
10 min
15 min
20 min
SgrS
ptsG mRNA
c
SgrS (Full length RNase E)
SgrS (RNase E mutant)
ptsG mRNA
ln (copy number)
1.00
0.14
0.02
0
5
10
15
20
Time (min)

Supplementary Figure 31. RNA lifetime measurements for the U182A strain. (a) SgrS degradation in the U182A strain. Each experiment was performed independently 2 times. **(b)** *ptsG* mRNA degradation in the U182A strain. Each experiment was performed independently 2 times. **(c)** Calculation of RNA lifetime. Filled and open circles are two independent measurements with mean value from ~80 cells in each case. The copy numbers have been normalized to time t=0 in each case. The degradation rates calculated from the lifetimes are shown in Fig. 6a, Supplementary Fig. 37 and Supplementary Table 3. Scale bar is 2 μm and applies to all the panels. Source data are provided as a Source Data file.

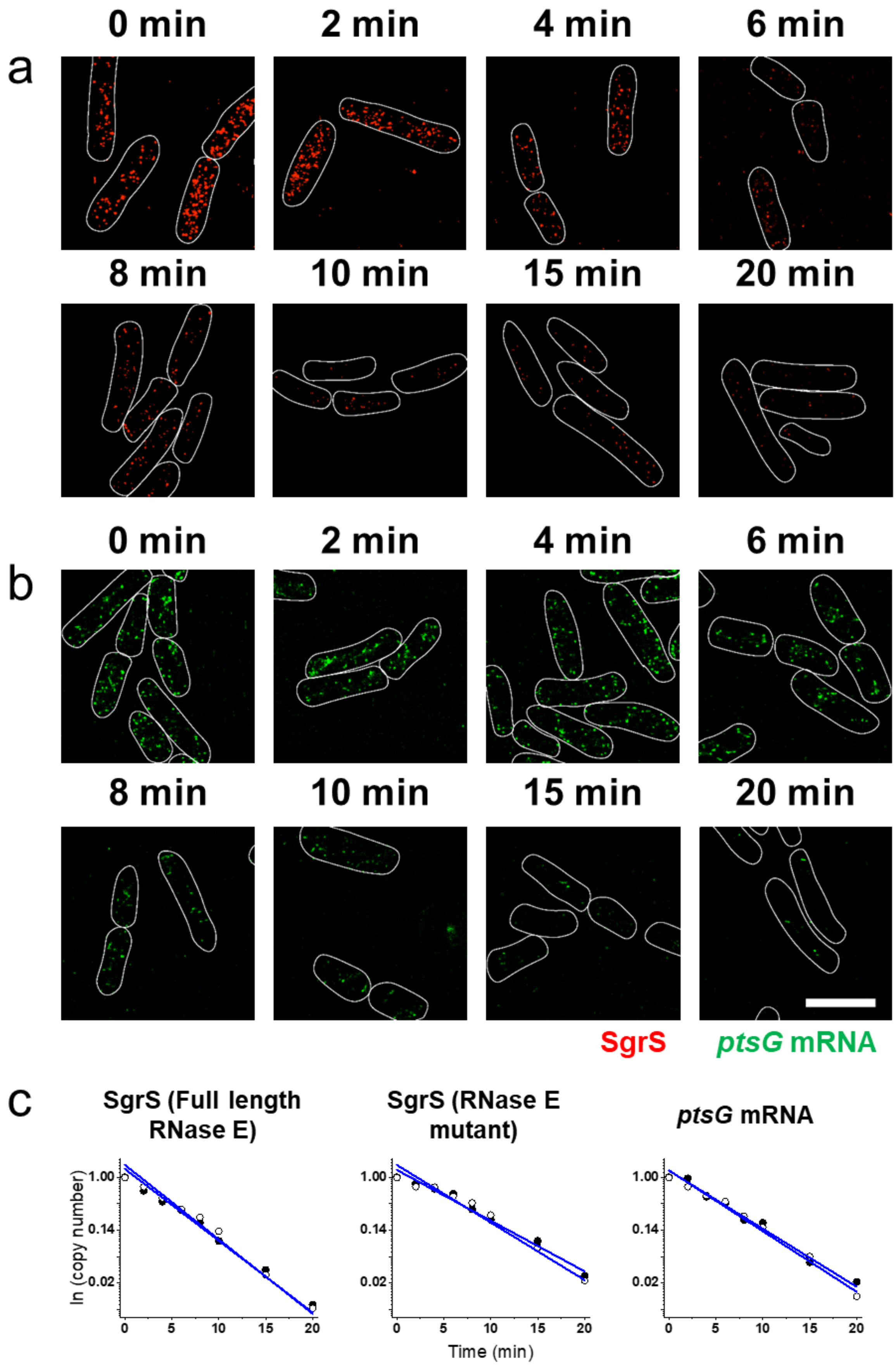
0 min
2 min
4 min
6 min
a
8 min
10 min
15 min
20 min
0 min
2 min
4 min
6 min
b
8 min
10 min
15 min
20 min
SgrS
ptsG mRNA
c
SgrS (Full length RNase E)
SgrS (RNase E mutant)
ptsG mRNA
ln (copy number)
1.00
0.14
0.02
0
5
10
15
20
Time (min)

Supplementary Figure 32. RNA lifetime measurements for the G184A strain. (a) SgrS degradation in the G184A strain. Each experiment was performed independently 2 times. **(b)** *ptsG* mRNA degradation in the G184A strain. Each experiment was performed independently 2 times. **(c)** Calculation of RNA lifetime. Filled and open circles are two independent measurements with mean value from ~80 cells in each case. The copy numbers have been normalized to time t=0 in each case. The degradation rates calculated from the lifetimes are shown in Fig. 6a, Supplementary Fig. 37 and Supplementary Table 3. Scale bar is 2 μm and applies to all the panels. Source data are provided as a Source Data file.

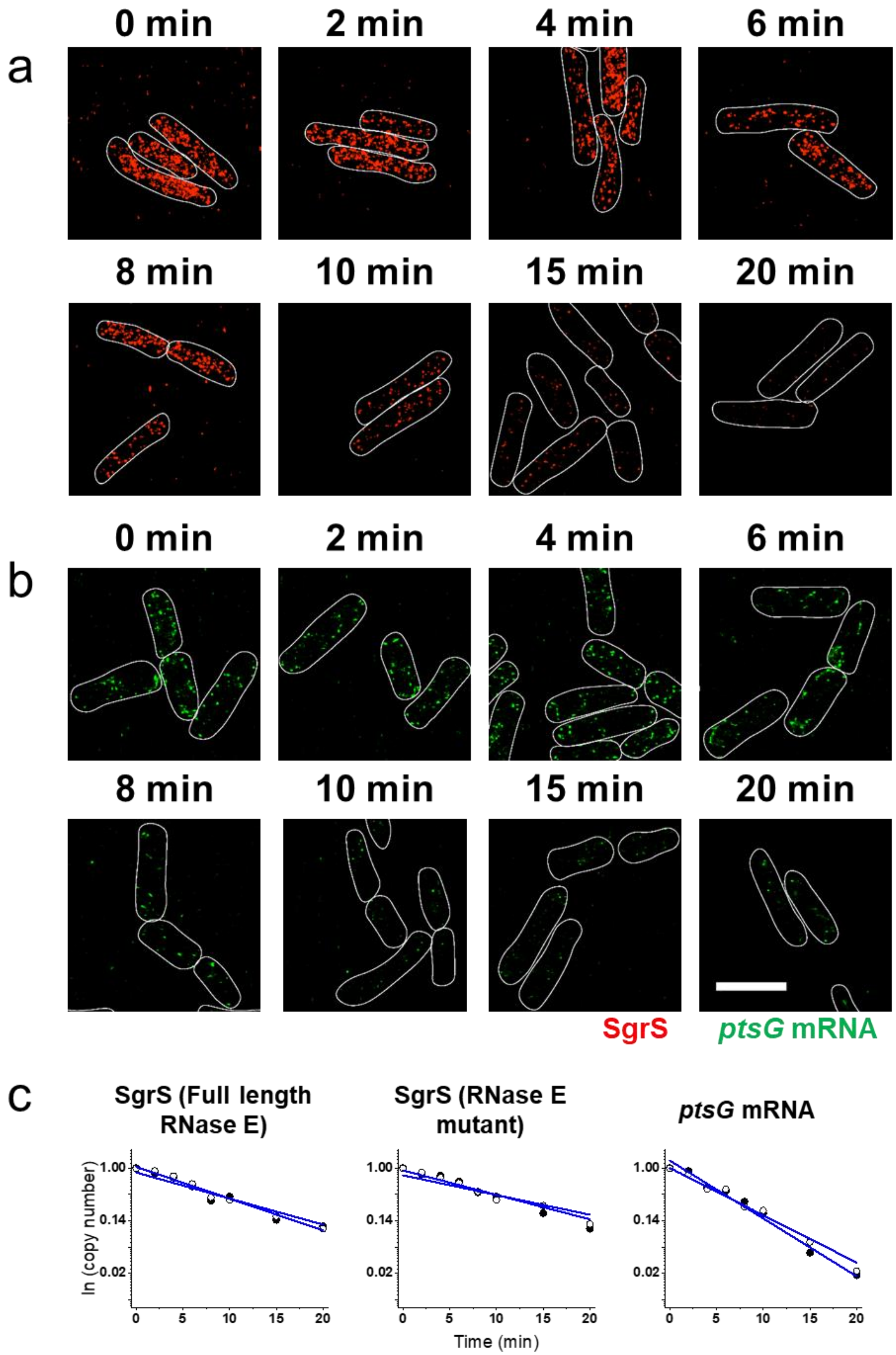

0 min
2 min
4 min
6 min
a
8 min
10 min
15 min
20 min
0 min
2 min
4 min
6 min
b
8 min
10 min
15 min
20 min
SgrS
ptsG mRNA
c
SgrS (Full length RNase E)
SgrS (RNase E mutant)
ptsG mRNA
ln (copy number)
1.00
0.14
0.02
0
5
10
15
20
Time (min)

Supplementary Figure 33. RNA lifetime measurements for the G184A-C195U strain. (a) SgrS degradation in the G184A-C195U strain. Each experiment was performed independently 2 times. **(b)** *ptsG* mRNA degradation in the G184A-C195U strain. Each experiment was performed independently 2 times. **(c)** Calculation of RNA lifetime. Filled and open circles are two independent measurements with mean value from ~80 cells in each case. The copy numbers have been normalized to time t=0 in each case. The degradation rates calculated from the lifetimes are shown in Fig. 6a, Supplementary Fig. 37 and Supplementary Table 3. Scale bar is 2 μm and applies to all the panels. Source data are provided as a Source Data file.

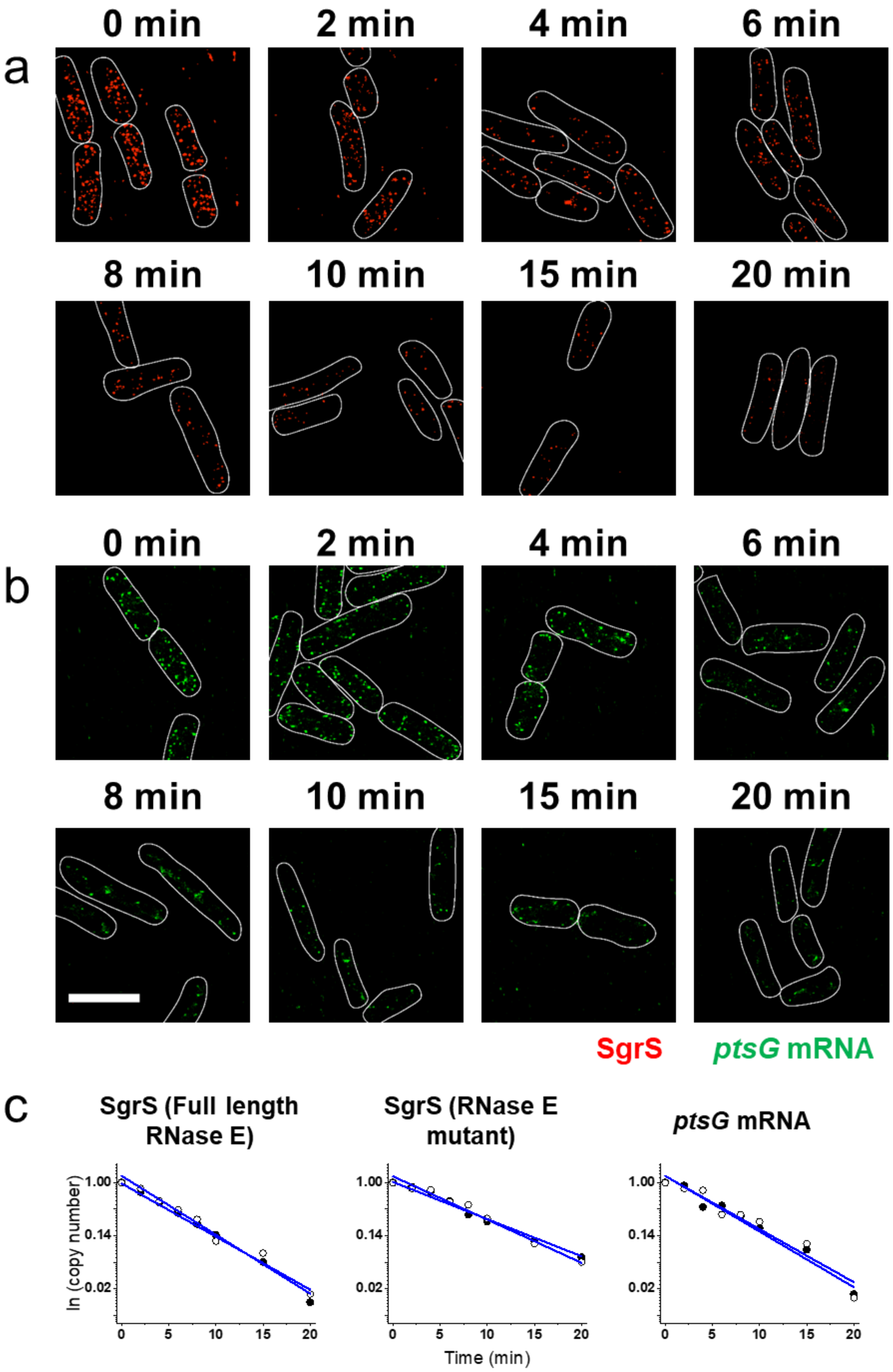
a
0 min
2 min
4 min
6 min
8 min
10 min
15 min
20 min
b
0 min
2 min
4 min
6 min
8 min
10 min
15 min
20 min
SgrS
ptsG mRNA
c
SgrS (Full length RNase E)
SgrS (RNase E mutant)
ptsG mRNA
ln (copy number)
1.00
0.14
0.02
0
5
10
15
20
Time (min)

Supplementary Figure 34. RNA lifetime measurements for the G215A strain. (a) SgrS degradation in the G215A strain. Each experiment was performed independently 2 times. **(b)** *ptsG* mRNA degradation in the G215A strain. Each experiment was performed independently 2 times. **(c)** Calculation of RNA lifetime. Filled and open circles are two independent measurements with mean value from ~80 cells in each case. The copy numbers have been normalized to time t=0 in each case. The degradation rates calculated from the lifetimes are shown in Fig. 6a, Supplementary Fig. 37 and Supplementary Table 3. Scale bar is 2 μm and applies to all the panels. Source data are provided as a Source Data file.

350

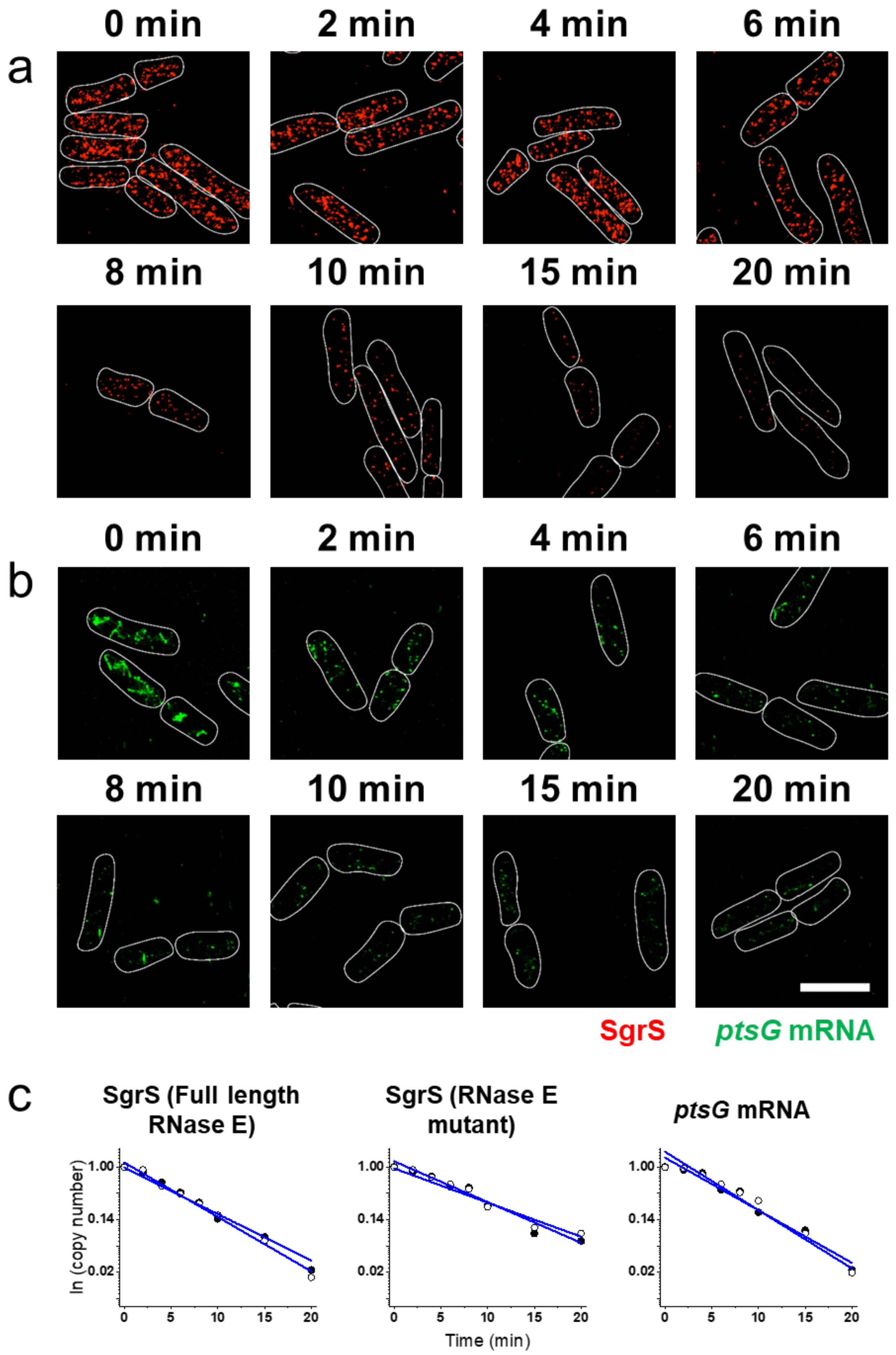

0 min
2 min
4 min
6 min
a
8 min
10 min
15 min
20 min
0 min
2 min
4 min
6 min
b
8 min
10 min
15 min
20 min
SgrS
ptsG mRNA
c
SgrS (Full length RNase E)
SgrS (RNase E mutant)
ptsG mRNA
ln (copy number)
1.00
0.14
0.02
0
5
10
15
20
Time (min)

Supplementary Figure 35. RNA lifetime measurements for the U224A strain. (a) SgrS degradation in the U224A strain. Each experiment was performed independently 2 times. **(b)** *ptsG* mRNA degradation in the U224A strain. Each experiment was performed independently 2 times. **(c)** Calculation of RNA lifetime. Filled and open circles are two independent measurements with mean value from ~80 cells in each case. The copy numbers have been normalized to time t=0 in each case. The degradation rates calculated from the lifetimes are shown in Fig. 6a, Supplementary Fig. 37 and Supplementary Table 3. Scale bar is 2 μm and applies to all the panels. Source data are provided as a Source Data file.

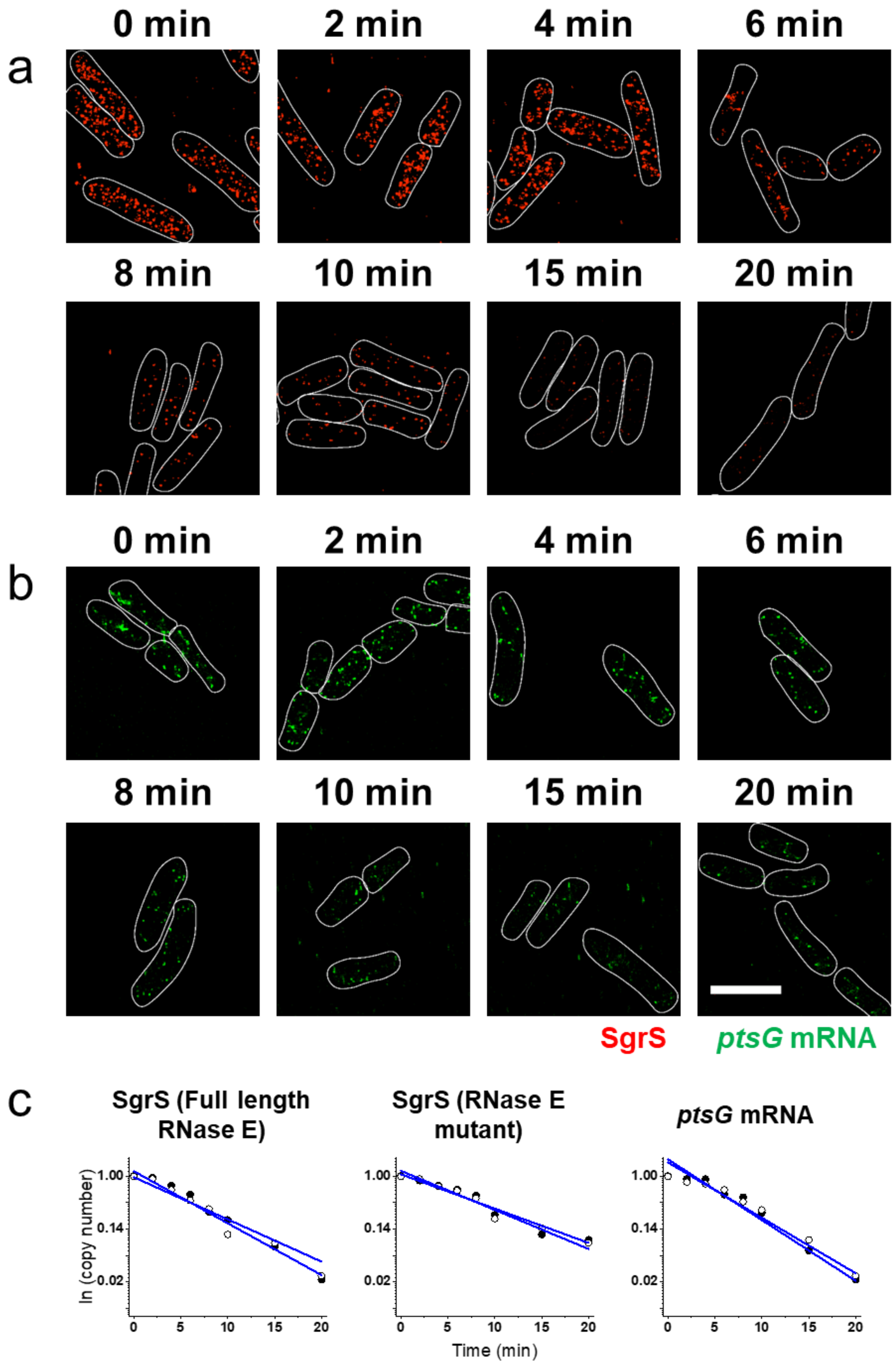
a
0 min
2 min
4 min
6 min
8 min
10 min
15 min
20 min
b
0 min
2 min
4 min
6 min
8 min
10 min
15 min
20 min
SgrS
ptsG mRNA
c
SgrS (Full length RNase E)
SgrS (RNase E mutant)
ptsG mRNA
ln (copy number)
1.00
0.14
0.02
0
5
10
15
20
Time (min)

Supplementary Figure 36. RNA lifetime measurements for the U224G strain. (a) SgrS degradation in the U224G strain. Each experiment was performed independently 2 times. **(b)** *ptsG* mRNA degradation in the U224G strain. Each experiment was performed independently 2 times. **(c)** Calculation of RNA lifetime. Filled and open circles are two independent measurements with mean value from ~80 cells in each case. The copy numbers have been normalized to time t=0 in each case. The degradation rates calculated from the lifetimes are shown in Fig. 6a, Supplementary Fig. 37 and Supplementary Table 3. Scale bar is 2 μm and applies to all the panels. Source data are provided as a Source Data file.

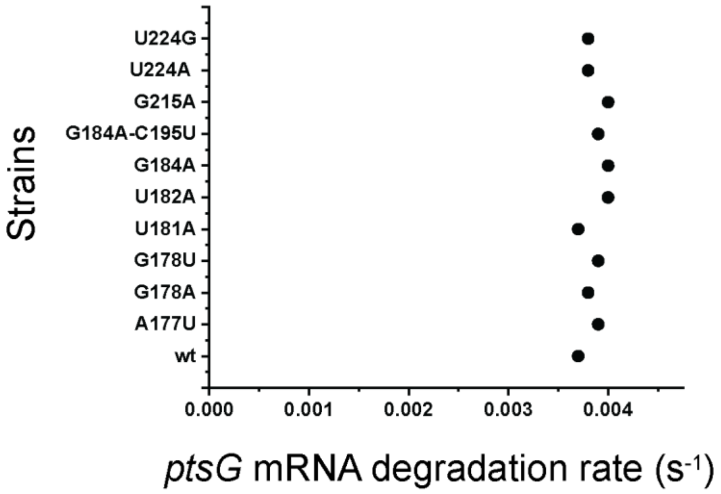

Supplementary Figure 37. Degradation rates of *ptsG* mRNA in wild-type and the mutant strains. Degradation rates of *ptsG* mRNA in wild-type and the strains A177U, G178A, G178U, U181A, U182A, G184A, G184A-C195U, G215A, U224A and U224G. The plot shows mean values obtained from two experimental replicates. Source data are provided as a Source Data file.

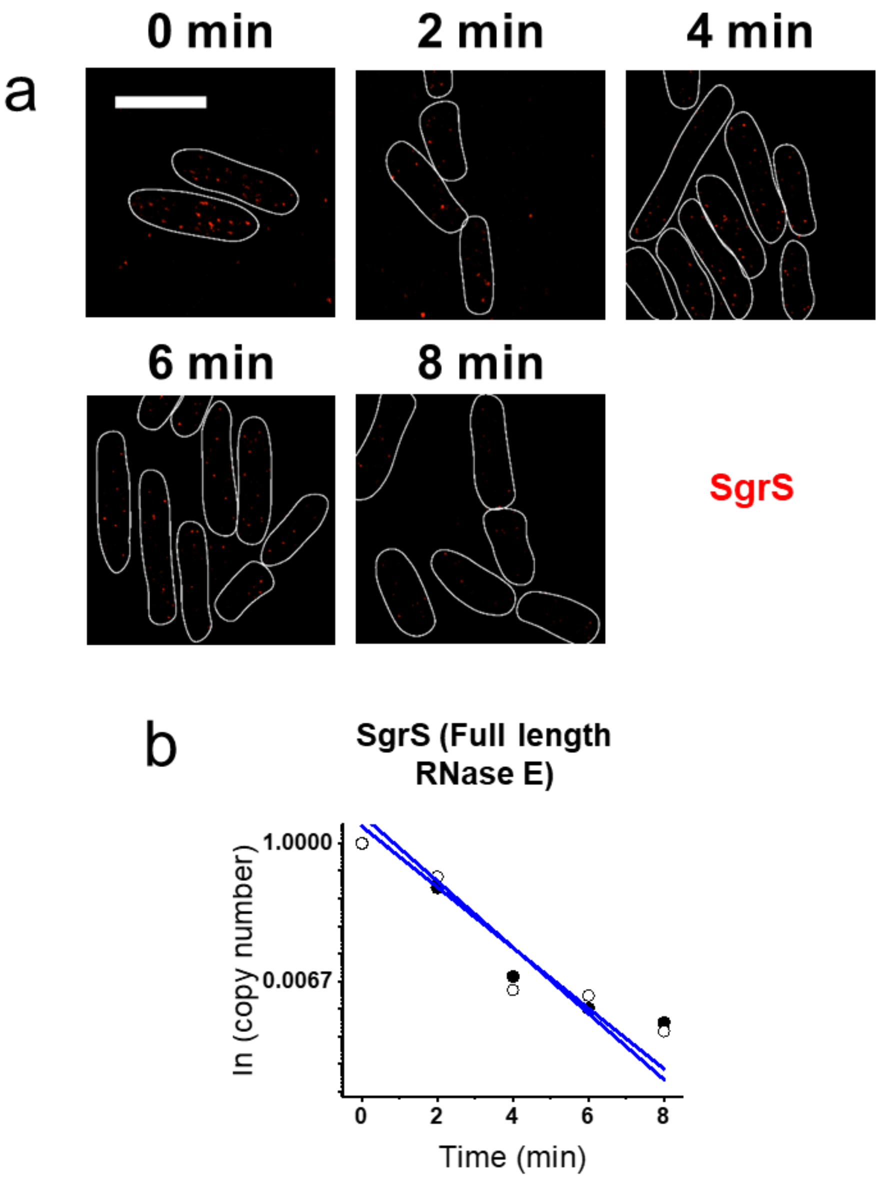

Supplementary Figure 38. RNA lifetime measurements for the Δ*hfq* wild-type strain. (a) SgrS degradation in the Δ*hfq* wild-type strain. Each experiment was performed independently 2 times. Scale bar is 2 μm and applies to all the panels. **(b)** Calculation of RNA lifetime. Filled and open circles are two independent measurements with mean value from ~70 cells in each case. The copy numbers have been normalized to time t=0 in each case. The degradation rates calculated from the lifetimes are shown in Fig 6a. Source data are provided as a Source Data file.

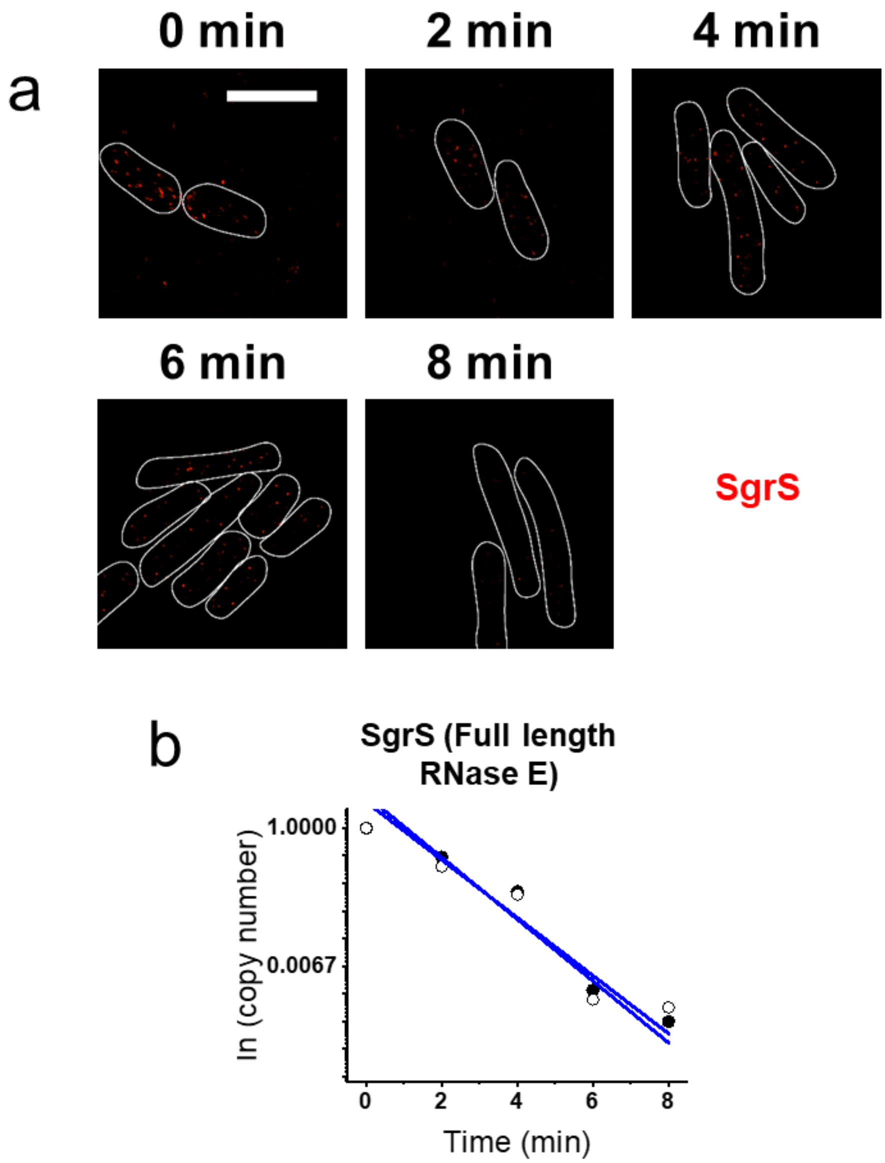

Supplementary Figure 39. RNA lifetime measurements for the Δ*hfq* A177U strain. (a) SgrS degradation in the Δ*hfq* A177U strain. Each experiment was performed independently 2 times. Scale bar is 2 μm and applies to all the panels. **(b)** Calculation of RNA lifetime. Filled and open circles are two independent measurements with mean value from ~70 cells in each case. The copy numbers have been normalized to time t=0 in each case. The degradation rates calculated from the lifetimes are shown in Fig 6a. Source data are provided as a Source Data file.

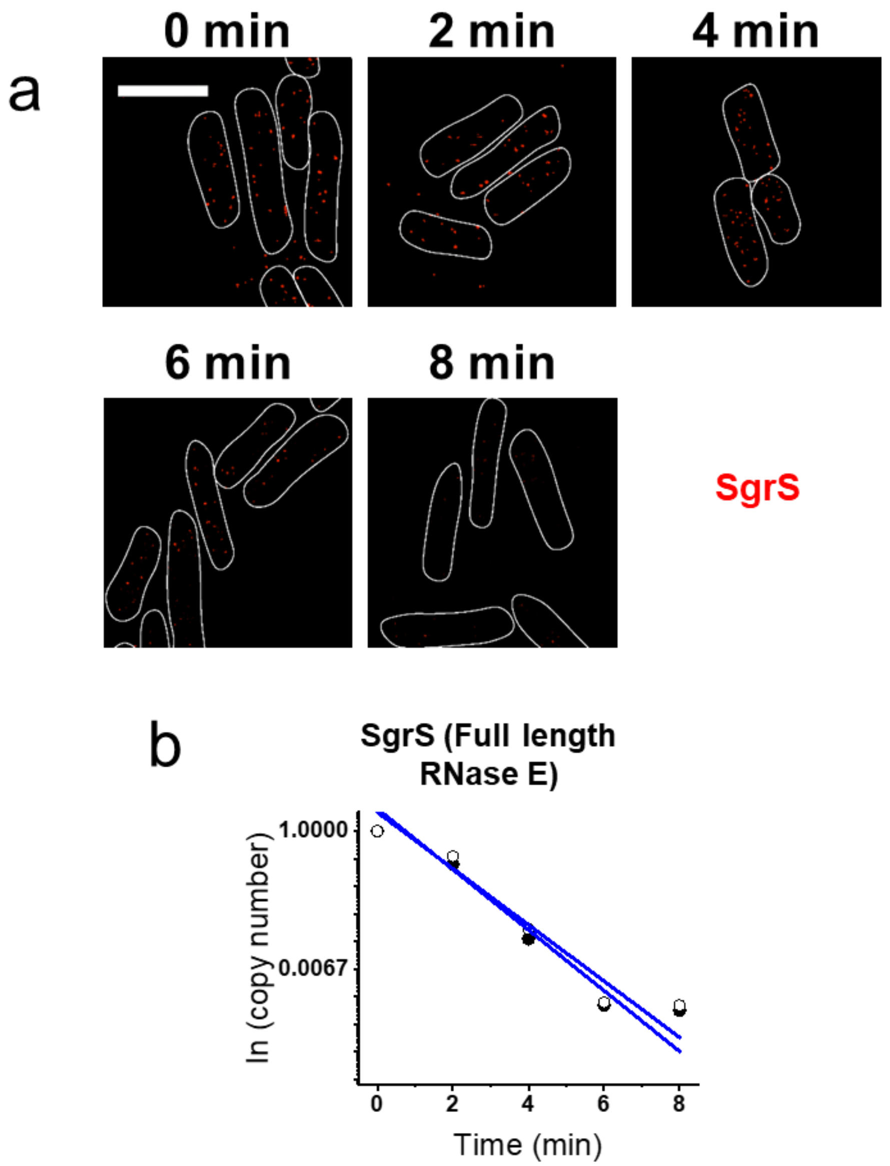

Supplementary Figure 40. RNA lifetime measurements for the Δ*hfq* G184A strain. (a) SgrS degradation in the Δ*hfq* G184A strain. Each experiment was performed independently 2 times. Scale bar is 2 μm and applies to all the panels. **(b)** Calculation of RNA lifetime. Filled and open circles are two independent measurements with mean value from ~70 cells in each case. The copy numbers have been normalized to time t=0 in each case. The degradation rates calculated from the lifetimes are shown in Fig 6a. Source data are provided as a Source Data file.

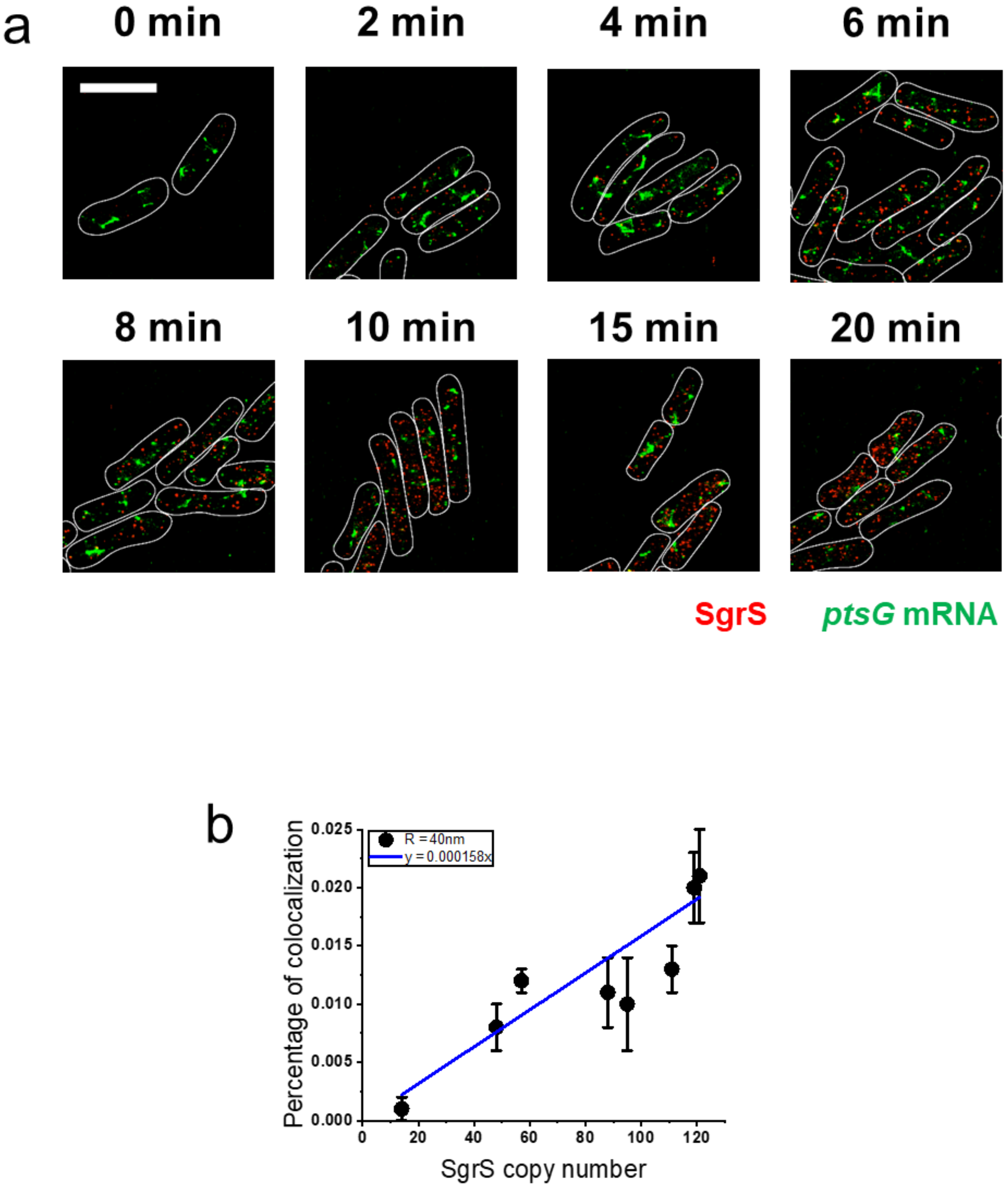

Supplementary Figure 41. Colocalization analysis for base-pairing mutant strain. (a) 3D super-resolution images of SgrS (red) and *ptsG* mRNA (green) in the base-pairing mutant strain projected on 2D planes. The panels show the multi-color images of cells before (0 min) and 2, 4,

6, 8, 10, 15, 20 min after αMG (non-metabolizable sugar analog) induction. Each experiment was performed independently 2 times. White lines denote cell boundaries. Scale bar is 2 μm and applies to all the panels. (b) The percentage of colocalization in the base-pairing mutant strain with R=40 nm as a function of mean SgrS copy number. The plot is fit with a linear function for correction of colocalization by chance. Data are presented as mean values +/-SD from n=3, 5, 4, 3, 6, 5, 4, 3 images for 0, 2, 4, 6, 8, 10, 15, 20 min after αMG induction. Source data are provided as a Source Data file.

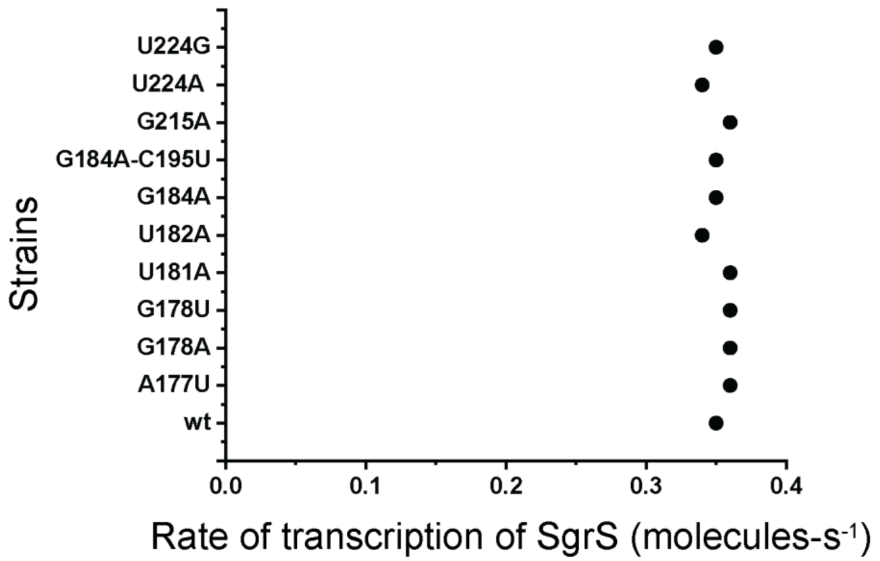

Supplementary Figure 42. Rates of transcription of SgrS for wild-type and the mutant strains. Rates of transcription of SgrS for wild-type and the strains A177U, G178A, G178U, U181A, U182A, G184A, G184A-C195U, G215A, U224A and U224G calculated from the time dependent modeling curves of the SgrS, *ptsG* mRNA and SgrS-*ptsG* mRNA complexes. These were determined simultaneously in the wild-type and the RNase E mutants. The plot shows mean values from the independent fitting of two replicates. Source data are provided as a Source Data file.

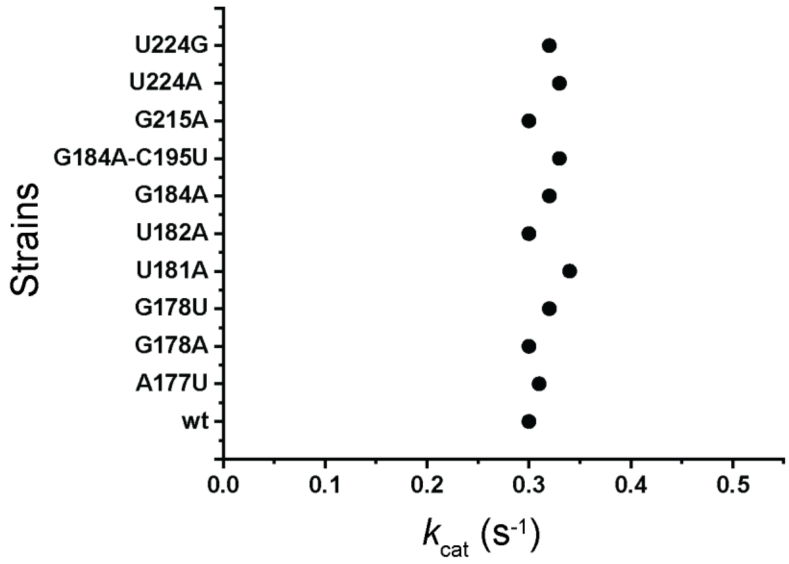

Supplementary Figure 43. Co-degradation rate of SgrS-*ptsG* mRNA complex for wild-type and the mutants. k_{cat} measured from the time dependent modeling curves of the SgrS, *ptsG* mRNA and SgrS-*ptsG* mRNA complexes for the wild-type and strains A177U, G178A, G178U, U181A, U182A, G184A, G184A-C195U, G215A, U224A, U224G. These were determined simultaneously in the wild-type and RNase E mutants. The plot shows mean values from the independent fitting on two replicates. Source data are provided as a Source Data file.

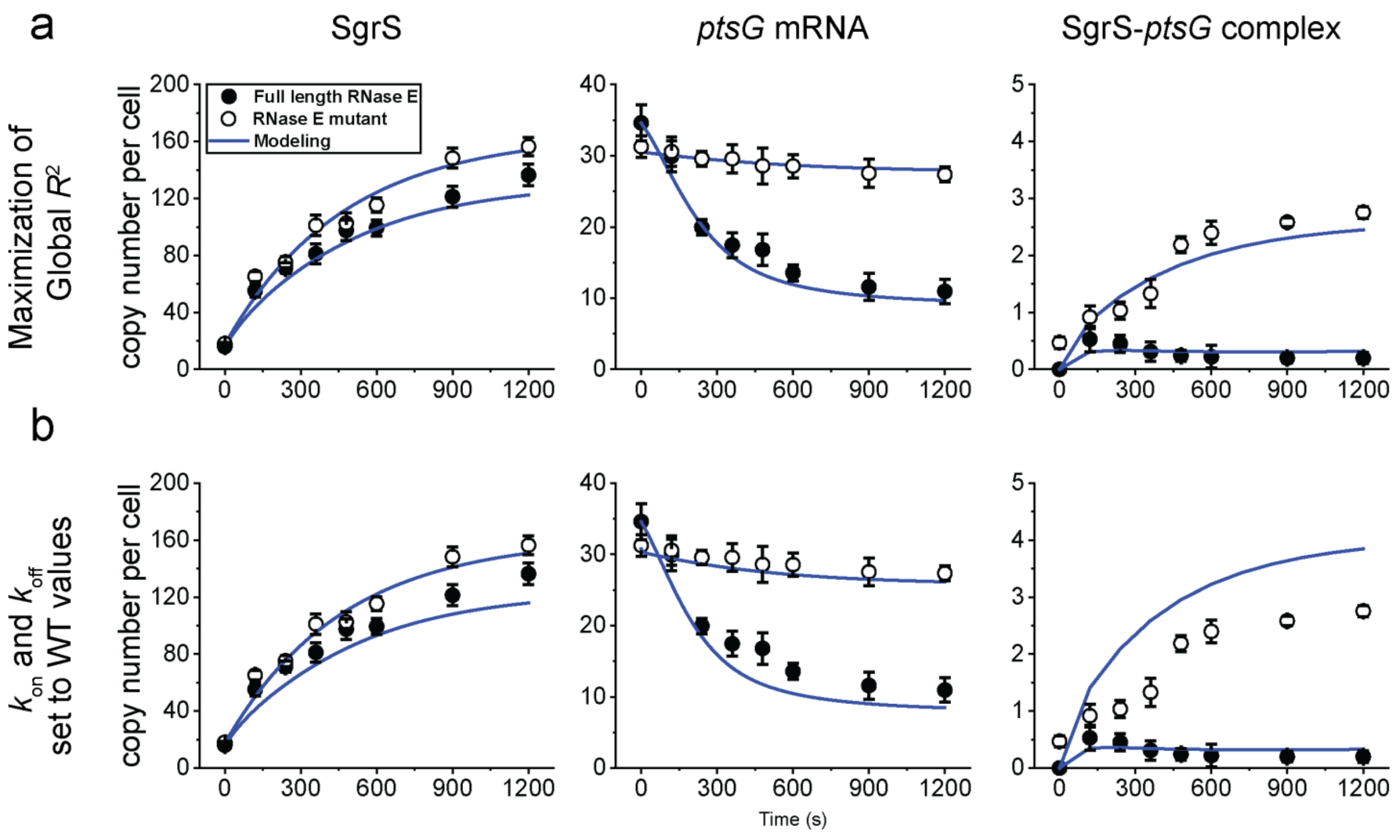

Supplementary Figure 44. Change in the copy numbers of SgrS and *ptsG* mRNA over time for SgrS A177U mutant strain. Time course changes from 0 min (0 s) to 20 min (1200 s) after the induction of glucose-phosphate stress using αMG (non-metabolizable sugar analog) and the corresponding modeling curves for SgrS, *ptsG* mRNA and SgrS-*ptsG* complexes for SgrS A177U strain by **(a)** maximization of global R^2 and **(b)** setting k_{on} and k_{off} to WT values. The average copy numbers per cell are plotted over time in each case. Rate constants obtained from (a) are shown in Fig. 6b, c, d and in Supplementary Table 3. Weighted R^2's for the modeling curves are reported in Supplementary Table 4. Data are presented as mean values +/- SEM in (a), (b) from n=132, 81, 88, 80, 85, 88, 86, 80 cells examined over 2 independent experiments after 0, 2, 4, 6, 8, 10, 15, 20 minutes induction respectively. Source data are provided as a Source Data file.

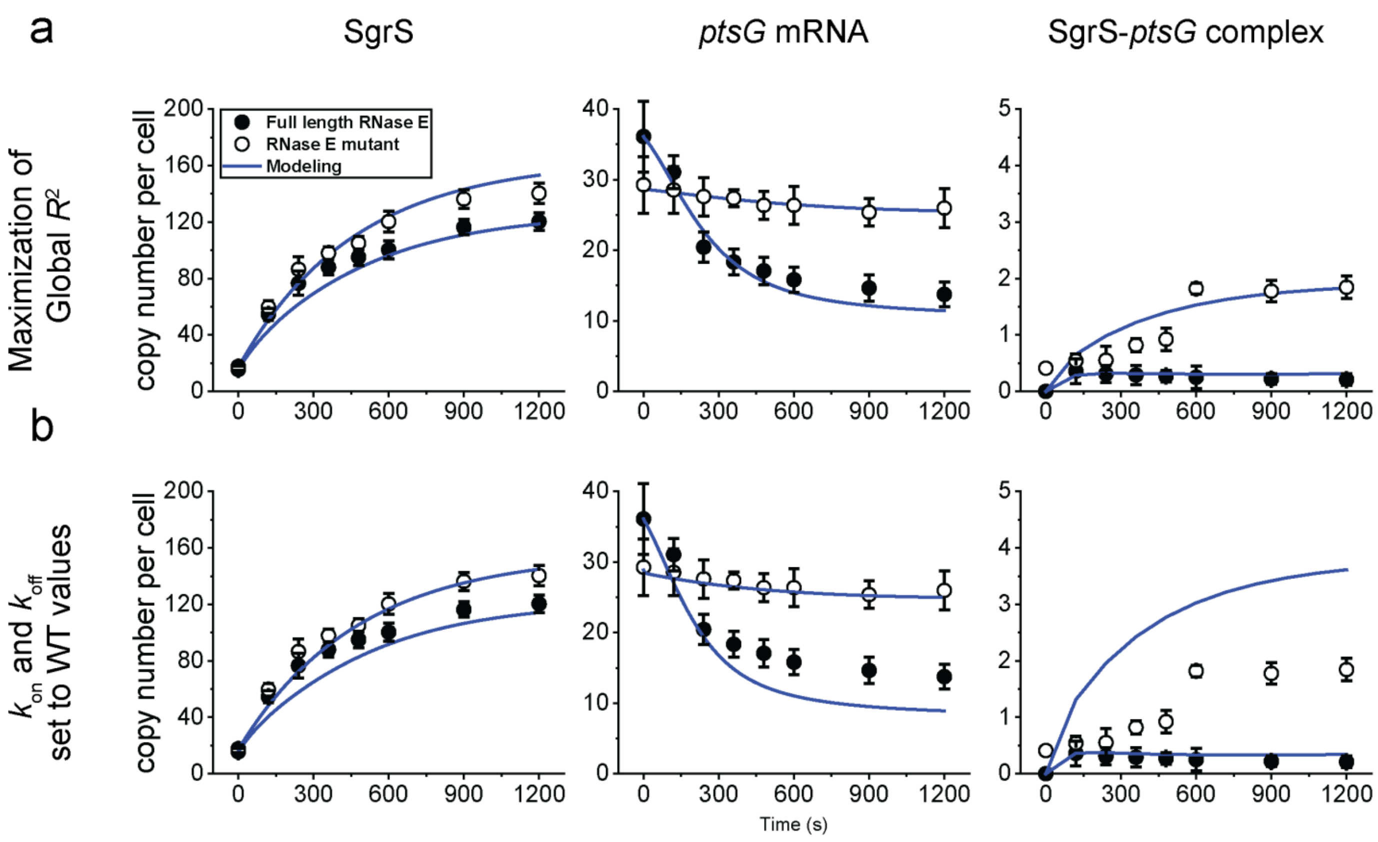

Supplementary Figure 45. Change in the copy numbers of SgrS and *ptsG* mRNA over time for SgrS G178A mutant strain. Time course changes from 0 min (0 s) to 20 min (1200 s) after the induction of glucose-phosphate stress using αMG (non-metabolizable sugar analog) and the corresponding modeling curves for SgrS, *ptsG* mRNA and SgrS-*ptsG* complexes for SgrS G178A strain by **(a)** maximization of global R^2 and **(b)** setting k_{on} and k_{off} to WT values. The average copy numbers per cell are plotted over time in each case. Rate constants obtained from (a) are shown in Fig. 6b, c, d and in Supplementary Table 3. Weighted R^2's for the modeling curves are reported in Supplementary Table 4. Data are presented as mean values +/- SEM in(a), (b) from n=81, 86, 89, 82, 87, 80, 83, 82 cells examined over 2 independent experiments after 0, 2, 4, 6, 8, 10, 15, 20 minutes induction respectively. Source data are provided as a Source Data file.

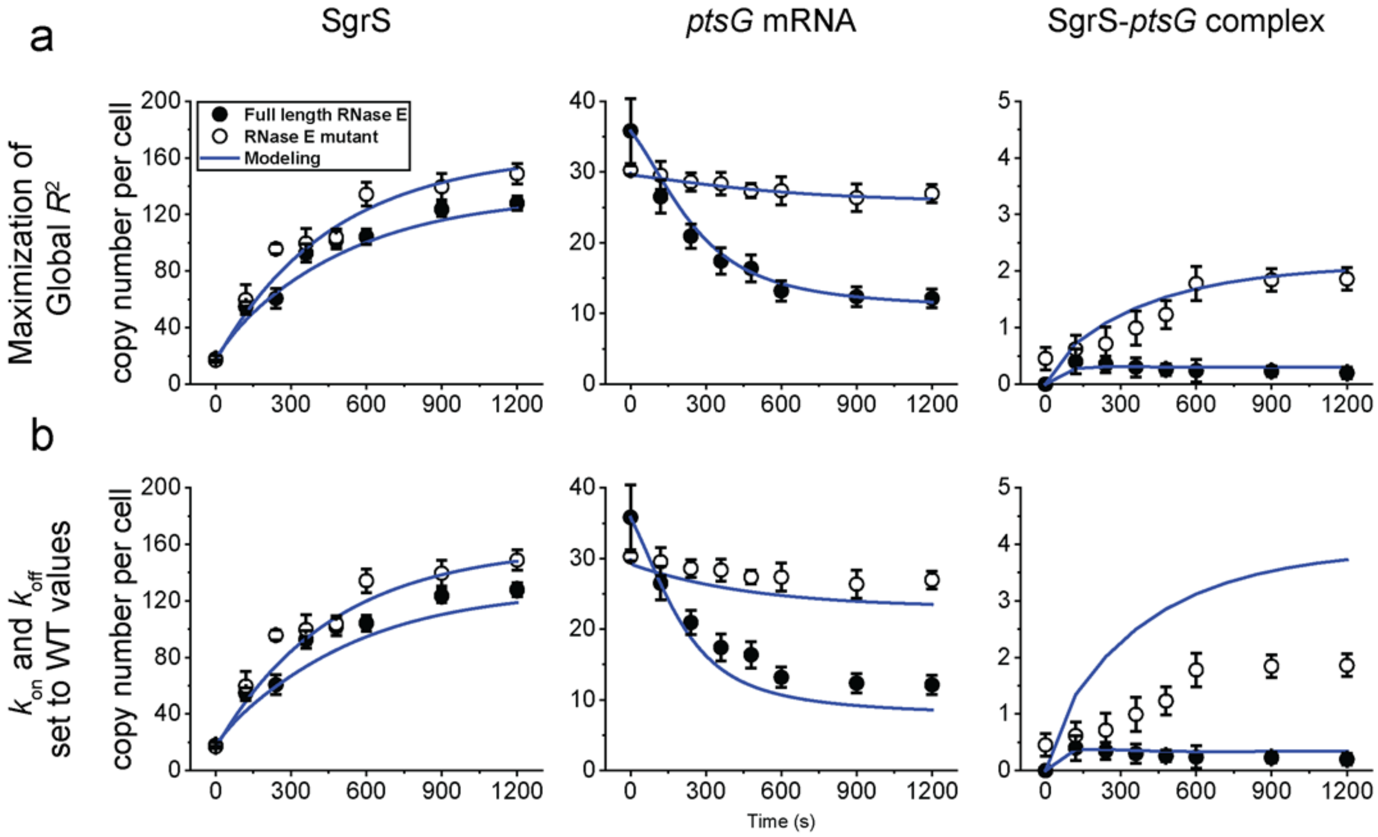

Supplementary Figure 46. Change in the copy numbers of SgrS and *ptsG* mRNA over time for SgrS G178U mutant strain. Time course changes from 0 min (0 s) to 20 min (1200 s) after the induction of glucose-phosphate stress using αMG (non-metabolizable sugar analog) and the corresponding modeling curves for SgrS, *ptsG* mRNA and SgrS-*ptsG* complexes for SgrS G178U strain by **(a)** maximization of global R^2 and **(b)** setting k_{on} and k_{off} to WT values. The average copy numbers per cell are plotted over time in each case. Rate constants obtained from (a) are shown in Fig. 6b, c, d and in Supplementary Table 3. Weighted R^2's for the modeling curves are reported in Supplementary Table 4. Data are presented as mean values +/- SEM in (a), (b) from n=88, 83, 97, 88, 90, 85, 86, 83 cells examined over 2 independent experiments after 0, 2, 4, 6, 8, 10, 15, 20 minutes induction respectively. Source data are provided as a Source Data file.

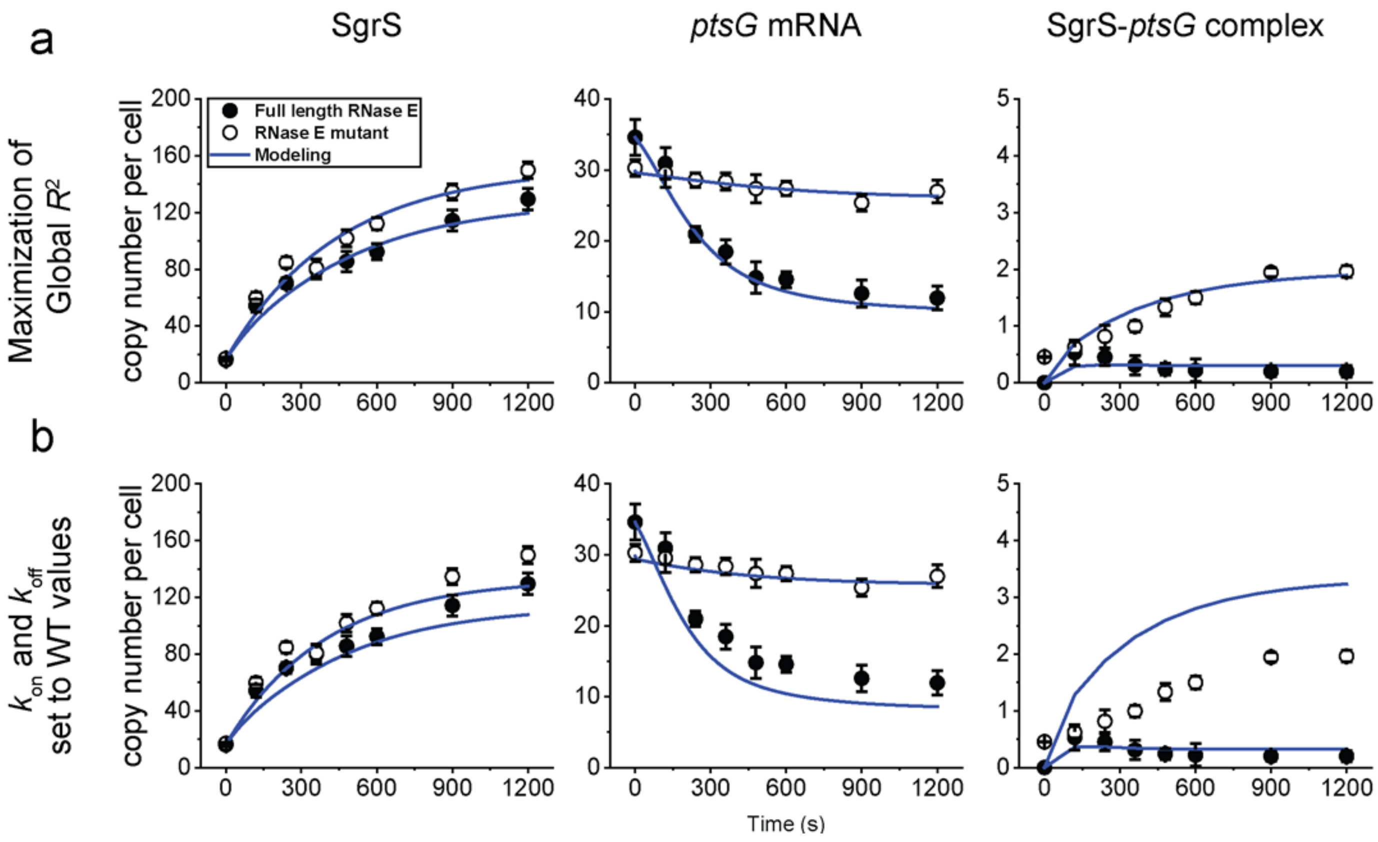

Supplementary Figure 47. Change in the copy numbers of SgrS and *ptsG* mRNA over time for SgrS U181A mutant strain. Time course changes from 0 min (0 s) to 20 min (1200 s) after the induction of glucose-phosphate stress using αMG (non-metabolizable sugar analog) and the corresponding modeling curves for SgrS, *ptsG* mRNA and SgrS-*ptsG* complexes for SgrS U181A strain by **(a)** maximization of global R^2 and **(b)** setting k_{on} and k_{off} to WT values. The average copy numbers per cell are plotted over time in each case. Rate constants obtained from (a) are shown in Fig. 6b, c, d and in Supplementary Table 3. Weighted R^2's for the modeling curves are reported in Supplementary Table 4. Data are presented as mean values +/- SEM in (a), (b) from n=91, 101, 85, 86, 90, 107, 95, 97 cells examined over 2 independent experiments after 0, 2, 4, 6, 8, 10, 15, 20 minutes induction respectively. Source data are provided as a Source Data file.

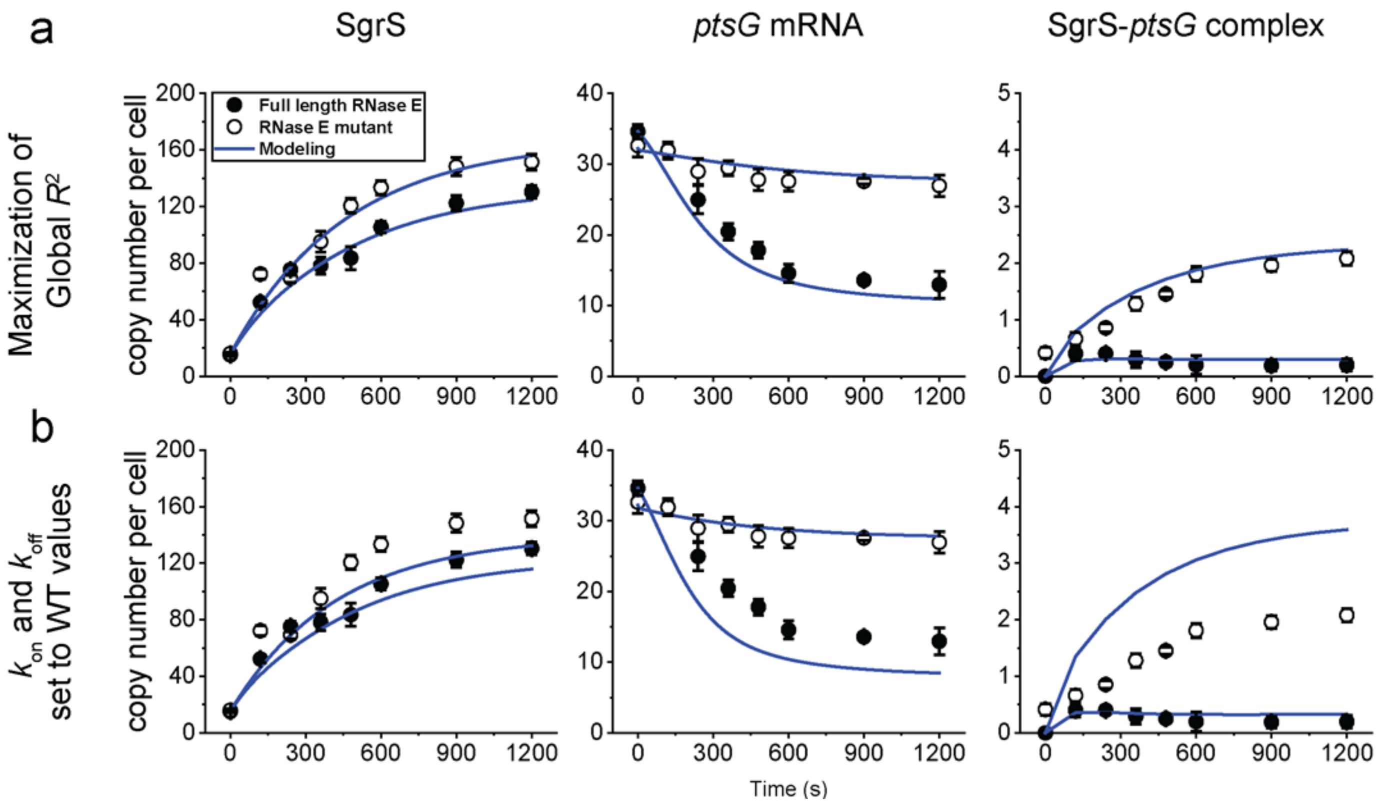

Supplementary Figure 48. Change in the copy numbers of SgrS and *ptsG* mRNA over time for SgrS U182A mutant strain. Time course changes from 0 min (0 s) to 20 min (1200 s) after the induction of glucose-phosphate stress using αMG (non-metabolizable sugar analog) and the corresponding modeling curves for SgrS, *ptsG* mRNA and SgrS-*ptsG* complexes for SgrS U182A strain by **(a)** maximization of global R^2 and **(b)** setting k_{on} and k_{off} to WT values. The average copy numbers per cell are plotted over time in each case. Rate constants obtained from (a) are shown in Fig. 6b, c, d and in Supplementary Table 3. Weighted R^2's for the modeling curves are reported in Supplementary Table 4. Data are presented as mean values +/- SEM in (a), (b) from n=101, 84, 87, 90, 112, 107, 82, 94 cells examined over 2 independent experiments after 0, 2, 4, 6, 8, 10, 15, 20 minutes induction respectively. Source data are provided as a Source Data file.

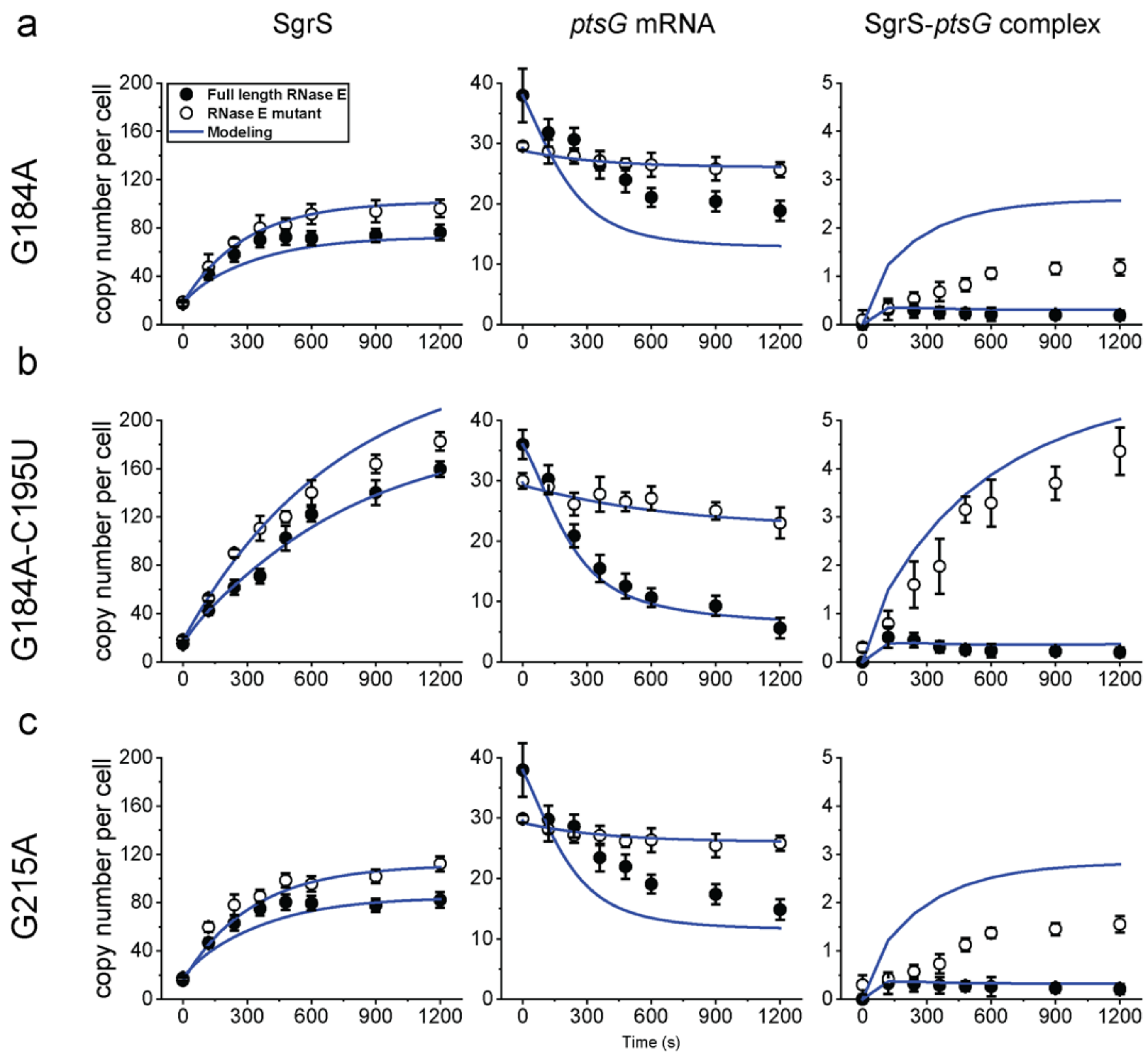

Supplementary Figure 49. Change in the copy numbers of SgrS and *ptsG* mRNA over time for SgrS mutant strains. Time course changes from 0 min (0 s) to 20 min (1200 s) after the induction of glucose-phosphate stress using αMG (non-metabolizable sugar analog) and the corresponding modeling curves for SgrS, *ptsG* mRNA and SgrS-*ptsG* complexes for (a) SgrS G184A strain, (b) SgrS G184A-C195U strain, (c) SgrS G215A strain by setting k_{on} and k_{off} to WT values. The average copy numbers per cell are plotted over time in each case. Weighted R^2's

for the modeling curves are reported in Supplementary Table 4. Data are presented as mean values +/- SEM in (a), (b), (c) from n=84, 84, 94, 99, 94, 90, 87, 88 cells examined over 2 independent experiments after 0, 2, 4, 6, 8, 10, 15, 20 minutes induction respectively for G184A mutant strain; for n=117, 101, 115, 111, 102, 110, 104, 104 cells examined over 2 independent experiments after 0, 2, 4, 6, 8, 10, 15, 20 minutes induction respectively for G184A-C195U mutant strain and for n=82, 88, 94, 104, 94, 90, 89, 88 cells examined over 2 independent experiments after 0, 2, 4, 6, 8, 10, 15, 20 minutes induction respectively for G215A mutant strain. Source data are provided as a Source Data file.

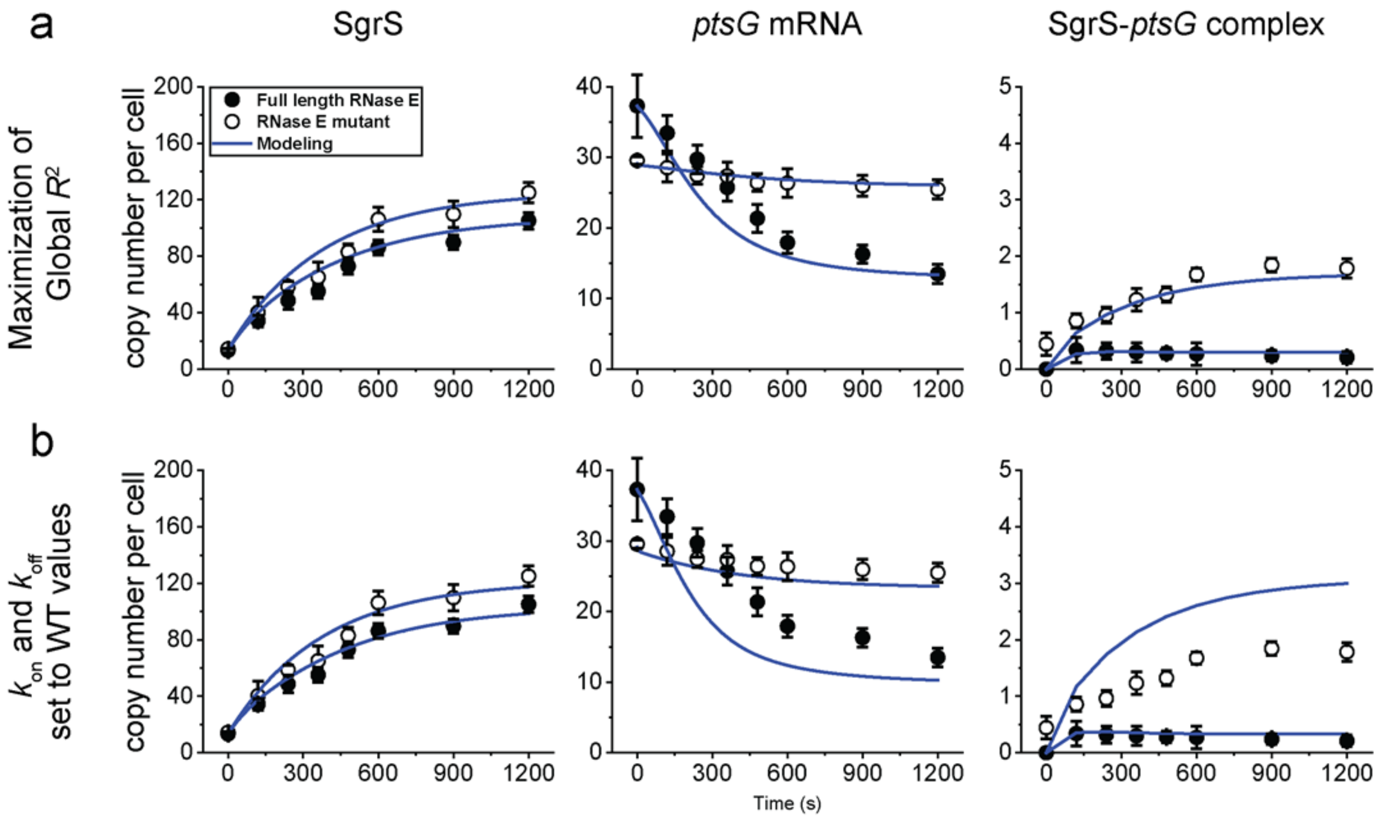

Supplementary Figure 50. Change in the copy numbers of SgrS and *ptsG* mRNA over time for SgrS U224A mutant strain. Time course changes from 0 min (0 s) to 20 min (1200 s) after the induction of glucose-phosphate stress using αMG (non-metabolizable sugar analog) and the corresponding modeling curves for SgrS, *ptsG* mRNA and SgrS-*ptsG* complexes for SgrS U224A strain by **(a)** maximization of global R^2 and **(b)** setting k_{on} and k_{off} to WT values. The average copy numbers per cell are plotted over time in each case. Rate constants obtained from (a) are shown in Fig. 6b, c, d and in Supplementary Table 3. Weighted R^2's for the modeling curves are reported in Supplementary Table 4. Data are presented as mean values +/- SEM in (a), (b) from n=90, 99, 99, 82, 83, 81, 81, 84 cells examined over 2 independent experiments after 0, 2, 4, 6, 8, 10, 15, 20 minutes induction respectively. Source data are provided as a Source Data file.

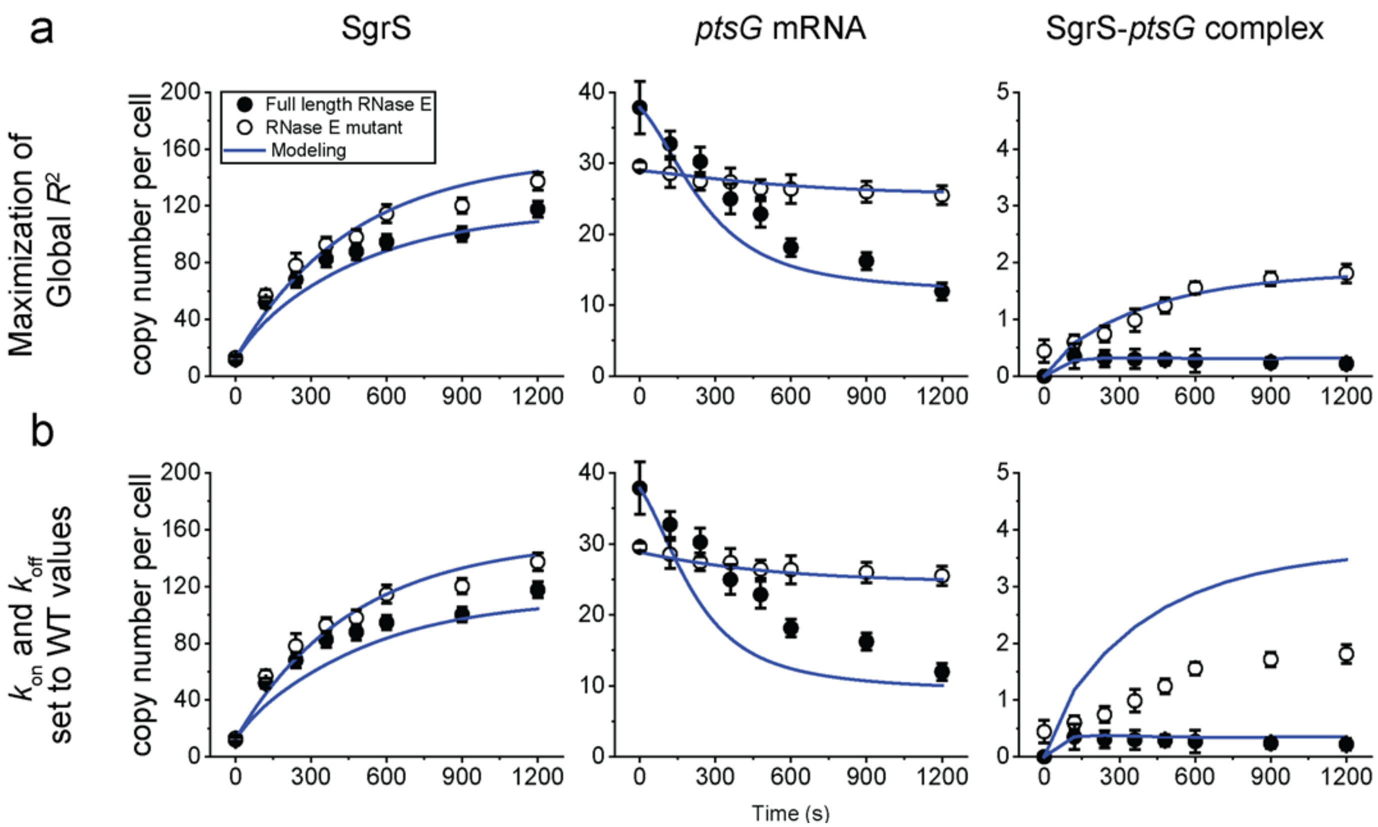

Supplementary Figure 51. Change in the copy numbers of SgrS and *ptsG* mRNA over time for SgrS U224G mutant strain. Time course changes from 0 min (0 s) to 20 min (1200 s) after the induction of glucose-phosphate stress using αMG (non-metabolizable sugar analog) and the corresponding modeling curves for SgrS, *ptsG* mRNA and SgrS-*ptsG* complexes for SgrS U224G strain by **(a)** maximization of global R^2 and **(b)** setting k_{on} and k_{off} to WT values. The average copy numbers per cell are plotted over time in each case. Rate constants obtained from (a) are shown in Fig. 6b, c, d and in Supplementary Table 3. Weighted R^2's for the modeling curves are reported in Supplementary Table 4. Data are presented as mean values +/- SEM in (a), (b) from n=92, 102, 105, 108, 83, 91, 94, 100 cells examined over 2 independent experiments after 0, 2, 4, 6, 8, 10, 15, 20 minutes induction respectively. Source data are provided as a Source Data file.

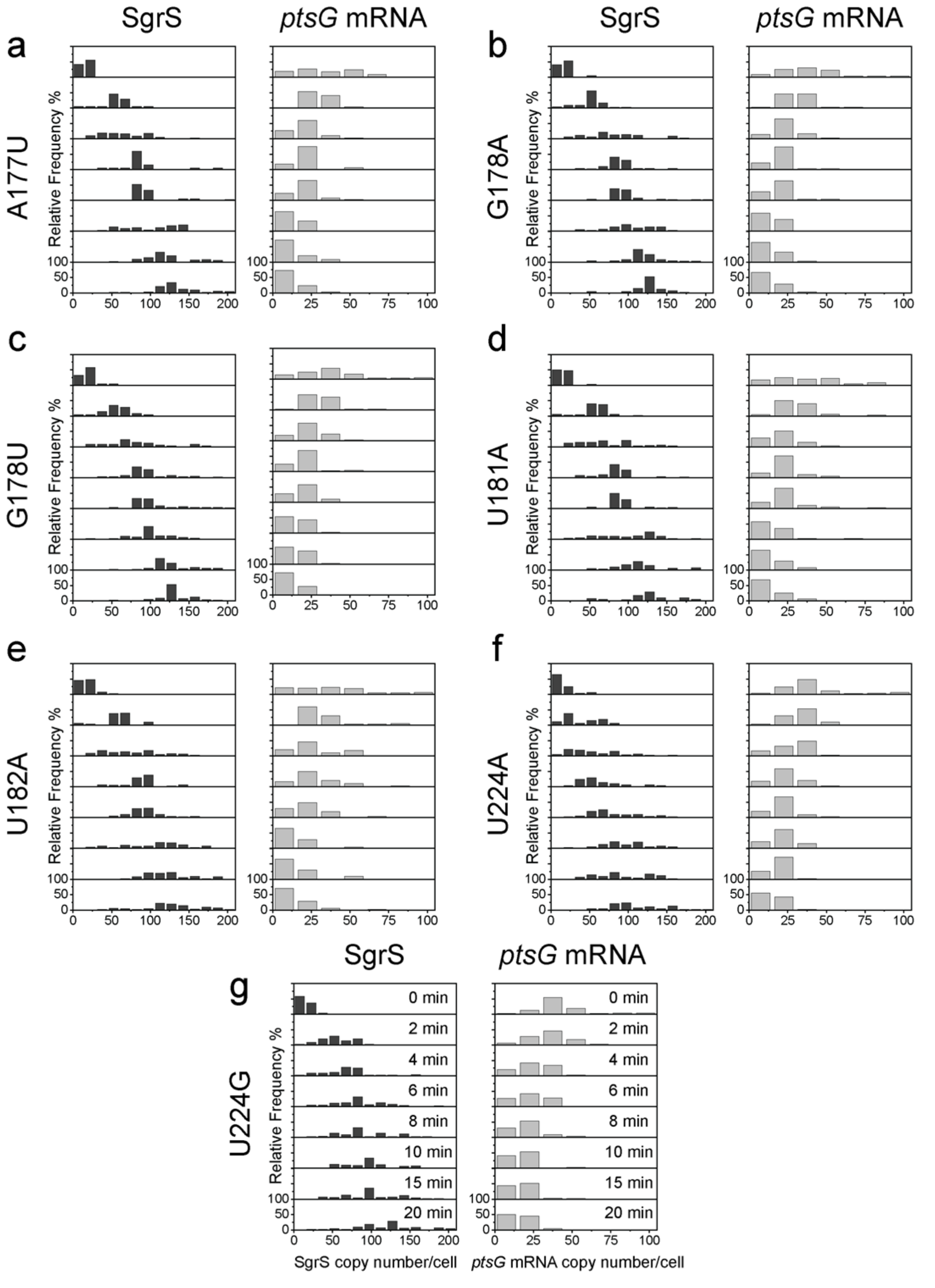

Supplementary Figure 52. Time dependent changes in the copy numbers of SgrS and ptsG mRNA. Histograms showing the change in distribution of SgrS and *ptsG* mRNA copy numbers

for (a) n=132, 81, 88, 80, 85, 88, 86, 80 cells examined over 2 independent experiments after 0, 2, 4, 6, 8, 10, 15, 20 minutes induction respectively for SgrS A177U mutant strain, (b) n=81, 86, 89, 82, 87, 80, 83, 82 cells examined over 2 independent experiments after 0, 2, 4, 6, 8, 10, 15, 20 minutes induction respectively for G178A mutant strain, (c) n=88, 83, 97, 88, 90, 85, 86, 83 cells examined over 2 independent experiments after 0, 2, 4, 6, 8, 10, 15, 20 minutes induction respectively for G178U mutant strain, (d) n=91, 101, 85, 86, 90, 107, 95, 97 cells examined over 2 independent experiments after 0, 2, 4, 6, 8, 10, 15, 20 minutes induction respectively for U181A mutant strain, (e) n=101, 84, 87, 90, 112, 107, 82, 94 cells examined over 2 independent experiments after 0, 2, 4, 6, 8, 10, 15, 20 minutes induction respectively for U182A mutant strain, (f) n=90, 99, 99, 82, 83, 81, 81, 84 cells examined over 2 independent experiments after 0, 2, 4, 6, 8, 10, 15, 20 minutes induction respectively for U224A mutant strain, (g) n=92, 102, 105, 108, 83, 91, 94, 100 cells examined over 2 independent experiments after 0, 2, 4, 6, 8, 10, 15, 20 minutes induction respectively for U224G mutant strain. Source data are provided as a Source Data file.

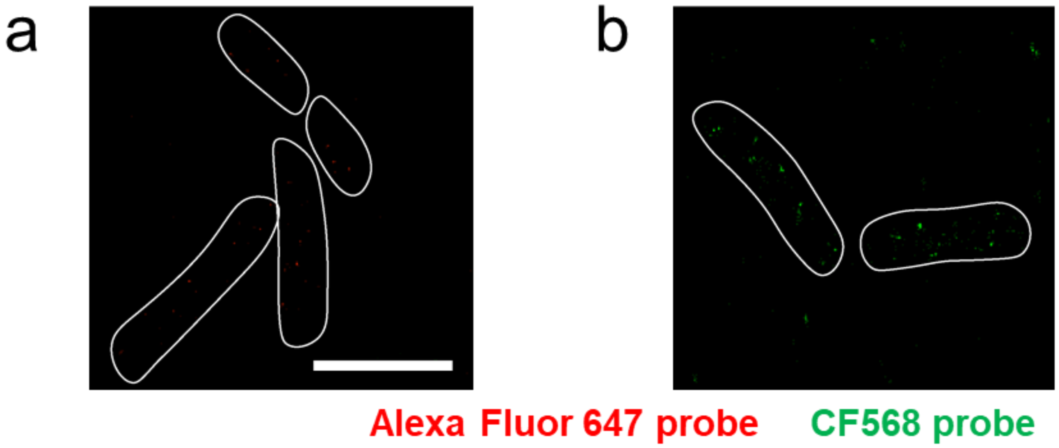

Supplementary Figure 53. Negative control for copy number calculation. (a) Background due to the non-specific binding of Alexa Fluor 647-labeled probes against SgrS in Δ*sgrS* strain. This experiment was performed independently 2 times. **(b)** Background due to the non-specific binding of CF568-labeled probes against *ptsG* mRNA in Δ*ptsG* strain. This experiment was performed independently 2 times. Scale bar is 2 μm and applies to all the panels.

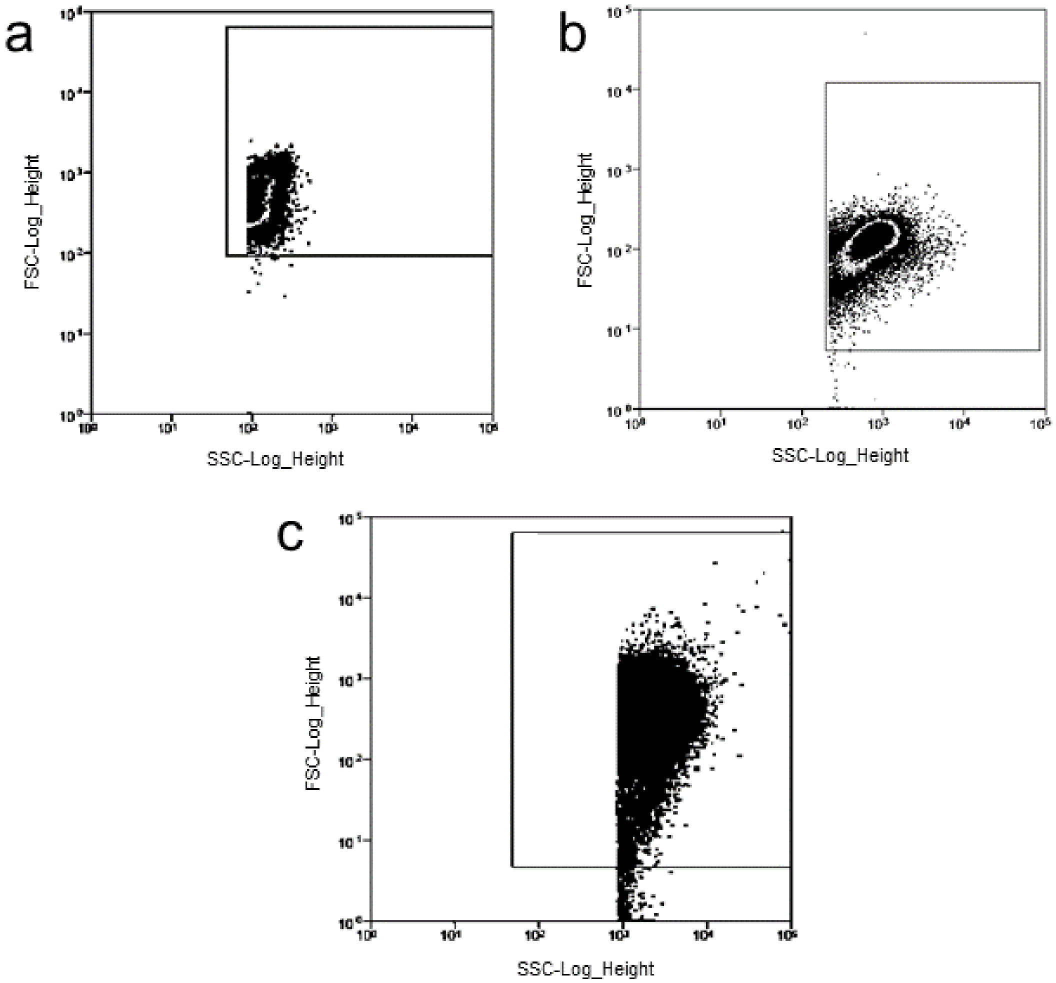

Supplementary Figure 54. FACS gating strategies used for cell sorting. (a) Gating strategy used for *E. coli* cells with target (*ptsG*)-only (green curve) shown in Fig. 2b. This experiment was performed independently 2 times. **(b)** Gating strategy used for *E. coli* cells with wild-type SgrS along with target (*ptsG*) (black curve) shown in Fig. 2b. This experiment was performed independently 2 times. **(c)** Gating strategy used for *E. coli* cells with SgrS library along with target (*ptsG*) (blue curve) shown in Fig. 2b. These cells were sorted into 5 evenly spaced (log scale) fluorescence bins and those details are presented in Fig. 2b itself. This experiment was performed independently 2 times.

Supplementary Tables

Supplementary Table 1. Plasmids and strains used in this study.

**Plasmid**	**Background**	**Source or Reference**
pSIM6	P_L -*gam-bet-exo* genes under the control of CI857 repressor, Amp^R (Ts)	Don Court
pZAMB1	$P_{Ltet0-1}$-*sgrS*	[3]
pZEMB8	$P_{Llac0-1}$-*ptsG-gfpsf*	[3]
pZAMB1A177T	$P_{Ltet0-1}$-*sgrS* A177T	This work
pZAMB1G178T	$P_{Ltet0-1}$-*sgrS* G178T	This work
pZAMB1G178A	$P_{Ltet0-1}$-*sgrS* G178A	This work
pZAMB1G184A	$P_{Ltet0-1}$-*sgrS* G184A	This work
pZAMB1C215A	$P_{Ltet0-1}$-*sgrS* C215A	This work
pZAMB1T224G	$P_{Ltet0-1}$-*sgrS* T224G	This work
pZAMB1T224A	$P_{Ltet0-1}$-*sgrS* T224A	This work
**Strain**	**Background**	**Source or Reference**
DJ480	MG1655 Δ*lac* X74	D. Jin, NCI
CV104	DJ480 Δ*lac* X74, *mal::lacI*^{q}, Δ*sgrS::kan*^{R}	C. K. Vanderpool, S. Gottesman, 2004
DB166	MG1655 ΔX74*lac, lacI*^{q}*-tetR-spec*^{R}	Divya Balasubramanian
MB1	Δ*ptsG,* Δ*sgrS, lacI*^{q}*-tetR-spec*^{R}	This work
MB130	lacI'::P_{BAD}-*ptsG'-'lacZ, λattB::lacI*^{q}*-PN25tetR-spec*^{R}*,* miniλ*tet*^{R}, Δ*araBAD araC*$^+$, *mal::lacI*^{q}	This work
MB205	*ΔsgrS::cat-sacB, lacI*^{q}*-tetR-spec*^{R}	This work
MB206	*sgrS* A177T, *lacI*^{q}*-tetR-spec*^{R}	This work

MB207	*sgrS* G178T, *lacI^q-tetR-spec^R*	This work
MB208	*sgrS* G178A, *lacI^q-tetR-spec^R*	This work
MB209	*sgrS* G184A, *lacI^q-tetR-spec^R*	This work
SA1701	*sgrS* G215A, *lacI^q-tetR-spec^R*	This work
XM180	*sgrS* G184A-C195T, *lacI^q-tetR-spec^R*	This work
XM181	*sgrS* T181A, *lacI^q-tetR-spec^R*	This work
XM182	*sgrS* T182A, *lacI^q-tetR-spec^R*	This work
SA1708	*sgrS* T224A, *lacI^q-tetR-spec^R*	This work
SA1709	*sgrS* T224G, *lacI^q-tetR-spec^R*	This work
TM528	*rne701-FLAG-cat*	[4]
SA1740	*sgrS* A177T, *lacI^q-tetR-spec^R, rne131::kan^R*	This work
SA1741	*sgrS* A178T, *lacI^q-tetR-spec^R, rne131::kan^R*	This work
SA1742	*sgrS* A178A, *lacI^q-tetR-spec^R, rne131::kan^R*	This work
SA1743	*sgrS* A184A, *lacI^q-tetR-spec^R, rne131::kan^R*	This work
SA1744	*sgrS* G215A, *lacI^q-tetR-spec^R, rne131::kan^R*	This work
SA1745	*sgrS* T224G, *lacI^q-tetR-spec^R, rne131::kan^R*	This work
SA1746	*sgrS* T224A, *lacI^q-tetR-spec^R, rne131::kan^R*	This work
SA1908	*sgrS* G184A-C195T, *lacI^q-tetR-spec^R, rne131::kan^R*	This work
SA1909	*sgrS* T181A, *lacI^q-tetR-spec^R, rne131::kan^R*	This work
SA1910	*sgrS* T182A, *lacI^q-tetR-spec^R, rne131::kan^R*	This work
XM199	*lacI^q-tetR-spec^R, Δhfq::kan^R*	This work
XM200	*sgrS* A177T, *lacI^q-tetR-spec^R, Δhfq::kan^R*	This work

XM201	*sgrS* G184A, *lacI*^{q}*-tetR-spec*^{R}*, Δhfq::kan*^{R}	This work
CS196	*ΔptsG*::*tet*^{R}	[5]
CS123	*DJ480 strain carrying G178C/G176C mutations on sgrS*	[6,7]

Supplementary Table 2. Oligonucleotides used in this study.

Oligo	Description	Sequence 5'-3'
1	Forward Primer for mutagenesis PCR of SgrS	CTATCAGTGATAGAGATACTGAGCACATATG
2	Reverse Primer for mutagenesis PCR of SgrS	GAGCCTTTCGTTTTATTTGATGGATCC
3	Forward primer for sequencing desired region of SgrS	TGGGATGACCGCAATTCTGAAA
4	Reverse primer for sequencing desired region of SgrS	GAGCCTTTCGTTTTATTTGATGGATCC
MBP251F	Forward primer for PCR amplification of the *cat-sacB* cassette	GCAATTTTATTTTCCCTATATTAAGTCAATAATTCCTAACAAA ATGAGACGTTGATCGGCACGTAAG
MBP251R	Reverse primer for PCR amplification of the *cat-sacB* cassette	TTATCCAGATCATACGTTCCCTTTTTAGCGCGGCGAGAATGTA TCAAAGGGAAACTGTCCATATGC
OSA766	Forward primer for PCR amplification of *sgrS*, for allelic exchange between *sgrS* and the *cat-sacB* cassette	GCAATTTTATTTTCCCTATATTAAGTCAATAATTCCTAACgat GAAGCAAGGGGGTGCCC
MBP252R	Reverse primer for PCR	TTATCCAGATCATACGTTCCCTTTTTAGCGCGGCGAGAATAAA AAAAACCAGCAGGTATAATCTGCTGGCGGG

	amplification of *sgrS*, for allelic exchange between *sgrS* and the *cat-sacB* cassette	
OSA768	Reverse primer for PCR amplification of *sgrS* T181A, for allelic exchange between *sgrS* and the *cat-sacB* cassette	TTATCCAGATCATACGTTCCCTTTTTAGCGCGGCGAGAATaaa AAAAACCAGCAGGTATAATCTGCTGGCGGGTGATTTTACACCA TTACTCAGTCACACATGATGCAGGCA
OSA769	Reverse primer for PCR amplification of *sgrS* T182A, for allelic exchange between *sgrS* and the *cat-sacB* cassette	TTATCCAGATCATACGTTCCCTTTTTAGCGCGGCGAGAATaaa AAAAACCAGCAGGTATAATCTGCTGGCGGGTGATTTTACACCT ATACTCAGTCACACATGATGCAGGC
MBP252R2	Reverse primer for PCR amplification of *sgrS* G215A, for allelic exchange between *sgrS* and the *cat-sacB* cassette	TTATCCAGATCATACGTTCCCTTTTTAGCGCGGCGAGAATAAA AAAAACCAGTAGGTATAATCTGCTGGCGGG
MBP252R3	Reverse primer for PCR amplification of *sgrS* T224G, for allelic exchange between *sgrS*	TTATCCAGATCATACGTTCCCTTTTTAGCGCGGCGAGAATAAA CAAAACCAGTAGGTATAATCTGCTGGCGGG

	and the *cat-sacB* cassette	
MBP252R4	Reverse primer for PCR amplification of *sgrS* T224A, for allelic exchange between *sgrS* and the *cat-sacB* cassette	TTATCCAGATCATACGTTCCCTTTTTAGCGCGGCGAGAATAAA TAAAACCAGTAGGTATAATCTGCTGGCGGG
OSA499	Primer to amplify SgrS	GATGAAGCAAGGGGGTGCCC
OSA500	Primer to amplify SgrS	CAATACTCAGTCACACATGATGCAGGC
OXM187	Primer to amplify *rrsA*	ATTCCGATTAACGCTTGCAC
OXM188	Primer to amplify *rrsA*	AGGCCTTCGGGTTGTAAAGT
MBP201F		ACCTGACGCTTTTTATCGCAACTCTCTACTGTTTCTCCATATAA ATAAAGGGCGCTTAGATGCCCTGTAC
MBP201R		TAACGCCAGGTTTTCCCAGTCACGACGTTGTAAAACGACTTGC AGGTTAGCAAATGCATTCTTAAACAT
A177T-F	Forward primer to generate SgrS A177T mutant	CTTGCCTGCATCATGTGTGACTGTGTATTGGTGTAAAATC
A177T-F	Reverse primer to generate SgrS A177T mutant	GATTTTACACCAATACACAGTCACACATGATGCAGGCAA
G178T-F	Forward primer to generate SgrS G178T mutant	CTTGCCTGCATCATGTGTGACTGATTATTGGTGTAAAATC
G178T-R	Reverse primer to generate SgrS G178T mutant	GATTTTACACCAATAATCAGTCACACATGATGCAGGCAAG

G178A-F	Forward primer to generate SgrS G178A mutant	CTTGCCTGCATCATGTGTGACTGAATATTGGTGTAAAATC
G178A-R	Reverse primer to generate SgrS G178A mutant	GATTTTACACCAATTATCAGTCACACATGATGCAGGCAAG
G184A-F	Forward primer to generate SgrS G184A mutant	CCTGCATCATGTGTGACTGAGTATTGATGTAAAATCACCC
G184A-R	Reverse primer to generate SgrS G184A mutant	GGGTGATTTTACATCAATACTCAGTCACACATGATGCAGG
C215A-F	Forward primer to generate SgrS C215A mutant	CCCGCCAGCAGATTATACCTACTGGTTTTTTTTATTCTC
C215A-R	Reverse primer to generate SgrS C215A mutant	GAGAATAAAAAAAACCAGTAGGTATAATCTGCTGGCGGG
T224G-F	Forward primer to generate SgrS T224G mutant	GATTATACCTGCTGGTTTTATTTATTCTCGCCGCG
T224G-R	Reverse primer to generate SgrS T224G mutant	CGCGGCGAGAATAAATAAAACCAGCAGGTATAATC
T224A-F	Forward primer to generate SgrS T224A mutant	GATTATACCTGCTGGTTTTGTTTATTCTCGCCGCG
T224A-R	Reverse primer to generate SgrS T224A mutant	CGCGGCGAGAATAAACAAAACCAGCAGGTATAATC
sgrS-bio	SgrS probe used in the Northern blot analysis	GCAACCAGCACAACTTCGCTGTCGCGGTAAAATAGTG
ssrA-bio	5S rRNA probe used in Northern blot analysis	CGCCACTAACAAACTAGCCTGACGCCACTAACAAA

SgrS	Probes* for smFISH	GTGCTGATAAAACTGACGCA<br>ACTTCGCTGTCGCGGTAAAA<br>CTTAACCAACGCAACCAGCA<br>CATGGTTAATCGTTGTGGGA<br>ATCCCACTGCATCAGTCCTT<br>GTCAACTTTCAGAATTGCGG<br>TCAGTCACACATGATGCAGG<br>GCGGGTGATTTTACACCAAT<br>AACCAGCAGGTATAATCTGC
ptsG	Probes* for smFISH	GCATCTAAGCGCCCTTTATT<br>GCAGGTTAGCAAATGCATTC<br>ATCAGCGATTTACCGACCTT<br>CTGCCATAACATGCGATACA<br>CATGTTTGCAAAGACGGAAC<br>GACACCGATCGCAAAAATCA<br>GATACGCCATCGTTATTGGT<br>ATGATGCCATAGGCAACAAC<br>AACCACGGCCATGGTTTTAA<br>CAGGTGTTTAGAGGCGATTT<br>TAAACATGTACGCTGCGATC<br>GGCAGCTTAATACGGTAGAA<br>GGCAAAGAAGCCAAGATACT<br>CAGAAATGATCGGCACAAAG<br>CCAAATGAAGGACAGCACAA<br>ACTGAGAGAAGGTCTGGATT<br>CAACGTTCGATGAAACCGTA<br>GGTGTATTCACCAATCTGCA<br>GGCAGACCGTACATTTTGAA<br>CGGTTTTCTGGTTTAGCAGA<br>AACGATGAAGTCGATCAGAC<br>GCGGAAGATGGTGTAGTAAA<br>CGTTTTCAGATCCAGTGCTT<br>TCGCTTTTGCATCTTCAGTC<br>TCTTTACCACCAAATGCAGC<br>TACATGCGTCGAGGTTAGTA<br>TTAGTACCGAAAATCGCCTG<br>AGTGGTTACGGATGTACTCA

*The probes were complementary sequences across the length of the RNAs.

Supplementary Table 3. Rate constants for SgrS, *ptsG* mRNA association, dissociation and SgrS-*ptsG* complex codegradation.

	α_s (molecules-s^{-1})	β_s (s^{-1})	α_p (molecules-s^{-1})	β_p (s^{-1})	k_{on} (M^{-1}-s^{-1}) x 10^5	k_{off} (s^{-1})	k_{cat} (s^{-1})
**Wild Type**	0.35	0.0013	0.13 ± 0.02	0.0037	1.9	0.22	0.3
**Wild Type RNase E Mutant**	0.35	0.0013	0.11 ± 0.02	0.0037	1.9	0.22	0.0062
**A177U**	0.36	0.0021	0.14 ± 0.02	0.0039	1.5	0.25	0.31
**A177U RNase E Mutant**	0.36	0.0021	0.12 ± 0.02	0.0039	1.5	0.25	0.0060
**G178A**	0.36	0.0021	0.14 ± 0.03	0.0038	1.3	0.27	0.3
**G178A RNase E Mutant**	0.36	0.0021	0.11 ± 0.03	0.0038	1.3	0.27	0.0078
**G178U**	0.36	0.0021	0.14 ± 0.03	0.0039	1.3	0.27	0.32
**G178U RNase E Mutant**	0.36	0.0021	0.12 ± 0.02	0.0039	1.3	0.27	0.0075
**U181A**	0.36	0.0022	0.13 ± 0.02	0.0037	1.4	0.26	0.34
**U181A RNase E Mutant**	0.36	0.0022	0.11 ± 0.02	0.0037	1.4	0.26	0.0065
**U182A**	0.34	0.0023	0.14 ± 0.02	0.0040	1.4	0.265	0.3
**U182A RNase E Mutant**	0.34	0.0023	0.13 ± 0.02	0.0040	1.4	0.265	0.0060

**G184A**	0.35	0.0034	0.15 ± 0.03	0.0040	0.95	0.293	0.32
**G184A RNase E Mutant**	0.35	0.0034	0.12 ± 0.02	0.0040	0.95	0.293	0.0080
**G184A-C195U**	0.35	0.0014	0.14 ± 0.03	0.0039	1.85	0.225	0.33
**G184A-C195U RNase E Mutant**	0.35	0.0014	0.12 ± 0.02	0.0039	1.85	0.225	0.0068
**G215A**	0.36	0.0025	0.15 ± 0.02	0.0040	1.2	0.28	0.30
**G215A RNase E Mutant**	0.36	0.0025	0.12 ± 0.02	0.0040	1.2	0.28	0.0082
**U224A**	0.34	0.0024	0.14 ± 0.03	0.0038	1.28	0.258	0.33
**U224A RNase E Mutant**	0.34	0.0024	0.11 ± 0.02	0.0038	1.28	0.258	0.0080
**U224G**	0.35	0.0022	0.14 ± 0.02	0.0038	1.3	0.27	0.32
**U224G RNase E Mutant**	0.35	0.0022	0.11 ± 0.02	0.0038	1.3	0.27	0.0075

Supplementary Table 4. Goodness of fit for the time dependent curves.

	**Global R^2**	**R^2 for SgrS**	**R^2 for ptsG**	**R^2 for complex**	**χ^2 for complex**	**α for complex**
**Wild-type**	0.999	0.999	0.995	-	0.37	0.01
**Wild-type RNase E Mutant**	0.999	0.999	0.963	0.902	-	-
**A177U**	0.998	0.998	0.993	-	0.33	0.001
**A177U with WT k_{on} and k_{off}**	0.990	0.996	0.970	-	0.27	0.001
**A177U RNase E Mutant**	0.998	0.999	0.927	0.805	-	-
**A177U RNase E Mutant with WT k_{on} and k_{off}**	0.990	0.998	0.796	0.581	-	-
**G178A**	0.999	0.999	0.990	-	0.09	0.001
**G178A with WT k_{on} and k_{off}**	0.990	0.993	0.931	-	0.15	0.001
**G178A RNase E Mutant**	0.999	0.999	0.962	0.700	-	-
**G178A RNase E Mutant with WT k_{on} and k_{off}**	0.990	0.998	0.749	-	17.73	0.99
**G178U**	0.999	0.999	0.994	-	0.13	0.001
**G178U with WT k_{on} and k_{off}**	0.991	0.994	0.959	-	0.15	0.001
**G178U RNase E Mutant**	0.999	0.999	0.970	0.795	-	-
**G178U RNase E Mutant with WT k_{on} and k_{off}**	0.991	0.999	0.772	-	21.36	0.999
**U181A**	0.998	0.998	0.997	-	0.27	0.001
**U181A with WT k_{on} and k_{off}**	0.982	0.982	0.979	-	2.63	0.15
**U181A RNase E Mutant**	0.998	0.998	0.989	0.789	-	-
**U181A RNase E Mutant with WT k_{on} and k_{off}**	0.982	0.988	0.978	-	24.59	0.9995

**U182A**	0.997	0.998	0.986	-	0.69	0.01
**U182A with WT k_{on} and k_{off}**	0.987	0.995	0.968	-	2.57	0.15
**U182A RNase E Mutant**	0.997	0.997	0.990	0.854	-	-
**U182A RNase E Mutant with WT k_{on} and k_{off}**	0.987	0.994	0.975	-	17.26	0.99
**G184A**	0.998	0.998	0.996	-	0.12	0.001
**G184A with WT k_{on} and k_{off}**	0.982	0.987	0.837	-	0.18	0.001
**G184A RNase E Mutant**	0.998	0.999	0.997	0.704	-	-
**G184A RNase E Mutant with WT k_{on} and k_{off}**	0.982	0.999	0.969	-	5.47	0.5
**G184A-C195U**	0.999	0.999	0.976	-	0.28	0.001
**G184A-C195U with WT k_{on} and k_{off}**	0.996	0.997	0.984	-	0.29	0.001
**G184A-C195U RNase E Mutant**	0.999	0.999	0.957	0.953	-	-
**G184A-C195U RNase E Mutant with WT k_{on} and k_{off}**	0.996	0.997	0.975	0.905	-	-
**G215A**	0.998	0.998	0.992	-	0.04	0.001
**G215A with WT k_{on} and k_{off}**	0.988	0.990	0.913	-	0.11	0.001
**G215A RNase E Mutant**	0.998	0.998	0.836	0.712	-	-
**G215A RNase E Mutant with WT k_{on} and k_{off}**	0.988	0.998	0.928	-	4.56	0.4
**U224A**	0.998	0.998	0.982	-	0.06	0.001
**U224A with WT k_{on} and k_{off}**	0.989	0.999	0.913	-	0.12	0.001
**U224A RNase E Mutant**	0.998	0.998	0.912	0.702	-	-
**U224A RNase E Mutant with WT k_{on} and k_{off}**	0.989	0.997	0.645	-	37.39	0.999995
**U224G**	0.998	0.998	0.964	-	0.08	0.001

**U224G with WT k_{on} and k_{off}**	0.988	0.991	0.888	-	0.13	0.001
**U224G RNase E Mutant**	0.998	0.998	0.933	0.941	-	-
**U224G RNase E Mutant with WT k_{on} and k_{off}**	0.988	0.997	0.981	-	5.731	0.55

Supplementary Note 1

Impact of SgrS mutations on *ptsG* mRNA intrinsic degradation rates

In order to solve the deterministic model (equations shown in Fig. 1), we also needed to obtain the SgrS-independent degradation rates of *ptsG* mRNA. To determine this in various SgrS mutant strains, we grew cells without SgrS induction and added rifampicin to stop transcription. Samples were collected over time and fixed using paraformaldehyde. The fixed cells were then subjected to the same imaging and analysis protocol and the average copy number of *ptsG* mRNA per cell was calculated as a function of time after transcription inhibition. The degradation rate thus determined was 0.0037 s^{-1} (4.5 min lifetime) for the wild type strain and remained similar for all the mutants (Fig. S24-S34, S39). This verified that the mutations in the SgrS by themselves do not affect the *ptsG* mRNA stability in the absence of sugar stress and co-degradation.

References

1. Aiba, H., Adhya, S. & de Crombrugghe, B. Evidence for two functional gal promoters in intact Escherichia coli cells. *J. Biol. Chem.* **256**, 11905–10 (1981).

2. Schneider, C. A., Rasband, W. S. & Eliceiri, K. W. NIH Image to ImageJ: 25 years of image analysis. *Nature Methods* (2012). doi:10.1038/nmeth.2089

3. Bobrovskyy, M. & Vanderpool, C. K. Diverse mechanisms of post-transcriptional repression by the small RNA regulator of glucose-phosphate stress. *Mol. Microbiol.* **99**, 254–273 (2016).

4. Morita, T., Kawamoto, H., Mizota, T., Inada, T. & Aiba, H. Enolase in the RNA degradosome plays a crucial role in the rapid decay of glucose transporter mRNA in the response to phosphosugar stress in Escherichia coli. *Mol. Microbiol.* **54**, 1063–1075 (2004).

5. Fei, J. *et al.* Determination of in vivo target search kinetics of regulatory noncoding RNA. *Science (80-.).* **347**, 1371–1374 (2015).

6. Kawamoto, H., Koide, Y., Morita, T. & Aiba, H. Base-pairing requirement for RNA silencing by a bacterial small RNA and acceleration of duplex formation by Hfq. *Mol. Microbiol.* **61**, 1013–1022 (2006).

7. Wadler, C. S. & Vanderpool, C. K. Characterization of homologs of the small rna sgrs reveals diversity in function. *Nucleic Acids Res.* **37**, 5477–5485 (2009).
